# Synthesis, potential antitumor activity, cell cycle analysis, and multitarget mechanisms of novel hydrazones incorporating a 4-methylsulfonylbenzene scaffold: a molecular docking study

**DOI:** 10.1080/14756366.2021.1924698

**Published:** 2021-07-15

**Authors:** Alaa A.-M. Abdel-Aziz, Adel S. El-Azab, Nawaf A. AlSaif, Ahmad J. Obaidullah, Abdulrahman M. Al-Obaid, Ibrahim A. Al-Suwaidan

**Affiliations:** Department of Pharmaceutical Chemistry, College of Pharmacy, King Saud University, Riyadh Saudi Arabia

**Keywords:** Hydrazones synthesis, antitumor activity, cell cycle analysis, enzymatic assay, COX-2 inhibition, EGFR inhibition, HER2 inhibition, molecular docking

## Abstract

Hydrazone is a bioactive pharmacophore that can be used to design antitumor agents. We synthesised a series of hydrazones (compounds **4–24**) incorporating a 4-methylsulfonylbenzene scaffold and analysed their potential antitumor activity. Compounds **6**, **9**, **16**, and **20** had the most antitumor activity with a positive cytotoxic effect (PCE) of 52/59, 27/59, 59/59, and 59/59, respectively, while compounds **5**, **10**, **14**, **15**, **18**, and **19** had a moderate antitumor activity with a PCE of 11/59–14/59. Compound **20** was the most active and had a mean 50% cell growth inhibition (GI_50_) of 0.26 µM. Compounds **9** and **20** showed the highest inhibitory activity against COX-2, with a half-maximal inhibitory concentration (IC_50_) of 2.97 and 6.94 μM, respectively. Compounds **16** and **20** significantly inhibited EGFR (IC_50_ = 0.2 and 0.19 μM, respectively) and HER2 (IC_50_ = 0.13 and 0.07 μM, respectively). Molecular docking studies of derivatives **9**, **16**, and **20** into the binding sites of COX-2, EGFR, and HER2 were carried out to explore the interaction mode and the structural requirements for antitumor activity.

## Graphical Abstract

**Figure:**
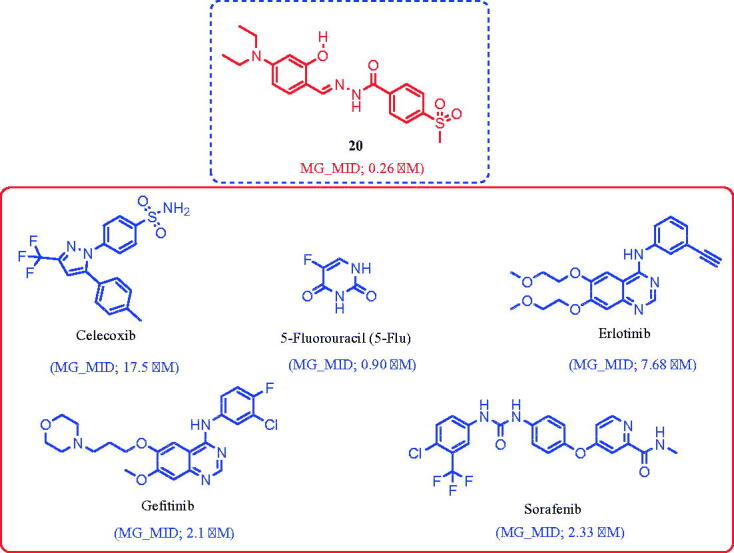

Compound 20 (MG_MID = 0.26 µM) is nearly 65-fold more potent than celecoxib (MG_MID = 17.5 µM), 3-fold more potent than 5-Fu (MG_MID = 0.90 µM), 30-fold more potent than erlotinib (MG_MID = 7.68 µM), and 9-fold more potent than gefitinib (MG_MID = 2.1 µM) and sorafenib (MG_MID = 2.33 µM).

## 1. Introduction

**Figure 1.**
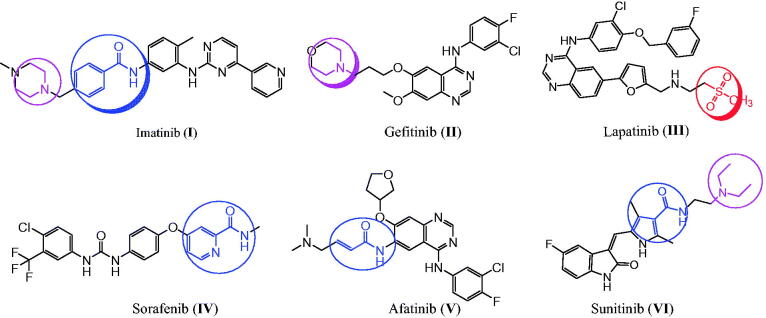
The reported anticancer agents with EGFR and HER2 inhibition activities.

**Figure 2.**
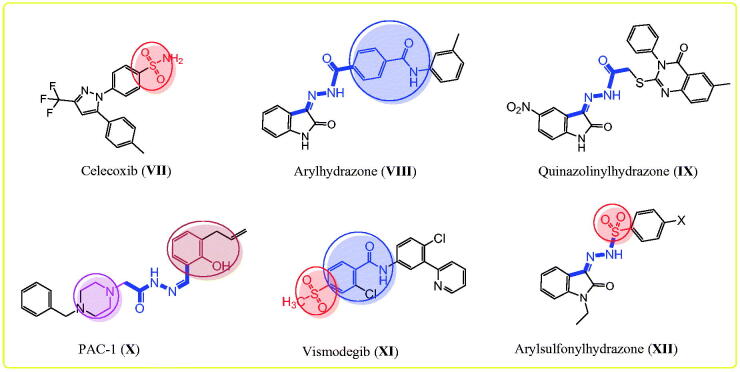
The reported anticancer agents bearing hydrazone, sulphonyl, and benzamide fragments.

Cancer is the most dangerous disease and a leading cause of death worldwide^1^. Also, cancer cells have evolved to become resistant to already used therapeutic agents^2–4^. Therefore, novel and effective antitumor agents are in high demand, and their development remains a challenge for pharmaceutical chemists^5–21^. The use of more than one drug in combination with cancer therapy has several side effects^22–24^. These effects can be diminished by using a single compound with multiple molecular mechanisms, which is currently the preferred therapeutic strategy^25–30^. EGFR and the structurally related human epidermal growth factor receptor 2 (HER2) are members of the tyrosine kinase receptor family^31–35^. EGFR and HER2 overexpression has been found in various cancers, such as prostate, breast, colon, and ovarian cancers, and their inhibition results in apoptotic-inducing activity in lung and breast cancers^36–39^. Therefore, EGFR and HER2 are important targets for antitumor agent design and development^27^^,^^32^^,^^33^^,^^39–42^. Several tyrosine kinase inhibitors, such as imatinib (**I**), gefitinib (**II**), lapatinib (**III**), sorafenib (**IV**), afatinib (**V**), and sunitinib (**VI**), are used to treat various cancers (Figure 1)^43–50^. In contrast, COX-2 isozyme is overexpressed in several cancers, such as colon, hepatocellular, gastric, breast, lung, prostate, and ovarian cancers, indicating that COX-2 is a target for cancer treatment^51^^,^^52^. These findings show that celecoxib (**VII**) and other selective COX-2 inhibitors can be used for cancer treatment and prevention (Figure 2)^53^^,^^54^. The anticancer mechanism through COX-2 inhibition might proceed *via* proliferation inhibition or apoptotic induction^55^.

Hugo Schiff first prepared Schiff’s bases or azomethines with an imine core skeleton (RHC = N-R) in 1864 through the reaction of carbonyl compounds and a primary amine^56^. The chemical and biological behaviours of Schiff’s bases are due to the lone pair of electrons on the imine core, which are responsible for their chelating properties^57^^,^^58^. Especially, hydrazone is an important, highly bioactive pharmacophore that can be used to design various antitumor agents, such as arylhydrazone (**VIII**), quinazolinylhydrazone (**IX**), and PAC-1 (**X**), and PAC-1 selectively induces apoptosis in cancer cells (Figure 2)^21^^,^^59–63^. Other compounds with hydrazones show the tumour-associated carbonic anhydrase IX and COX-2 inhibition activities^64–66^. Interestingly, compounds with methylsulfonylbenzene, such as vismodegib (**XI**), and hydrazine derivative–linked sulphonyl fragments, such as arylsulfonylhydrazone (**XII**), are potential antitumor agents against skin, hepatocellular, lung, and colon cancers and melanoma (Figure 2)^66–69^. The mechanism underlying the anticancer activity of a few hydrazones has been investigated through selective COX-2 inhibition, EGFR, and HER2 inhibition, in addition to apoptosis induction^39^^,^^60^^,^^66^^,^^69^.

**Figure 3.**
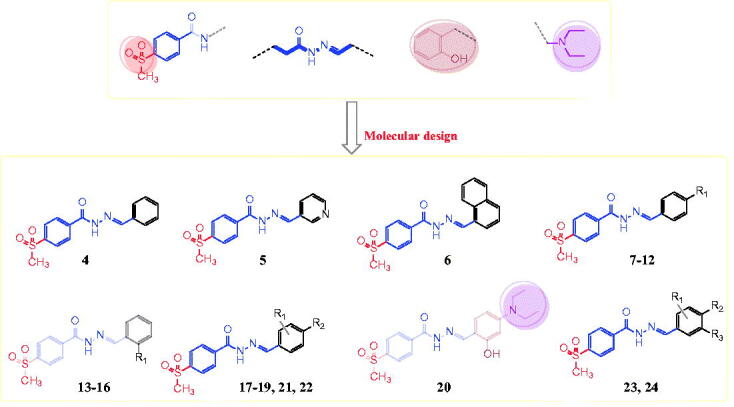
The designed target arylhydrazones **4–24** based on the chemical structure of compounds **I-XII**.

In this study, we synthesised a series of hydrazones (compounds **4–24**) incorporating a 4-methylsulfonylbenzene scaffold (Figure 3). The chemical structure of the designed compounds was based on some fragments of compounds shown in Figures 1 and 2. A 4-methylsulfonylbenzene core was linked with a hydrazone moiety and connected with various arylidenes with or without hydroxyl and *N,N*-diethylamine fragments (Figure 3). We also evaluated the *in vitro* antitumor activities of the synthesised compounds using 59 human cancer cell lines and investigated the structure–activity relationship (SAR) of the compounds with various substituents, depending on their antitumor activities. Next, we performed a cell cycle analysis and apoptotic induction assay of the most active compounds using the HL-60 cell line. We performed an enzymatic assay of the EGFR and HER2 inhibitory activity of the most promising compounds and evaluated the COX-2 inhibitory activity of the most active derivatives. Finally, we used molecular docking to predict the interaction mode of the biologically active compounds in the binding pockets of COX-2 isozyme, and EGFR, and HER2 tyrosine kinases.

## 2. Results and discussion

### 2.1. Chemistry

**Scheme 1.**
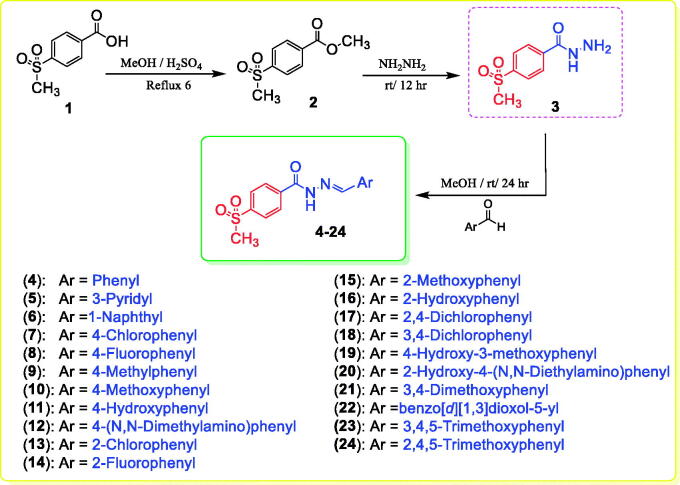
Synthesis of the designed hydrazones **4–24**.

4-(Methylsulfonyl)-*N*'-(substituted-benzylidene)benzohydrazides **4**–**24** were obtained with a yield of 85–95% by stirring an appropriate aldehyde and 4-(methylsulfonyl)benzohydrazide (**3**) in methanol containing a catalytic amount of acetic acid at room temperature (Scheme 1). Multiple spectral analyses were performed to confirm the structures of target compounds **4**–**24**. The amide fragment of the benzylidene benzohydrazide moiety (PhCONH=CHPh) was verified by ^1^H NMR spectra with peaks at 12.35–11.81 ppm for the amidic proton and by ^13 ^C NMR spectra with characteristic peaks at 163.0–161.3 ppm for the carbonyl group. In addition, the imine fragment of the benzylidene benzohydrazide moiety (PhCONH = CHPh) was verified by ^1^H NMR spectra with peaks at 9.12–8.36 ppm and by ^13 ^C NMR spectra with characteristic peaks at 151.4–143.9 ppm. The methyl group of the 4-methylsulfonyl moiety (SO_2_CH_3_) was verified by ^1^H NMR and ^13 ^C NMR spectra with peaks at 3.32–3.29 and 43.8–43.7 ppm, respectively.

### 2.2. Antitumor activity and SAR study

#### 2.2.1. Growth inhibition percentage (GI) at a single dose concentration of 10 µM

Compounds **4–24** were selected by the National Cancer Institute (Bethesda, MD, USA) for evaluation of their *in vitro* antitumor activity against a full panel of 59 cancer cell lines (Tables 1 and 2) taken from nine human tissue (i.e. blood, lung, colon, brain, skin, ovary, kidney, prostate, and breast)^17^^,^^40^^,^^70^. We performed the initial antitumor evaluation at a single dose concentration of 10 µM and calculated the GI in the 59 cell lines. The GI results were compared with those of imatinib, gefitinib, and 5-fluorouracil (5-FU) as reference drugs. Tables 1 and 2 summarise the GI activity of compounds **4–24**, which showed significant antitumor activity against the 59 cell lines at a 10 µM concentration with PCE (ratio between the number of cell lines with percentage growth inhibition from 10 to 100 and the total number of cell lines) of 5/59–59/59 and a percentage mean growth (MG) of 100.00%–20.59%. Compounds **5**, **6**, **9**, **10**, **14–16**, and **18–20** showed the highest PCE of 11/59–59/59 (MG = 99.74%–20.95%), while compounds **4**, **7**, **8, 11–13, 17,** and **21–24** showed the lowest PCE of ≤10/59 (MG = 100.00%–95.96%) compared to imatinib (PCE = 20/55 and MG = 92.62%). Interestingly, compounds **6**, **9**, **16**, and **20** were the most active antitumor agents (PCE = 52/59, 27/59, 59/59, and 59/59, respectively, and MG = 71.75%, 88.01%, 39.27%, and 20.59%, respectively), while compounds **5**, **14**, **15**, **18**, and **19** showed moderate activity (PCE = 13/59, 12/59, 14/59, 14/59, and 13/59, respectively, and MG = 98.37%, 95.16%, 95.92%, 94.23%, and 96.65%, respectively).

**Table 1.** Antitumor activity of the designed hydrazones at 10 *µ*M concentration.

Compd No	60 cell lines assay in one dose 10.0 µM concentration (GI%)^a^	
	PCE^b^	Most sensitive cell lines
**4**	10/59	***Leukaemia*** (HL-60 (TB): **60.3**, K-562: **11.3**, MOLT-4: **25.5**), ***NSC Lung Cancer*** (A549/ATCC: **28.5**, HOP-62: **12.0**, NCI-H522: **42.1**), ***CNS Cancer*** (U251: **20.4**), ***Melanoma*** (SK-MEL-2: **21.5**, UACC-257: **44.2**), ***Ovarian Cancer*** (OVCAR-8: **15.1**).
**5**	13/59	***Leukaemia*** (CCRF-CEM: **11.1**, HL-60(TB): **20.5**, K-562: **18.8**), ***NSC Lung Cancer*** (A549/ATCC: **10.6**, HOP-62: **16.5**, NCI-H226: **11.9**, NCI-H522: **15.1**), ***Colon Cancer*** (HT29: **13.3**), ***Ovarian Cancer*** (SK-OV-3: **16.9**), ***Renal Cancer*** (A498: **11.2**, UO-31: **16.3**), ***Prostate Cancer*** (PC-3: **17.9**), ***Breast Cancer*** (T-47D: **13.7**).
**6**	52/59	***Leukaemia*** (CCRF-CEM: **22.4**, HL-60(TB): **62.5**, K-562: **72.2**, MOLT-4: **53.2**, PRMI-8226: **12.3**, SR: **67.5**), ***NSC Lung Cancer*** (A549/ATCC: **58.7**, EKVX: **12.6**,HOP-62: **25.1**, NCI-H226: **16.3**, NCI-H23: **10.4**, NCI-H460: **33.2**, NCI-H522: **95.5**), ***Colon Cancer*** (COLO 205: **14.3**, HCC-2998: **15.5**, HCT-116: **38.2**, HCT-15: **50.2**, HT29: **50.5**, KM12: **49.2**, SW-620: **63.8**), ***CNS Cancer*** (SF-268: **14.3**, SF-295: **24.0**, SF-539: **10.7**, SNB-19: **20.2**, SNB-75: **10.3**, U251: **23.7**), ***Melanoma*** (LOX IMVI: **27.6**, MALME-3M: **30.3** , M14: **33.2**, MDA-MB-435: **82.9**, SK-MEL-2: **52.3**, SK-MEL-28: **10.6**, SK-MEL-5: **32.8**, UACC-257: **58.9**, UACC-62: **38.4**), ***Ovarian Cancer*** (IGROV1: **36.8**, OVCAR-3: **32.2**, OVCAR-8: **16.2**, NCI/ADR-RES: **26.5**, SK-OV-3: **21.9**), ***Renal Cancer*** (A498: **30.9**, CAKI-1: **32.8**, SN12C: **12.6**, TK-10: **14.9**, UO-31: **21.5**), ***Prostate Cancer*** (PC-3: **21.2**, DU-145: **11.2**), ***Breast Cancer*** (MCF7: **55.1**, HS 578 T: **14.9**, BT-549: **12.4**, T-47D: **14.7**, MDA-MB-468: **25.8**).
**7**	7/59	***Leukaemia*** (HL-60 (TB): **52.1**, K-562: **14.0**, MOLT-4: **29.4**), ***NSC Lung Cancer*** (NCI-H522: **15.7**), ***Melanoma*** (SK-MEL-2: **12.8**), ***Renal Cancer*** (A498: **12.3**), ***Prostate Cancer*** (PC-3: **22.8**).
**8**	9/59	***Leukaemia*** (CCRF-CEM: **11.8**, HL-60(TB): **23.1**, K-562: **13.6**, MOLT-4: **23.9**, SR: **20.2**), ***NSC Lung Cancer*** ( A549/ATCC: **11.1**, NCI-H522: **15.9**), ***Colon Cancer*** (HCT-116: **10.8**), ***Melanoma*** (UACC-257: **18.4**).
**9**	27/59	***Leukaemia*** (CCRF-CEM: **20.8**, HL-60(TB): **24.3**, K-562: **44.1**, MOLT-4: **22.6**, SR: **43.8**), ***NSC Lung Cancer*** (A549/ATCC: **12.7**, NCI-H522: **53.9**), ***Colon Cancer*** (HCT-116: **13.9**, HCT-15: **16.3**, HT29: **17.7**, KM12: **25.1**, SW-620: **15.2**), ***CNS Cancer*** (U251: **13.7**), ***Melanoma*** (MALME-3M: **15.2** , MDA-MB-435: **47.5**, SK-MEL-2: **19.8**, SK-MEL-5: **13.5**, UACC-257: **28.4**, UACC-62: **19.1**), ***Ovarian Cancer*** (IGROV1: **12.3**, NCI/ADR-RES: **14.6**), ***Renal Cancer*** (A498: **17.9**, CAKI-1: **25.0**, UO-31: **12.3**), ***Prostate Cancer*** (PC-3: **26.9**), ***Breast Cancer*** (MCF7: **21.4**, MDA-MB-468: **21.5**)
**10**	11/59	***Leukaemia*** (CCRF-CEM: **10.6**, HL-60(TB): **42.9**, MOLT-4: **21.1**), ***NSC Lung Cancer*** (A549/ATCC: **29.8** , NCI-H226: **13.4**, NCI-H522: **20.8**), ***Colon Cancer*** (HT29: **10.8**), ***Melanoma*** (SK-MEL-2: **11.8**, UACC-257: **29.0**), ***Ovarian Cancer*** (OVCAR-8: **11.3**), ***Prostate Cancer*** (PC-3: **13.8**).
**11**	5/59	***Leukaemia*** (HL-60(TB): **50.5**, K-562: **26.2**, MOLT-4: **32.5**), ***NSC Lung Cancer*** (NCI-H522: **11.7**), ***Prostate Cancer*** (PC-3: **10.9**).
**12**	9/59	***Leukaemia*** (HL-60(TB): **38.0**, K-562: **17.2**, MOLT-4: **29.8**), ***NSC Lung Cancer*** (A549/ATCC: **28.4**, HOP-62: **11.4**, NCI-H522: **40.4**), ***Colon Cancer*** (HT29: **20.5**), ***Melanoma*** (UACC-257: **39.2**), ***Renal Cancer*** (UO-31: **11.2**).
**13**	10/59	***Leukaemia*** (HL-60(TB): **47.7**, K-562: **32.5**, MOLT-4: **32.3**), ***NSC Lung Cancer*** (A549/ATCC: **21.6**, HOP-62: **12.7**), ***CNS Cancer*** (SNB-75: **15.1**), ***Melanoma*** ( UACC-257: **35.5**), ***Ovarian Cancer*** (SK-OV-3: **11.7**), ***Renal Cancer*** (UO-31: **15.7**), ***Breast Cancer*** (T-47D: **12.5**)
**14**	12/59	***Leukaemia*** (HL-60(TB): **36.0**, K-562: **25.7**, MOLT-4: **26.4**, SR: **49.2**), ***NSC Lung Cancer*** (A549/ATCC: **29.3**, HOP-62: **15.8**, NCI-H522: **26.0**), ***Colon Cancer*** (HT29: **18.6**), ***Melanoma*** ( UACC-257: **42.4**), ***Ovarian Cancer*** (OVCAR-8: **14.5**), ***Renal Cancer*** ( CAKI-1: **11.7**), ***Breast Cancer*** (T-47D: **11.8**).
**15**	14/59	***Leukaemia*** (CCRF-CEM: **16.1**, HL-60(TB): **36.5**, K-562: **23.4**, MOLT-4: **11.6**, SR: **15.6**), ***NSC Lung Cancer*** (A549/ATCC: **11.9**, HOP-62: **18.9**, NCI-H226: **10.3**), ***Colon Cancer*** (HCT-116: **10.7**), ***Melanoma*** (UACC-257: **10.1**), ***Ovarian Cancer*** (SK-OV-3: **17.1**), ***Renal Cancer*** (UO-31: **14.4**), ***Prostate Cancer*** (PC-3: **19.1**), ***Breast Cancer*** (T-47D: **18.2**)
**16**	59/59	***Leukaemia*** (CCRF-CEM: **84.7**, HL-60(TB): **93.7**, K-562: **59.5**, MOLT-4: **90.8**, PRMI-8226: **51.3**, SR: **79.6**), ***NSC Lung Cancer*** (A549/ATCC: **61.2**, EKVX: **74.2**,HOP-62: **71.8**, NCI-H226: **44.8**, NCI-H23: **45.2**, NCI-H322M: **61.6**, NCI-H460: **85.9**, NCI-H522: **84.9**), ***Colon Cancer*** (COLO 205: **68.8**, HCC-2998: **52.7**, HCT-116: **70.9**, HCT-15: **82.0**, HT29: **56.4**, KM12: **65.9**, SW-620: **56.7**), ***CNS Cancer*** (SF-268: **61.6**, SF-295: **62.0**, SF-539: **44.7**, SNB-19: **54.6**, SNB-75: **22.3**, U251: **72.2**), ***Melanoma*** (LOX IMVI: **78.6**, MALME-3M: **36.5** , M14: **67.5**, MDA-MB-435: **40.3**, SK-MEL-2: **60.0**, SK-MEL-28: **44.1**, SK-MEL-5: **67.8**, UACC-257: **77.4**, UACC-62: **83.6**), ***Ovarian Cancer*** (IGROV1: **59.0**, OVCAR-3: **77.2**, OVCAR-4: **56.3**, OVCAR-5: **35.3**, OVCAR-8: **72.1**, NCI/ADR-RES: **70.1**, SK-OV-3: **60.1**), ***Renal Cancer*** (786-0: **67.0**, A498: **24.8**, ACHN: **71.7**, CAKI-1: **65.1**, RXF 393: **47.0**, SN12C: **41.4**, TK-10: **54.2**, UO-31: **82.0**), ***Prostate Cancer*** (PC-3: **45.7**, DU-145: **59.6**), ***Breast Cancer*** (MCF7: **77.5**, MDA-MB-231/ATCC: **52.6**, HS 578 T: **18.5**, BT-549: **47.0**, T-47D: **47.6**, MDA-MB-468: **35.3**).
**17**	9/59	***NSC Lung Cancer*** (A549/ATCC: **13.7**, HOP-62: **10.2**, NCI-H226: **13.6**, NCI-H522: **25.0**), ***Colon Cancer*** (HT29: **11.3**), ***CNS Cancer*** (SNB-75: **10.9**), ***Melanoma*** (UACC-257: **29.4**), ***Renal Cancer*** (UO-31: **12.7**), ***Breast Cancer*** (MDA-MB-468: **18.1**).
**18**	14/59	***Leukaemia*** (HL-60(TB): **30.6**, K-562: **17.7**, MOLT-4: **31.7**, SR: **24.5**), ***NSC Lung Cancer*** (A549/ATCC: **20.1**, NCI-H522: **17.2**), ***Colon Cancer*** (HCT-116: **12.7**), ***Melanoma*** (MALME-3M: **12.2** , UACC-257: **21.8**, UACC-62: **11.2**), ***Ovarian Cancer*** (IGROV1: **15.0**), ***Renal Cancer*** (A498: **16.0**, CAKI-1: **10.1**), ***Prostate Cancer*** (PC-3: **10.3**).
**19**	13/59	***Leukaemia*** (HL-60(TB): **67.3**, K-562: **27.2**, MOLT-4: **30.9**, SR: **18.5**), ***NSC Lung Cancer*** (A549/ATCC: **29.3**, HOP-62: **12.1**, NCI-H522: **37.7**), ***Colon Cancer*** (HT29: **17.5**), ***CNS Cancer*** (U251: **13.0**), ***Melanoma*** (SK-MEL-2: **13.9**, UACC-257: **48.4**), ***Ovarian Cancer*** (OVCAR-8: **19.6**), ***Renal Cancer*** (UO-31: **12.8**).
**20**	59/59	***Leukaemia*** (CCRF-CEM: **93.1**, HL-60(TB): **100**, K-562: **87.6**, MOLT-4: **97.3**, PRMI-8226: **79.0**, SR: **91.0**), ***NSC Lung Cancer*** (A549/ATCC: **87.5**, EKVX: **84.5**, HOP-62: **87.1**, NCI-H226: **77.4**, NCI-H23: **67.2**, NCI-H322M: **75.0**, NCI-H460: **96.7**, NCI-H522: **90.1**), ***Colon Cancer*** (COLO 205: **91.4**, HCC-2998: **60.4**, HCT-116: **81.6**, HCT-15: **71.5**, HT29: **78.2**, KM12: **74.1**, SW-620: **67.0**), ***CNS Cancer*** (SF-268: **78.2**, SF-295: **78.6**, SF-539: **96.0**, SNB-19: **68.0**, SNB-75: **44.8**, U251: **79.4**), ***Melanoma*** (LOX IMVI: **90.3**, MALME-3M: **86.0** , M14: **96.6**, MDA-MB-435: **62.8**, SK-MEL-2: **80.5**, SK-MEL-28: **72.88**, SK-MEL-5: **100**, UACC-257: **100**, UACC-62: **>100**), ***Ovarian Cancer*** (IGROV1: **78.8**, OVCAR-3: **66.6**, OVCAR-4: **65.3**, OVCAR-5: **67.5**, OVCAR-8: **83.2**, NCI/ADR-RES: **82.2**, SK-OV-3: **61.0**), ***Renal Cancer*** (786-0: **84.6**, A498: **54.7**, ACHN: **82.9**, CAKI-1: **79.6**, RXF 393: **71.5**, SN12C: **68.8**, TK-10: **69.5**, UO-31: **100**), ***Prostate Cancer*** (PC-3: **50.8**, DU-145: **75.5**), ***Breast Cancer*** (MCF7: **91.2**, MDA-MB-231/ATCC: **56.8**, HS 578 T: **32.0**, BT-549: **69.3**, T-47D: **70.0**, MDA-MB-468: **98.8**).
**21**	8/59	***Leukaemia*** (HL-60(TB): **45.9**, K-562: **19.1**, MOLT-4: **34.1**), ***NSC Lung Cancer*** (NCI-H522: **15.7**), ***Colon Cancer*** (HCT-116: **11.0**), ***CNS Cancer*** (SNB-75: **14.2**), ***Renal Cancer*** (CAKI-1: **11.0**), ***Prostate Cancer*** (PC-3: **11.5**).
**22**	9/59	***Leukaemia*** (CCRF-CEM: **13.0**, HL-60(TB): **43.0**, K-562: **14.5**, MOLT-4: **29.9**), ***NSC Lung Cancer*** (A549/ATCC: **32.3**, NCI-H522: **14.0**), ***Colon Cancer*** (HT29: **11.0**), ***Melanoma*** (SK-MEL-2: **10.9**, UACC-257: **40.9**).
**23**	9/59	***Leukaemia*** (HL-60(TB): **44.2**, K-562: **37.6**, MOLT-4: **32.6**), ***NSC Lung Cancer*** (A549/ATCC: **22.8**), ***Colon Cancer*** (COLO 205: **14.6**), ***CNS Cancer*** (SNB-75: **11.3**), ***Melanoma*** (UACC-257: **28.6**), ***Ovarian Cancer*** (SK-OV-3: **14.2**), ***Renal Cancer*** (UO-31: **10.2**).
**24**	8/59	***Leukaemia*** (MOLT-4: **21.4**), ***NSC Lung Cancer*** (A549/ATCC: **17.1**, NCI-H522: **39.8**), ***Colon Cancer*** (HT29: **17.9**), ***Melanoma*** (K-MEL-2: **11.7**, UACC-257: **21.6**), ***Renal Cancer*** (UO-31: **10.5**), ***Prostate Cancer*** (PC-3: **18.6**).
**Imatinib**	20/54	***Leukaemia*** (MOLT-4: **18.0**, PRMI-8226: **12.6**, SR: **14.6**), ***NSC Lung Cancer*** (EKVX: **15.7**, NCI-H226: **10.6**, NCI-H23: **17.1**), ***Colon Cancer*** (HCT-116: **18.6**, HCT-15: **11.5**, HT29: **47.1**), ***CNS Cancer*** (SF-295: **15.1**, SF-539: **24.5**, U251: **10.6**), ***Melanoma*** (LOX IMVI: **11.6**, SK-MEL-5: **22.3**), ***Renal Cancer*** (A498: **13.7**), ***Prostate Cancer*** (PC-3: **10.6**, DU-145: **14.4**), ***Breast Cancer*** (MDA-MB-231/ATCC: **11.2**, T-47D: **18.6**, MDA-MB-468: **29.1**).

^a^Growth Inhibition percentages (GI%) lower than 10% are not shown.

^b^PCE: Positive cytotoxic effect (ratio between the number of cell lines with percentage growth inhibition from 10 to 100 and the total number of cell lines).

**Table 2.** Growth inhibition percentage *(GI%)* of the most active hydrazones against individual cell lines.

Growth inhibition percentage (GI%)								
Subpanel tumour cell lines		6	9	16	20	Imatinib	Gefitinib	5-Fu
Leukaemia								
CCRF-CEM	22.4		20.8	84.7	93.1	–	96.0	42.9
HL-60(TB)	62.5		24.3	93.7	>100	–	100	52.1
K-562	72.2		44.1	59.5	87.6	NT	NT	57.7
MOLT-4	53.2		22.6	90.8	97.3	18.0	>100	56.9
PRMI-8226	12.3		–	51.3	79.0	12.6	18.0	58.6
SR	67.5		43.8	79.6	91.0	14.6	44.7	75.2
Non-Small Cell Lung Cancer								
A549/ATCC	58.7		12.7	61.2	87.5	–	87.0	65.8
EKVX	12.6		–	74.2	84.5	15.7	92.3	41.6
HOP-62	25.1		–	71.8	87.1	NT	NT	52.2
NCI-H226	16.3		–	44.8	77.4	10.6	25.2	30.5
NCI-H23	10.4		–	45.2	67.2	17.1	86.2	61.0
NCI-H322M	–		–	61.6	75.0	NT	NT	40.5
NCI-H460	33.2		–	85.9	96.7	–	42.7	87.0
NCI-H522	95.5		53.9	84.9	90.1	NT	NT	42.0
Colon Cancer								
COLO 205	14.3		–	68.8	91.4	–	49.6	59.8
HCC-2998	15.5		–	52.7	60.4	–	55.3	>100
HCT-116	38.2		13.9	70.9	81.6	18.6	72.1	82.2
HCT-15	50.2		16.3	82.0	71.5	11.5	71.5	73.5
HT29	50.5		17.7	56.4	78.2	47.1	50.3	72.9
KM12	49.2		25.1	65.9	74.1	–	36.9	59.3
SW-620	63.8		15.2	56.7	67.0	–	29..3	49.9
CNS Cancer								
SF-268	14.3		–	61.6	78.2	–	64.1	41.0
SF-295	24.0		–	62.0	78.6	15.1	15.8	30.9
SF-539	10.7		–	44.7	96.0	24.5	15.1	>100
SNB-19	20.2		–	54.6	68.0	–	73.8	34.1
SNB-75	10.3		–	22.3	44.8	–	61.5	34.1
U251	23.7		13.7	72.2	79.4	10.6	56.5	49.7
Melanoma								
LOX IMVI	27.6		–	78.6	90.3	11.6	46.0	69.6
MALME-3M	30.3		15.2	36.5	86.0	–	22.1	41.8
M14	33.2		–	67.5	96.6	–	89.7	NT
MDA-MB-435	82.9		47.5	40.3	62.8	–	63.3	63.4
SK-MEL-2	52.3		19.8	60.0	80.5	NT	NT	–
SK-MEL-28	10.6		–	44.1	72.8	–	27.3	NT
SK-MEL-5	32.8		13.5	67.8	>100	22.3	58.1	66.3
UACC-257	58.9		28.4	77.4	>100	–	24.7	80.5
UACC-62	38.4		19.1	83.6	>100	–	28.3	60.3
Ovarian Cancer								
IGROV1	36.8		12.3	59.0	78.8	–	57.2	48.8
OVCAR-3	32.2		–	77.2	66.6	–	55.8	52.6
OVCAR-4	–		–	56.3	65.3	–	11.5	40.6
OVCAR-5	–		–	35.3	67.5	–	54.5	55.7
OVCAR-8	16.2		–	72.1	83.2	–	78.2	NT
NCI/ADR-RES	26.5		14.6	70.1	82.2	–	86.3	52.4
SK-OV-3	21.9		–	60.1	61.0	–	83.0	22.5
Renal Cancer								
786-0	–		–	67.0	84.6	–	80.7	51.3
A498	30.9		17.9	24.8	54.7	13.7	65.9	>100
ACHN	–		–	71.7	82.9	–	91.5	60.7
CAKI-1	32.8		25.0	65.1	79.6	–	98.8	60.6
RXF 393	–		–	47.0	71.5	–	36.0	65.7
SN12C	12.6		–	41.4	68.8	–	85.5	46.0
TK-10	14.9		–	54.2	69.5	–	75.0	33.1
UO-31	21.5		12.3	82.0	>100	–	89.7	58.7
Prostate Cancer								
PC-3	21.2		26.9	45.7	50.8	10.6	49.6	41.8
DU-145	11.2		–	59.6	75.5	14.4	69.6	64.5
Breast Cancer								
MCF7	55.1		21.4	77.5	91.2	–	56.0	88.5
MDA-MB-231/ATCC	–		–	52.6	56.8	11.2	31.4	21.9
HS 578 T	14.9		–	18.5	32.0	–	10.0	>100
BT-549	12.4		–	47.0	69.3	–	61.2	62.2
T-47D	14.7		–	47.6	70.0	18.6	26.8	43.3
MDA-MB-468	25.8		21.5	35.3	98.8	29.1	>100	NT

NT: Not tested.

Structure correlation analysis gave the following results:

- 2-hydroxyphenyl derivatives, such as compounds **16** and **20** (PCE = 59/59), had significant and potent antitumor activity compared to unsubstituted phenyl and 4-hydroxyphenyl derivatives, such as compounds **4**, **11**, and **19** (PCE = 10/59, 5/59, and 13/59, respectively).
- Derivatives incorporating the 2-hydroxyphenyl moiety, such as compounds **16** and **20** (PCE = 59/59), were potent antitumor agents compared to the corresponding 2-substituted compounds **13–15** (PCE = 10/59–14/59).
- Derivatives based on a naphthalene scaffold, such as compound **6**, showed a sharp increase in antitumor activity (PCE = 52/59) compared to phenyl and pyridyl compounds **4** and **5** (PCE = 10/59 and 13/59, respectively).
- Replacement of the phenyl moiety of compound **4** with a 4-tolyl fragment, such as in compound **9**, increased antitumor activity (PCE = 10/59 and 27/59, respectively).
- The 4-tolyl compound **9** showed significant antitumor activity (PCE = 27/59) compared to the corresponding halogenated derivatives, such as compounds **7** and **8** (PCE = 7/59 and 9/59, respectively), and derivatives incorporating 4-methoxyphenyl (compound **10**), 4-hydroxyphenyl (compound **11**), and 4-(*N,N*-dimethylamino)phenyl (compound **12**) fragments (PCE = 11/59, 5/59, and 9/59 respectively).
- Introduction of one or more methoxy group at the phenyl fragment, such as in compounds **10** and **21–24**, did not improve antitumor activity (PCE = 11/59, 8/59, 9/59, 9/59, and 8/59, respectively) compared to the unsubstituted phenyl compound **4** (PCE = 10/59).
- Insertion of a methoxy group into compound **11** (PCE = 5/59) at position three significantly increased the antitumor activity of compound **19** (PCE = 13/59).
- Insertion of a halogen atom into a phenyl derivative, such as compound **4** (PCE = 10/59), produced compounds **7**, **8**, **13**, and **14** with retention of antitumor activity (PCE = 7/59, 9/59, 10/59, and 12/59, respectively).
- Replacement of the 3,4-dimethoxyphenyl moiety, such as compound **21**, with a 3,4-dichlorophenyl derivative, such as compound **18**, increased antitumor activity (PCE = 8/59 and 14/59, respectively).

The broad-spectrum and selectivity of compounds **4–24** (Tables 1 and 2) against the 59 cell lines showed that compounds **6**, **9**, **16**, and **20** had significant GI (>10–100%) against most of the cancer cell lines tested (leukaemia, non–small cell lung cancer [NSCLC], melanoma, and colon, CNS, ovarian, renal, prostate, and breast cancer) compared to imatinib (GI < 10%–47.1%). Compounds **6**, **9**, **16**, and **20** showed significant antitumor activity against leukaemia (GI = 12.3–100%), NSCLC (GI = 10.4%–96.7%), colon cancer (GI = 13.9%–91.4%), CNS cancer (GI = 10.3%–96.0%), melanoma (GI = 10.6–100%), ovarian cancer (GI = 12.3%–83.2%), renal cancer (GI = 12.3–100%), prostate cancer (GI = 11.2%–75.5%), and breast cancer (GI = 12.4%–98.8%). In contrast, the antitumor activity of imatinib was moderate against leukaemia (GI = 12.6%–18.0%), NSCLC (GI = 10.6%–17.1%), colon cancer (GI = 11.5%–47.1%), CNS cancer (GI = 10.6%–24.5%), melanoma (GI = 11.6%–22.3%), ovarian cancer (GI < 10.0%), renal cancer (GI < 10.0%–13.7%), prostate cancer (GI = 10.6%–14.4%), and breast cancer (GI = 11.2%–29.1%).

#### 2.2.2. Gi50, TGI, and LC_50_ of compound 20 and a comparative study

Compound **20** was the most active broad-spectrum antitumor agent among the 21 compounds tested, so we evaluated its potencies (50% cell growth inhibition [GI_50_]) in an advanced assay against a panel of 60 cancer cell lines (Figure S1 and Tables 3 and 4) at tenfold dilution of five different concentrations (100, 10, 1, 0.1, and 0.01 µM). We also comparatively compared the assay results to the potencies of celecoxib, erlotinib, gefitinib, sorafenib, vismodegib, and 5-Flu as reference drugs (Tables 3 and 4). Accordingly, we listed three dose–response parameters of antitumor activity for compound **20** and the reference drugs against each cell line in Table 4, including GI_50_, total cell growth inhibition (TGI), and median lethal concentration (LC_50_). In addition, we calculated the mean GI_50_ graph midpoints (GI_50_ MG_MID) for these parameters in order to obtain an average activity parameter over all cell lines for each molecule. Tables 3 and 4 list the GI_50_ values, which show that compound **20** had significantly potent antitumor activity, with GI_50_ = 0.063–11.7 µM. We compared this value to celecoxib, erlotinib, gefitinib, sorafenib, and vismodegib (GI_50_ = 3.98–63.09, 0.10–100.0, 0.0125–10.0, 1.26–3.98, and 19.95–100.0 µM, respectively (Table 4). With regard to individual human organs, compound **20** showed significantly potent antitumor activity against leukaemia (mean GI_50_ = 0.23 µM), NSCLC (mean GI_50_ = 0.26 µM), colon cancer (mean GI_50_ = 0.27 µM), CNS cancer (mean GI_50_ = 0.22 µM), melanoma (mean GI_50_ = 0.31 µM), ovarian cancer (mean GI_50_ = 1.10 µM), renal cancer (mean GI_50_ = 1.40 µM), prostate cancer (mean GI_50_ = 0.23 µM), and breast cancer (mean GI_50_ = 2.20 µM) (Tables 3 and 4). In contrast, celecoxib, 5-Fu, erlotinib, gefitinib, and sorafenib had the following mean GI_50_: leukaemia (12.35, 2.82, 27.85, 2.56, and 1.91 µM, respectively), NSCLC (25.09, 13.59, 13.11, 2.05, and 2.34 µM, respectively), colon cancer (20.73, 0.27, 51.68, 5.23, and 2.19 µM, respectively), CNS cancer (17.22, 14.38, 16.99, 5.64, and 2.33 µM, respectively), melanoma (22.46, 7.83, 23.74, 3.68, and 1.87 µM, respectively), ovarian cancer (17.14, 5.17, 5.52, 3.05, and 2.89 µM, respectively), renal cancer (19.87, 0.93, 2.46, 1.41, and 2.86 µM, respectively), prostate cancer (14.22, 1.46, 20.90, 3.29, and 2.58 µM, respectively), and breast cancer (17.49, 6.87, 24.72, 4.67, and 2.17 µM, respectively) (Table 4). Comparing the antitumor activity of compound **20** with the reference drugs, we found that compound **20** (GI_50_ MG_MID = 0.26 µM) is nearly 65-fold more potent than celecoxib (GI_50_ MG_MID =17.5 µM), 3-fold more potent than 5-Fu (GI_50_ MG_MID = 0.90 µM), 30-fold more potent than erlotinib (GI_50_ MG_MID = 7.68 µM), and 9-fold more potent than gefitinib (GI_50_ MG_MID = 2.1 µM) and sorafenib (GI_50_ MG_MID = 2.33 µM).

**Table 3.** Influence of compound **20**, and reference drugs on the growth of the individual tumour cell lines; median growth inhibitory (GI_50_, *µ*M).

*Subpanel tumour cell lines*	GI_50_ (*µ*M)					
	**20** (799156/1)^a^	**Celecoxib** (719627)^a^	**Erlotinib** (718781)^a^	**Gefitinib** (759856)^a^	**Sorafenib** (747971)^a^	**Vismodegib** (755986)^a^
*Leukaemia*						
CCRF-CEM	0.078	12.58	50.12	0.398	1.99	31.62
HL-60(TB)	0.164	15.85	25.12	1.0	1.58	31.62
K-562	0.604	10.00	31.62	2.51	3.16	31.62
MOLT-4	0.164	15.85	25.12	1.995	3.16	31.62
PRMI-8226	0.297	3.98	25.12	6.31	1.58	31.62
SR	0.063	15.85	10.00	3.16	3.16	39.81
*Non-Small Cell Lung Cancer*						
A549/ATCC	0.402	15.85	10.00	2.51	3.16	39.81
EKVX	0.162	19.95	0.199	0.0501	2.51	39.81
HOP-62	0.085	63.09	15.85	3.16	1.99	79.43
HOP-92	0.491	NT^b^	3.98	0.316	1.58	25.12
NCI-H226	0.073	NT^b^	39.81	3.98	1.99	50.12
NCI-H23	0.446	15.85	31.62	2.51	1.99	50.12
NCI-H322M	0.228	19.95	0.10	0.063	2.51	100
NCI-H460	0.113	15.85	6.31	3.16	2.51	39.81
NCI-H522	0.395	NT^b^	1.00	1.0	1.99	25.12
*Colon Cancer*						
COLO 205	0.269	15.85	50.12	7.94	1.99	39.81
HCC-2998	0.301	15.85	79.43	2.51	3.16	39.81
HCT-116	0.121	50.01	6.31	3.16	1.58	50.12
HCT-15	0.092	15.85	3.98	3.98	2.51	31.62
HT29	0.297	15.85	63.09	3.98	1.99	25.12
KM12	0.404	15.85	79.43	10.0	1.58	31.62
SW-620	0.387	15.85	79.43	5.011	2.51	63.09
*CNS Cancer*						
SF-268	0.0789	15.85	15.84	2.51	2.51	39.81
SF-295	0.200	15.85	15.84	10.0	1.58	31.62
SF-539	0.244	19.95	19.95	10.0	1.58	50.12
SNB-19	0.428	19.95	12.58	3.16	3.16	63.09
SNB-75	0.228	15.85	12.58	5.011	3.16	25.12
U251	0.137	15.85	25.12	3.16	1.99	50.12
*Melanoma*						
LOX IMVI	0.0997	19.95	6.31	3.16	1.58	39.81
MALME-3M	0.44	19.95	1.58	3.98	1.99	39.81
M14	0.171	63.09	6.31	1.995	1.99	31.62
MDA-MB-435	0.623	15.85	19.95	3.16	1.58	31.62
SK-MEL-2	0.307	15.85	6.31	10.0	1.99	39.81
SK-MEL-28	0.340	15.85	50.12	3.16	2.51	39.81
SK-MEL-5	0.223	15.85	19.5	3.16	1.58	25.12
UACC-257	0.340	19.95	100	2.51	1.99	50.12
UACC-62	0.234	15.85	3.16	1.995	1.58	31.62
*Ovarian Cancer*						
IGROV1	0.196	15.85	0.316	3.16	2.51	100
OVCAR-3	0.125	15.85	3.98	5.011	3.16	50.12
OVCAR-4	0.063	12.58	10.00	6.31	3.16	39.81
OVCAR-5	6.48	19.95	7.94	3.98	3.16	100
OVCAR-8	0.25	19.95	7.94	0.631	3.16	31.62
NCI/ADR-RES	0.324	15.85	7.94	1.26	2.51	31.62
SK-OV-3	0.251	19.95	0.50	1.0	2.51	NT^b^
*Renal Cancer*						
786-0	0.182	39.81	10.00	3.16	3.16	NT^b^
A498	9.81	15.85	2.51	0.631	2.51	31.62
ACHN	0.117	15.85	0.199	0.158	2.51	39.81
CAKI-1	0.104	19.95	0.199	0.5012	3.16	79.43
RXF 393	0.271	15.85	3.98	3.16	2.51	25.12
SN12C	0.354	15.85	1.58	1.258	2.51	39.81
TK-10	0..207	19.95	0.251	0.794	3.98	63.09
UO-31	0.139	15.85	1.00	1.584	2.51	31.62
*Prostate Cancer*						
PC-3	0.319	12.58	39.81	5.011	1.99	25.12
DU-145	0.144	15.85	1.99	1.584	3.16	50.12
*Breast Cancer*						
MCF7	0.253	15.85	100.00	5.011	2.51	31.62
MDA-MB-231/ATCC	0.308	15.85	6.31	3.98	1.26	19.95
HS 578 T	11.7	19.95	6.31	7.94	2.51	79.43
BT-549	0.346	19.95	31.62	3.16	3.16	NT^b^
T-47D	0.39	15.85	3.98	7.94	1.58	31.62
MDA-MB-468	0.203	NT^b^	0.126	0.01258	1.99	19.95

^a^https://dtp.cancer.gov/dtpstandard/dwindex/index.jsp.

^b^NT: Not tested.

**Table 4.** Average antitumor activity of compound **20**, and reference drugs against tumour cell lines from nine different organs at 10-fold dilution of five concentrations; median growth inhibitory (GI_50_, *µ*M), total growth inhibitory (TGI, *µ*M) and median lethal (LC_50_, *µ*M)^a^.

	Subpanel tumour cell lines										MG-MID^b^
Compd. No. (NSC)^c^	Activity	leukaemia	NSC lung cancer	colon cancer	CNS cancer	melanoma	ovarian cancer	renal cancer	prostate cancer	breast cancer	
**20** (799156)	GI_50_	**0.23**	**0.26**	**0.27**	**0.22**	**0.31**	**1.10**	**1.40**	**0.23**	**2.20**	**0.26**
	TGI	83.5	65.0	c	71.7	45.6	70.0	80.3	c	69.5	46.8
	LC_50_	c	c	c	c	c	c	c	c	c	c
**Celecoxib** (719627)	GI_50_	**12.35**	**25.09**	**20.73**	**17.22**	**22.46**	**17.14**	**19.87**	**14.22**	**17.49**	**17.5**
	TGI	29.27	41.93	41.39	31.62	38.49	34.20	41.19	28.37	34.89	34.0
	LC_50_	67.09	67.08	66.51	58.76	64.31	65.91	61.22	57.01	67/03	63.3
**5-Flu** (19893)	GI_50_	**2.82**	**13.59**	**0.27**	**14.38**	**7.83**	**5.17**	**0.93**	**1.46**	**6.87**	**0.90**
	TGI	68.37	80.45	51.41	73.70	61.57	38.36	46.25	c	68.01	43.4
	LC_50_	c	c	89.30	c	95.42	92.87	c	c	c	95.6
**Erlotinib** (718781)	GI_50_	**27.85**	**13.11**	**51.68**	**16.99**	**23.74**	**5.52**	**2.46**	**20.90**	**24.72**	**7.68**
	TGI	96.57	73.76	c	82.11	77.89	74.41	42.59	c	70.53	66.3
	LC_50_	c	97.71	c	c	93.31	97.06	89.15	c	96.43	95.6
**Gefitinib** (759856)	GI_50_	**2.56**	**2.05**	**5.23**	**5.64**	**3.68**	**3.05**	**1.41**	**3.29**	**4.67**	**2.1**
	TGI	12.07	13.86	18.47	19.62	12.49	33.29	12.50	31.62	18.62	14.3
	LC_50_	93.85	94.68	51.74	50.56	36.40	83.58	52.82	89.72	52.47	51.9
**Sorafenib** (747971)	GI_50_	**1.91**	**2.34**	**2.19**	**2.33**	**1.87**	**2.89**	**2.86**	**2.58**	**2.17**	**2.33**
	TGI	42.12	7.99	7.94	7.64	6.29	14.43	10.86	10.27	9.35	9.11
	LC_50_	c	49.15	38.06	27.77	28.57	71.34	54.75	69.91	57.29	43.1

^a^GI_50_, molar concentration of the compound that inhibits 50% net cell growth; TGI, molar concentration of the compound leading to total inhibition; and LC_50_, molar concentration of the compounds leading to 50% net cell death.

^b^Full panel mean-graph midpoint (*µ*M).

^c^https://dtp.cancer.gov/dtpstandard/dwindex/index.jsp.

c: Compounds showed values ≥ 100 *µ*M. Bold values used only for more precise comparison.

### 2.3. Apoptosis assay

#### 2.3.1. Annexin V–FITC apoptosis assay

**Figure 4.**
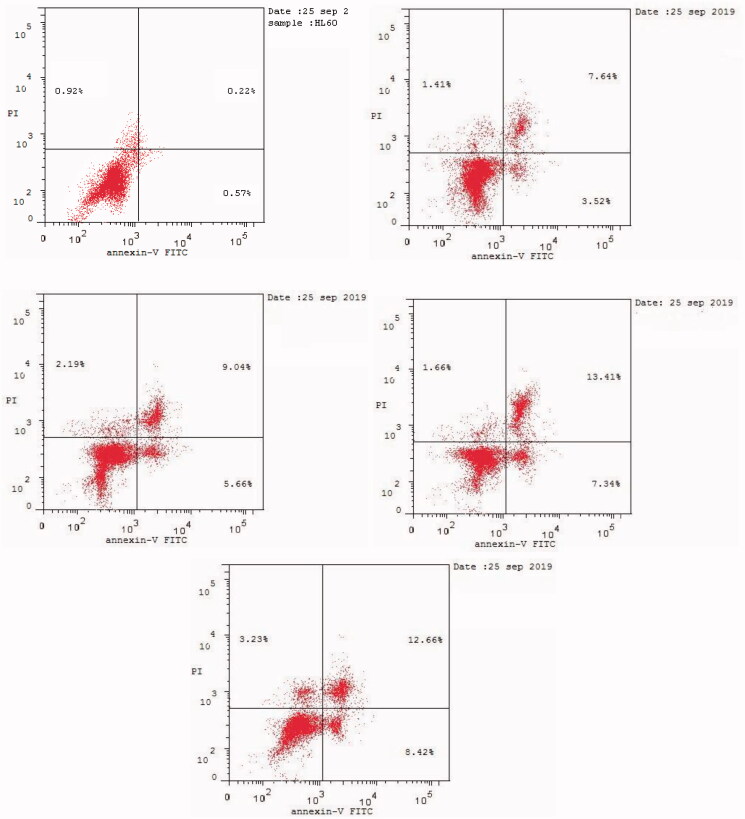
Effect of DMSO (upper left panel), and compounds **6** (upper right panel), **9** (middle left panel), **16** (middle right panel), and **20** (lower panel) on the percentage of annexin V-FITC-positive staining in HL60 cells.).

Apoptosis induction is the most important mechanism by which major chemotherapeutics kill cancer cells^71^. Apoptosis causes cellular changes whereby the translocation of phosphatidylserine (PS) occurs through the plasma membrane from the inside to the outside^71^. Annexin-V can bind to PS, which can be used as a sensitive probe for PS on the outer side of the plasma membrane^72^. We performed cytometric analysis to distinguish apoptosis from the necrosis mode of HL60 cell death induced by the most active compounds **6**, **9**, **16**, and **20** using annexin V–fluorescein isothiocyanate (FITC)/propidium iodide (AV/PI) dual-staining assay with the BD FACSCalibur (BD Biosciences, San Jose, CA, USA) (Table 5). The AV/PI staining of HL60 cells was performed at a mixed molar concentration of 10 µM with compounds **6**, **9**, **16**, and **20** for 24 h. Figure 4 and Table 5 show the results of treating HL60 cells with compounds **6**, **9**, **16**, and **20** for 24 h. We found an increase in the early apoptosis ratio (Figure 4, lower-right quadrant of the cytogram) from 0.57% in the control sample (DMSO) to 3.52%–8.42% and a sharp increase in the late apoptosis ratio (Figure 4, upper-right quadrant of the cytogram) from 0.22% to 7.64%–13.41%. These data support the apoptotic mechanism underlying programmed cell death induced by compounds **6**, **9**, **16**, and **20** rather than the necrotic pathway.

**Table 5.** Effect of compounds **6**, **9**, **16**, and **20** and DMSO on the percentage of HL60 cells stained positive for annexin V-FITC

		Apoptosis		
Sample/cell line	Total	Early	Late	Necrosis
**6**	12.57	3.52	7.64	1.41
**9**	16.89	5.66	9.04	2.19
**16**	22.41	7.34	13.41	1.66
**20**	24.31	8.42	12.66	3.23
DMSO	1.71	0.57	0.22	0.92

#### 2.3.2. *In vitro* cell cycle analysis

**Figure 5.**
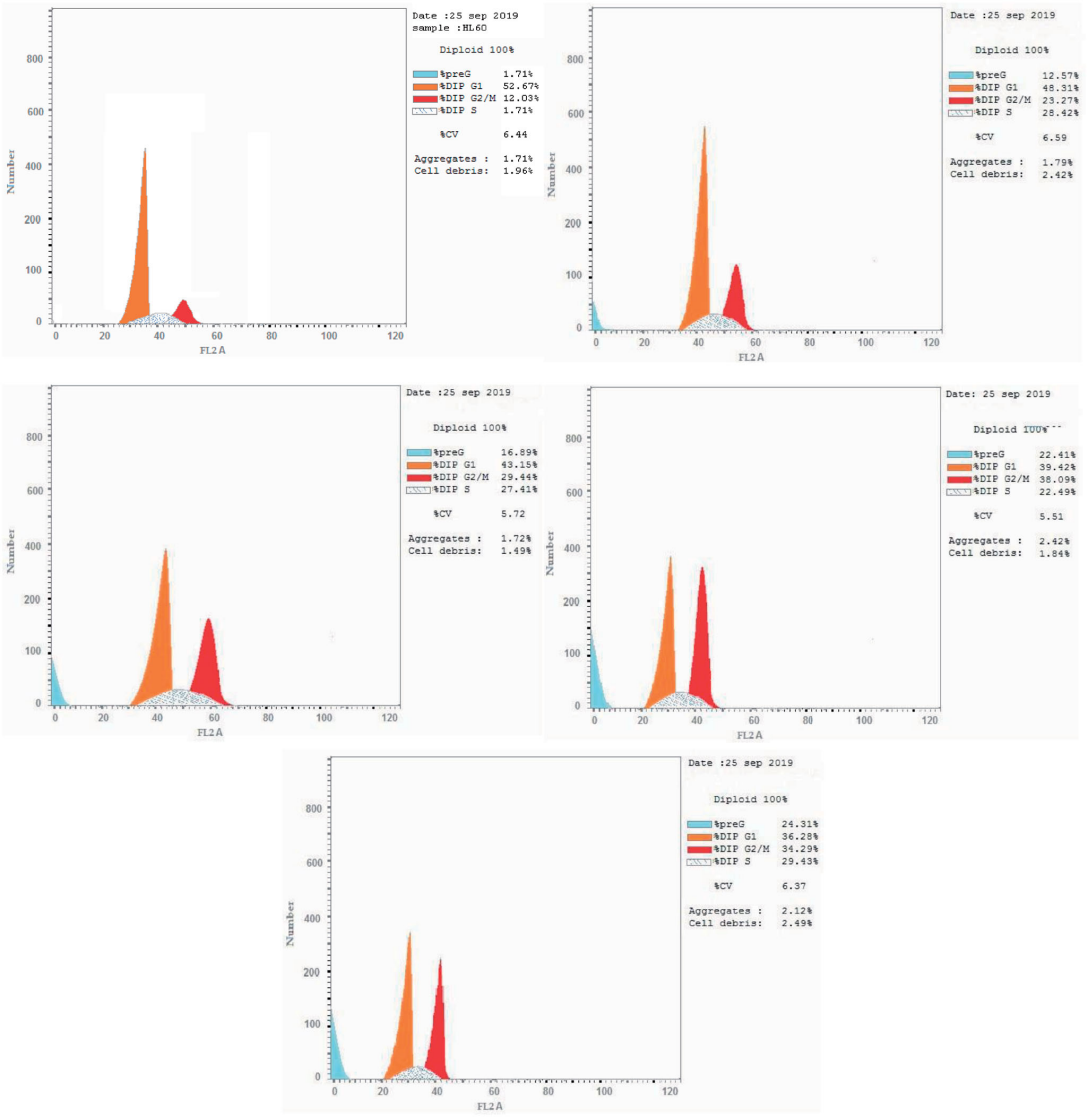
Cell cycle analysis of HL60 cells treated with DMSO (upper left panel) and compounds **6** (upper right panel), **9** (middle left panel), **16** (middle right panel), and **20** (lower panel).

Antitumor agents can induce apoptosis by activating signalling pathways, leading to G2/M phase arrest^73^^,^^74^. Flow cytometry is used to measure cell growth in different cell cycle phases (pre-G1, G1, S, and G2/M)^73^^,^^74^. We selected the most active compounds **6**, **9**, **16**, and **20** for further analysis of their effects on cell cycle progression in the HL60 cell line (Figure 5 and Table 6). We used the solvent DMSO as a negative control. Briefly, we incubated HL60 cells with 10 µM compounds **6**, **9**, **16**, and **20** for 24 h. Compounds **6**, **9**, **16**, and **20** interfered with the normal cell cycle of HL60 cells. There was a significant effect on the percentage of apoptotic cells, as indicated by an increase in cells in the pre-G1 phase (12.57%–24.31%) and the G2/M phase (23.27%–38.09%) compared to the control (1.71% and 12.03% cells, respectively). In contrast, the percentage of cells in S and G0/G1 phases significantly decreased (22.49%–29.43% and 36.28%–48.31%, respectively) compared to the control (35.3% and 52.67%, respectively), causing cell cycle arrest. These results clearly indicated that compounds **6**, **9**, **16**, and **20** arrests the G2/M phase of the cell cycle (Figure 5 and Table 6).

**Table 6.** Effect of compounds **6**, **9**, **16**, and **20** and DMSO on the cell cycle of HL60 cells.

Sample	**Conc.** **(µM)**	%G0-G1	%S	%G2-M	%Pre-G1
**6**	10.0	48.31	28.42	23.27	12.57
**9**	10.0	43.15	27.41	29.44	16.89
**16**	10.0	39.42	22.49	38.09	22.41
**20**	10.0	36.28	29.43	34.29	24.31
DMSO	0.0	52.67	35.3	12.03	1.71

### 2.4. Enzymatic inhibition assay

#### 2.4.1. Cox-2 inhibition activity

COX-2 is overexpressed in several cancer cell lines during cell proliferation. Its inhibition is used as a target for cancer treatment and prevention^51^^,^^52^^,^^75^. Therefore, we performed COX-2 inhibition assays (kit catalogue no. 560101; Cayman Chemicals Inc., Ann Arbour, MI, USA) using compounds **6**, **9**, **16**, and **20**, which showed the highest antitumor activity, in addition to the reference drug celecoxib^15^^,^^76^. The results were expressed as IC_50_ (µM) as the mean of three acquired determinations (Table 7). The IC_50_ of celecoxib as a COX-2 inhibitor was 2.79 µM. Compounds **9** and **20** were the most active COX-2 inhibitors (IC_50_ = 2.97 and 6.94 µM, respectively). In contrast, compounds **6** and **16** showed significantly low COX-2 inhibition (IC_50_ = 36.27 and 82.45 µM, respectively). Compounds with the 4-alkylphenyl moiety, such as the 4-tolyl fragment in compound **9** and the *N,N*-diethylaminophenyl fragment in compound **20**, have high COX-2 inhibition compared to compounds devoid of the 4-alkylphenyl moiety, such as compounds **6** and **16**.

**Table 7.** *In vitro* inhibitory effects of COX-2, EGFR, and HER2 of the antitumor agents **6, 9**, **16**, and **20**.^a^

Compound No.	IC_50_ (*µ*M)^a^		
	COX-2 Inhibition	EGFR Inhibition	HER2 Inhibition
**6**	36.27 ± 2.55	0.26 ± 0.09	0.35 ± 0.01
**9**	2.97 ± 0.07	0.77 ± 0.03	0.41 ± 0.01
**16**	82.45 ± 3.61	0.20 ± 0.07	0.13 ± 0.04
**20**	6.94 ± 0.41	0.19 ± 0.01	0.07 ± 0.02
Celecoxib	2.79 ± 0.07	–	–
Erlotenib	–	0.11 ± 0.04	0.09 ± 0.03
Sorafenib	–	0.10 ± 0.04	0.05 ± 0.02
Gefitinib	–	0.055 ± 0.99	0.079 ± 1.42

^a^IC_50_ value is the compound concentration required to produce 50% inhibition.

#### 2.4.2. Kinase inhibition activity

We tested the inhibitory effects of the most active compounds **6**, **9**, **16**, and **20** against EGFR and HER2^40^. We also tested the reference drugs erlotinib, sorafenib, and gefitinib. Table 7 summarises the inhibitory activities of compounds **6**, **9**, **16**, and **20**, and the reference drugs. The IC_50_ of erlotinib, sorafenib, and gefitinib against EGFR was 0.11, 0.10, and 0.055 µM, respectively, and against HER2 was 0.09, 0.05, and 0.079 µM, respectively. The IC_50_ of compounds **6**, **9**, **16**, and **20** against EGFR and HER2 were in the submicromolar range of **0.07–0.77 **µM. Compounds **16** and **20** showed the highest and most potent inhibitory activity against EGFR (IC_50_ = 0.20 and 0.19, respectively) and HER2 (IC_50_ = 0.13 and 0.07 µM, respectively) compared to erlotinib (EGFR-IC_50_ = 0.11 and HER2-IC_50_ = 0.09 µM), sorafenib (EGFR- IC_50_ = 0.10 and HER2-IC_50_ = 0.05 µM), and gefitinib (EGFR-IC_50_ = 0.055 and HER2-IC_50_ = 0.079 µM). Compound **9** was the least active against EGFR and HER2 (IC_50_ = 0.77 and 0.41 µM, respectively), while compound **6** was more effective against EGFR (IC_50_ = 0.26 µM) compared to HER2 (IC_50_ = 0.35 µM). Compound **20** showed inhibitory activity approximately similar to the reference drugs against EGFR and HER2. Compounds with 2-hydroxyphenyl fragments, such as compounds **16** and **20**, are more potent than corresponding compounds with the 4-tolyl moiety, such as compound **9**, or the 1-naphthyl fragment, such as compound **6.** In the enzymatic assay, the 2-hydroxyphenyl moiety plays a major role in the inhibitory activity against EGFR and HER2.

### 2.5. Molecular docking study

Molecular modelling is an important tool for studying the biological activity and SARs of bioactive compounds and exploring the binding mode of ligand molecules within the receptor- or putative enzyme-binding sites^77–80^. We performed molecular docking using the MOE 2008.10 program protocol obtained from Chemical Computing Group Inc. (Montreal, QC, Canada) ^81^. We subjected the selected compounds and the co-crystallized bound inhibitors to molecular docking into the putative active site of the protein to ensure docking accuracy and generate an appropriate binding orientation^27^^,^^40^^,^^41^^,^^82^^,^^83^.

#### 2.5.1. Molecular docking of compound 9 with COX-2

**Figure 6.**
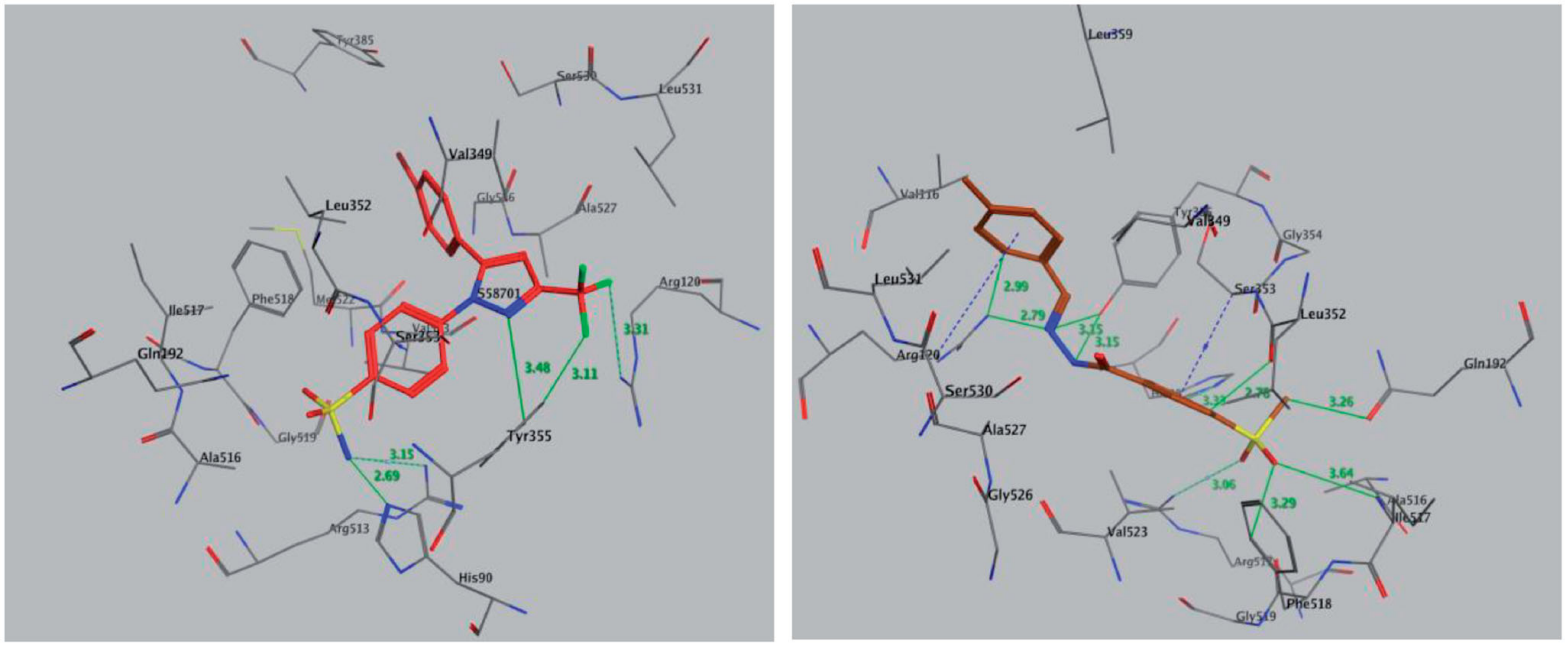
Binding mode of co-crystallized inhibitor (upper panel), compounds **9** (middle panel), and **20** (lower panel) within COX-2 binding site (PDB ID: 1CX2).

Molecular docking was performed to study the mode of interaction between the most active compound **9** and the COX-2 pocket–binding site (Figure 6). We derived the crystallographic binding site on the COX-2 isozyme in a complex with the SC-558 ligand, a celecoxib analogue, from the Protein Data Bank (PDB code: 1CX2) (Figure 6, left panel). We used the interaction energy and hydrogen bond formation among compound **9** and the amino acids within the putative active pocket of the COX-2 isozyme to predict the mode of interaction. Figure 6 shows the molecular docking results for compound **9**. Compound **9** was placed into the catalytic site of the COX-2 isozyme, where the pharmacophoric 4-methylsulfonylbenzene group can interact with amino acid residues Ile-517, Phe-518, His-90, Gln-192, and Arg-513 through a network of classical and nonclassical hydrogen bonds. These binding interactions were approximately similar to those of the SC-558 inhibitor co-crystallized in the COX-2-binding site. The stability of the docked complex of the target compound **9** in the COX-2-binding site depended on the methylsulfonyl pharmacophore (–SO_2_CH_3_) by forming classical and nonclassical hydrogen bonds with the key amino acid residues Arg-513 (3.06 Å), Gln-192 (3.26 Å), Ile-517 (3.64 Å), and Phe-518 (3.29 Å) (Figure 6, right panel), while the phenyl ring attached to the methylsulfonyl pharmacophore formed two nonclassical hydrogen bonds by binding with amino acid residues His-90 (3.33 Å) and Leu-352 (2.78 Å) and undergoing an additional CH–*π* interaction with amino acid residue Ser-353 (3.87 Å). Also, the benzylidenehydrazine fragment of compound **9** interacted with amino acid residues Ala-527, Val-349, Val-116, Leu-359, and Leu-531 through hydrophobic interactions. The hydrazino fragment of benzylidenehydrazine formed three classical hydrogen bonds with amino acid residues Arg-120 (2.79 Å), Tyr-355 (3.15, and 3.15 Å), while the phenyl part of the benzylidenehydrazine moiety interacted with the amino acid residue Arg-120 by forming a nonclassical H-bond (2.99 Å) and CH–*π* interaction (3.98 Å) (Figure 6, right panel).

#### 2.5.2. Molecular docking of compound 20 with EGFR

**Figure 7.**
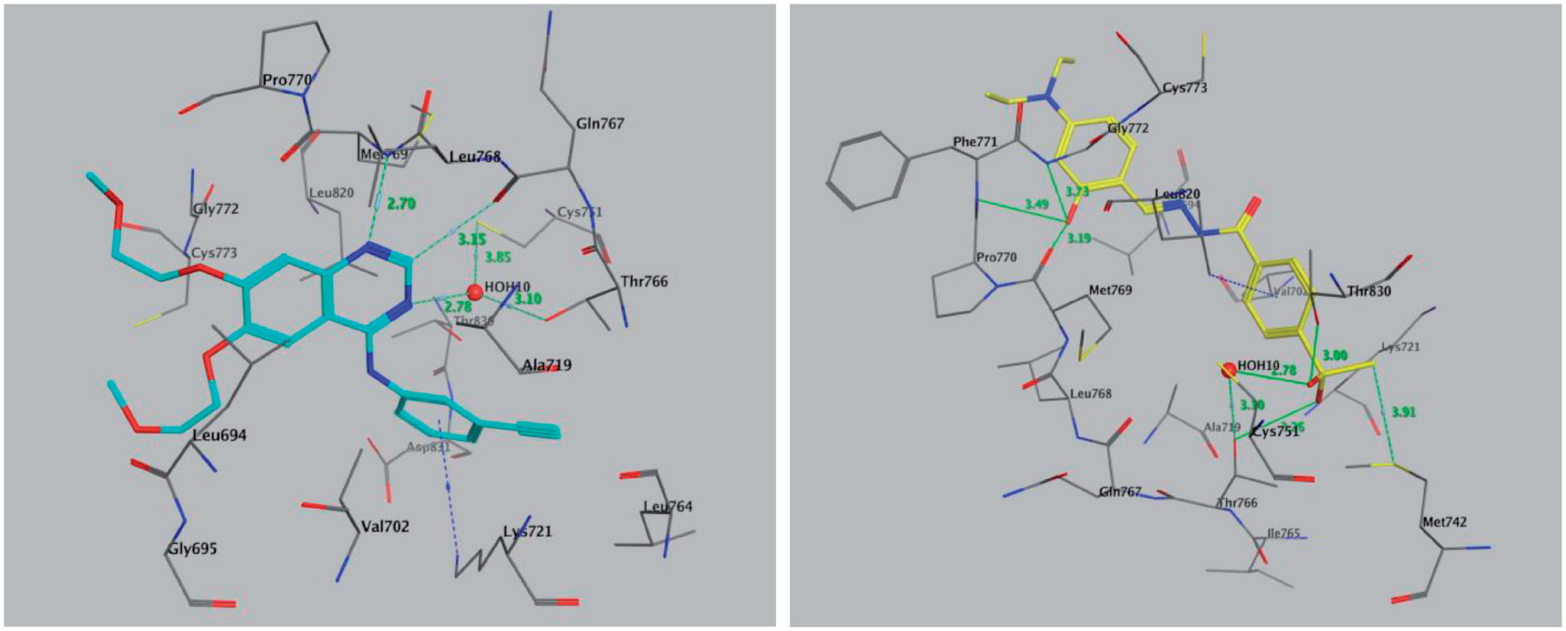
Binding mode of co-crystallized inhibitor (left panel) and compounds **20** (right panel) within EGFR binding site (PDB ID: 1M17).

The results of the antitumor activity and enzymatic assay of compound **20** against EGFR prompted us to perform molecular docking studies of the ATP-binding site of EGFR, along with the reference drug erlotinib to predict the binding interactions of the target compound (Figure 7). We retrieved the ligand erlotinib from the PDB as a co-crystallized ligand in a complex with EGFR (PDB code: 1M17) (Figure 7, left panel). Both phenyl rings of compound **20** surrounded and interacted with amino acid residues lining the hydrophobic pocket in EGFR-TK, such as Gly-772, Leu-768, Pro-770, Leu-694, Leu-820, and Val-702 (Figure 7, right panel). Also, the –OH group of compound **20** formed triple hydrogen bonds with amino acid residues Met-769 (3.19 Å), Pro-770 (3.49 Å), and Gly-772 (3.73 Å). The methylsulfonyl (CH_3_-SO_2_–) moiety showed significant interactions, where the oxygen atom of the CH_3_-SO_2_– group directly formed hydrogen bonds with the amino acid residue Thr-766 (3.26 Å) and the Thr-830 side chain (3.00 Å) and showed additional binding with a water molecule (HOH-10)-mediated hydrogen bonding with Thr-766 (2.78, and 3.10 Å). Also, the methyl moiety of the CH_3_-SO_2_– group interacted with amino acid residue Met-742 by a nonclassical hydrogen bond of 3.91 Å with the sulphur (S-) part of Met-742, while the phenyl part attached to the methylsulfonyl moiety interacted with amino acid residue Leu-820 through CH–π interaction (4.34 Å). These binding interactions indicated that both 2-hydroxyphenyl and 4-methylsulfonylbenzene fragments are important for binding and subsequent inhibitory effects (Figure 7).

#### 2.5.3. Molecular docking of compound 20 with HER2

**Figure 8.**
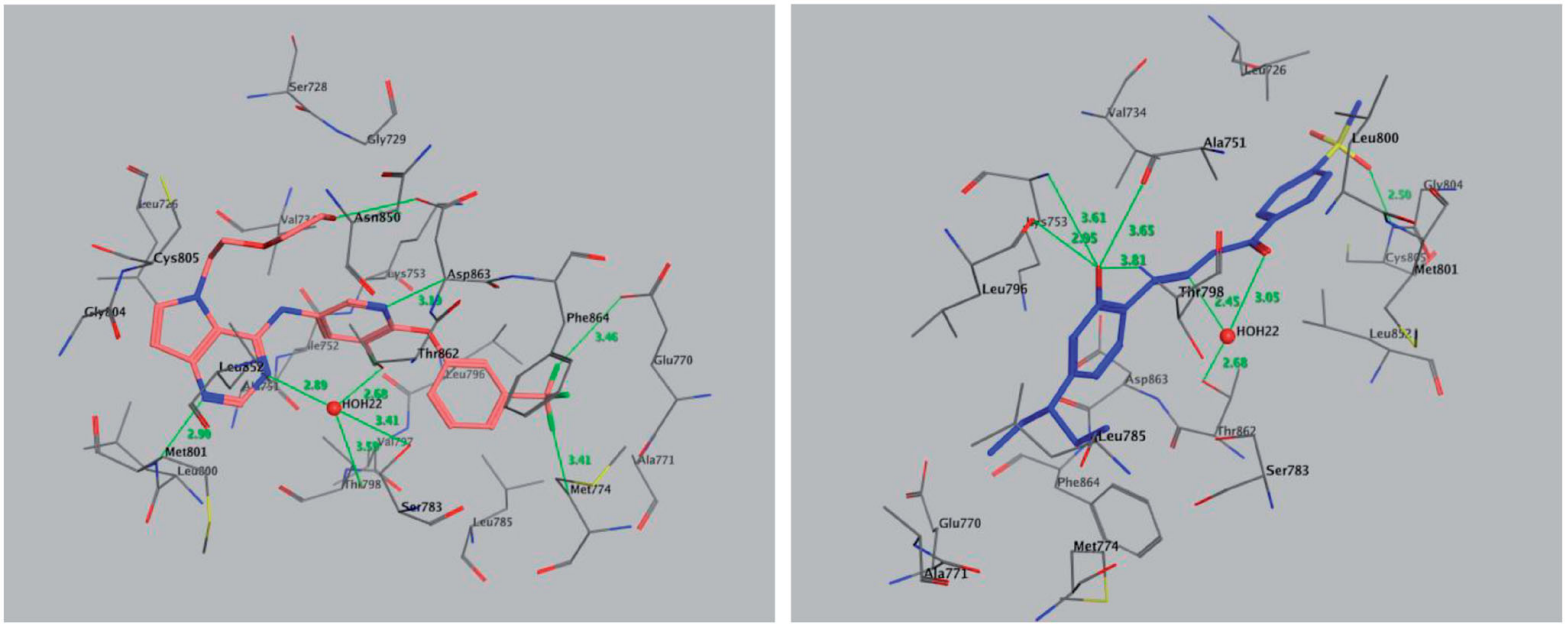
Binding mode of co-crystallized inhibitor (left panel) and compounds **20** (right panel) within HER2 binding site (PDB ID: 3PP0).

We retrieved the crystal 3 D structure of HER2 co-crystallized with its bound inhibitor 03Q from the PDB (PDB code: 3PP0) (Figure 8, left panel). Molecular docking of compound **20** into the HER2-binding cavity showed that the 2-hydroxyphenyl fragment forms four hydrogen bonds with amino acid residues Leu-796 (2.95 Å), Thr-798 (3.81 Å), Ala-751 (3.65 Å), and Lys-753 (3.61 Å), while the hydrazide fragment forms two hydrogen bonds with a water molecule (HOH-22)-mediated hydrogen bonding with Thr-862 (2.45, and 3.05 Å) (Figure 8, right panel). In addition, we observed one more hydrogen bond between one oxygen of the sulphonyl group and the amino acid residue Cys-805 (2.50 Å). In contrast, the *N,N*-diethylaminophenyl moiety of compound **20** shows hydrophobic interactions with the side chains of amino acid residues Glu-770, Ser-783, Leu-785, Met-774, Phe-864, Leu-796, Thr-798, Asp-863, and Lys-753, while the methylsulfonylbenzene moiety showed hydrophobic interactions with amino acid residues Leu-852, Met-801, Leu-800, Val-734, Leu-826, and Gly-804. The binding modes of compound **20** are approximately similar to the co-crystallized bound inhibitor with HER2 kinase.

### 2.6. Physicochemical and pharmacokinetic predictions

**Figure 9.**
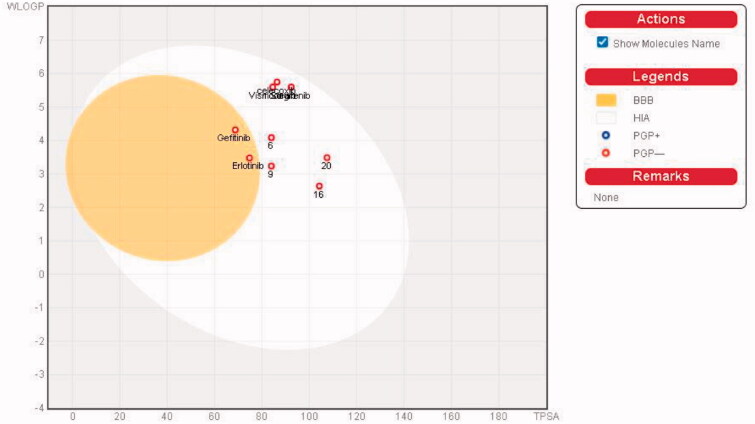
Boiled-Egg plot predicted by swissADME online web tool for target molecules **6**, **9, 16**, **20**, and the reference drugs (celecoxib, erlotinib, gefitinib, and vismodegib).

**Figure 10.**
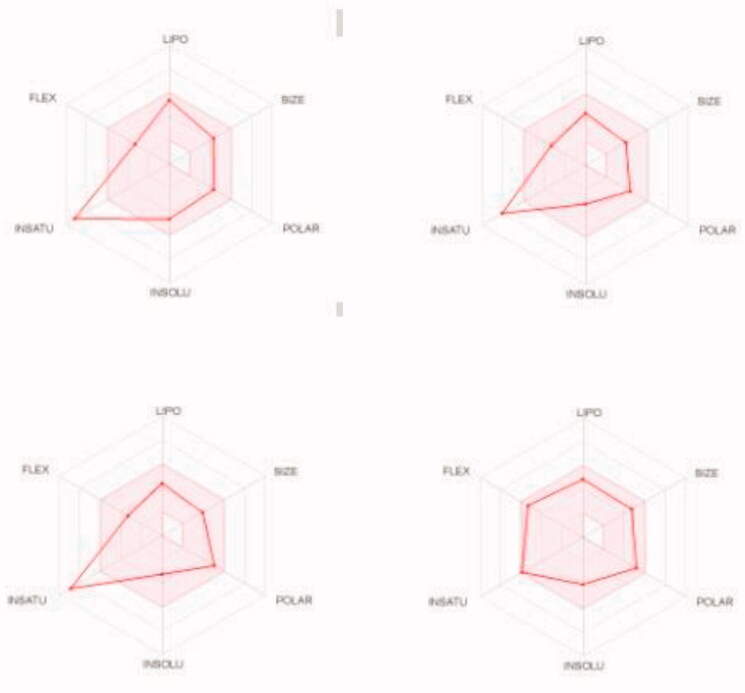
Bioavailability radar charts as predicted by swissADME online web tool for target molecules **6** (upper left panel), **9** (upper right panel), **16** (lower left panel), and **20** (lower right panel).

**Figure 11.**
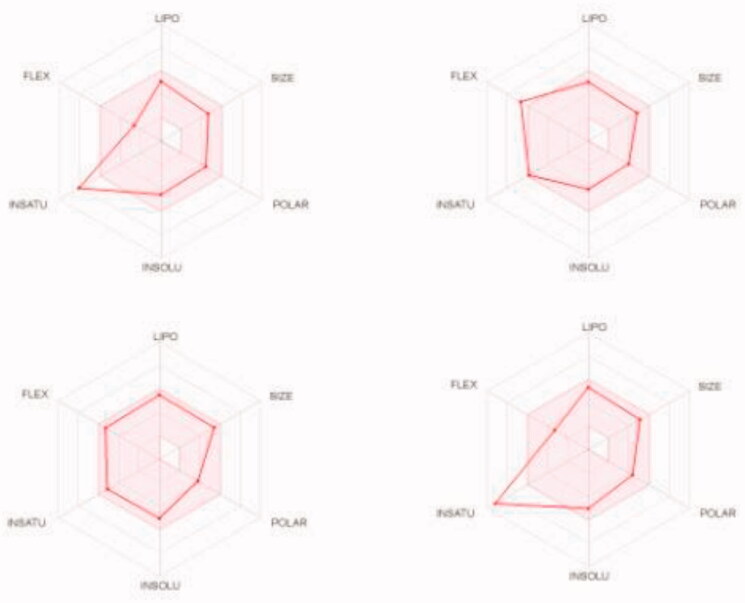
Bioavailability radar charts as predicted by swissADME online web tool for reference drugs celecoxib (upper left panel), erlotinib (upper right panel), gefitinib (lower left panel), and vismodegib (lower right panel).

We predicted the pharmacokinetic and physicochemical properties of the most active compounds **6**, **9**, **16**, **20**, and reference drugs celecoxib, erlotinib, gefitinib, and vismodegib using the automated SwissADME online calculation system (Table 8)^84^. Compounds **6**, **9**, **16**, **20** showed high gastrointestinal absorption, while compound **20** was predicted as an inhibitor of CYP2C19, CYP2C9, and CYP3A4 isoforms and a non-inhibitor of CYP1A2 and CYP2D6 isoforms (Table 8). In contrast, compound **16** was predicted as a non-inhibitor of all CYP isoforms. In addition, compounds **6** and **9** were predicted as inhibitors of CYP2C19 and CYP1A2 isoforms and non-inhibitors of CYP2C9, CYP3A4, and CYP2D6 isoforms. In addition, we calculated the drug-likeness properties, as indicated by major Lipinski’s (Pfizer), Ghose’s (Amgen), Veber’s (GSK), and Egan’s (Pharmacia) pharmaceutical rules^85–87^. Compounds **6**, **9**, **16**, **20** successfully passed all filters (Table 8). The BOILED-Egg graph^88^ of the Wlog*P*/*t*PSA (topological polar surface area) showed that compounds **6**, **9**, **16**, **20**, together with celecoxib and vismodegib, are located in the human intestinal absorption (HIA) region with no BBB permeation, indicating few CNS side effects (Figure 9). Indeed, compounds **6**, **9**, **16**, **20** are not P-glycoprotein (P-gp−) substrates, suggesting that they are not susceptible to the efflux mechanism carried out by P-gp that is used by many cancer cell lines as a drug resistance mechanism (Figure 9)^89–91^. In addition, the bioavailability radar chart of compounds **6**, **9**, **16**, **20**, and reference drugs are shown in Figures 10 and 11. These charts were drawn as six axes for six key properties of oral bioavailability: polarity (POLAR), solubility (INSOLU), lipophilicity (LIPO), flexibility (FLEX), saturation (INSATU), and size (SIZE) ^89–91^. The optimal property ranges are shown as a pink area, while the red line represents predicted properties for the examined molecule. The SwissADME tool calculation of compounds **6**, **9**, **16**, and **20** predicts that they possess appropriate physicochemical and pharmacokinetic properties.

**Table 8.** Predictions of the physicochemical and pharmacokinetic properties for target compounds **6**, **9**, **16**, and **20** together with reference drugs^a^

Compounds No.	6	9	16	20	celecoxib	Erlotinib	Gefitinib	Vismodegib
Properties								
BBB^b^	NO	NO	NO	NO	NO	Yes	Yes	NO
GIA^b^	High	High	High	High	High	High	High	High
P-gp^b^substrate	No	No	No	No	No	No	No	No
CYP1A2 inhibitor^b^	Yes	Yes	No	No	No	Yes	No	No
CYP2C19 inhibitor^b^	Yes	Yes	No	Yes	No	Yes	Yes	Yes
CYP2C9 inhibitor^b^	No	No	No	Yes	Yes	Yes	Yes	Yes
CYP2D6 inhibitor^b^	No	No	No	No	No	Yes	Yes	No
CYP3A4 inhibitor^b^	No	No	No	Yes	No	Yes	Yes	Yes
Log S (Water Solubility)	−6.96 (Poorly soluble)	−5.69 (Moderately soluble)	−4.73 (Moderately soluble)	−5.60 (Moderately soluble)	−6.22 (Poorly soluble)	−7.26 (Poorly soluble)	−7.94 (Poorly soluble)	−8.51 (Poorly soluble)
Bioavailability Score	0.55	0.55	0.55	0.55	0.55	0.55	0.55	0.55
Lipinski #violations	0	0	0	0	0	0	0	0
Ghose #violations	0	0	0	0	1	0	0	1
Veber #violations	0	0	0	0	0	0	0	0
Egan #violations	0	0	0	0	0	0	0	0

^a^All calculations were performed using SwissADME.

^b^GIA: gastrointestinal absorption; BBB: blood-brain barrier permeation; P-gp: permeability glycoprotein; CYP1A2, CYP2C9, CYP2C19, CYP3A4 and CYP2D6 are isoforms of cytochromes P450.

## 3. Conclusion

We synthesised a series of hydrazones **4–24** based on a 4-methylsulfonylbenzene scaffold starting from 4-methylsulfonylphenylhydrazide. We also analysed the potential antitumor activities of the 21 hydrazones using 59 human cell lines and performed enzyme inhibition assays using EGFR, HER2, and COX-2. Compounds **6**, **9**, **16**, and **20** possess the highest broad-spectrum and potent antitumor activity with a GI of >10–100% and a PCE of 52/59, 27/59, 59/59, and 59/59, respectively, for nine human tissue compared to imatinib (GI = >10%–47.1%; PCE = 20/55). The antitumor activity of compounds **6**, **9**, **16**, and **20** against individual organs is GI = 20.8–100% for leukaemia, 12.7%–96.7% for NSCLC, 13.9%–91.4% for colon cancer, 10.3%–96.0% for CNS cancer, 10.6–100% for melanoma, 12.3%–83.2% for ovarian cancer, 12.3–100% for renal cancer, 11.2%–75.5% for prostate cancer, and 12.4%–98.8% for breast cancer. Compound **20** has the highest GI and shows significant antitumor activity (GI_50_ = 0.063–11.7 µM) compared to celecoxib, erlotinib, gefitinib, sorafenib, and vismodegib (GI_50_ = 3.98–63.09, 0.10–100.0, 0.0125–10.0, 1.26–3.98, and 19.95–100.0 µM, respectively). Cell cycle analysis showed that the apoptotic mechanism underlying programmed cell death is the main mechanism rather than the necrotic pathway, and compounds **6**, **9**, **16**, and **20** induce apoptosis by inhibiting cell growth at the G2/M phase. In addition, compounds **9** and **20** are the most active inhibitory agents against COX-2 (IC_50_ = 2.97 and 6.94 µM, respectively) compared celecoxib (IC_50_ = 2.79 µM), while compounds **16** and **20** have the highest inhibition activity against EGFR (IC_50_ = 0.2 and 0.19 µM, respectively) and HER2 (IC_50_ = 0.13 and 0.07 µM, respectively) compared to erlotinib (EGFR-IC_50_ = 0.11 and HER2-IC_50_ = 0.09 µM), sorafenib (EGFR-IC_50_ = 0.10 and HER2-IC_50_ = 0.05 µM), and gefitinib (EGFR-IC_50_ = 0.055 and HER2-IC_50_ = 0.079 µM). The results as mentioned above indicated that these compounds are potential multitarget agents as COX-2, EGFR, and HER2 inhibitors. Compound **9** was subjected to molecular docking into binding sites of COX-2, while compound **20** was subjected to molecular docking into the putative binding sites of EGFR and HER2 to find the binding mode and molecular models required for interaction of these compounds with respective enzymes or receptors. Molecular docking showed that the respective molecules bound approximately similar to the co-crystallized inhibitors in COX-2-, EGFR-, and HER2-binding sites. SwissADME and drug-likeness prediction showed that compounds **6**, **9**, **16**, and **20** possess good physicochemical, pharmacokinetic, and drug-likeness properties.

## 4. Experimental

### 4.1. Chemistry

Melting points (uncorrected) were recorded on a Barnstead 9100 Electrothermal melting apparatus (APS Water Services Corporation, Van Nuys, CA, USA), while the IR spectra were recorded on a FT-IR Perkin-Elmer spectrometer (PerkinElmer Inc., Waltham, MA, USA). The ^1^H NMR and ^13 ^C NMR were measured in DMSO-d_6_ or CDCl_3_, on Bruker 700 or 500 and 176 or 125 MHz instruments, respectively (Bruker, Billerica, MA, USA). Chemical shifts are reported in δ ppm. Mass spectra were recorded on an Agilent 6320 Ion Trap mass spectrometer (Agilent Technologies, Santa Clara, CA, USA). C, H, and N were analysed at the Research Centre, College of Pharmacy, King Saud University, Saudi Arabia. The results were within ± 0.4% of the theoretical values. Compounds **3**, **11**, **16**, **19**, and **20** were prepared according to a previous report^65^.

#### 4.1.1. General procedure for the synthesis of hydrazones 4–24 (Scheme 1)

A mixture of 4-(methylsulfonyl)benzohydrazide **3** (10 mmol) and an appropriate aromatic aldehyde (10 mmol) was stirred in methanol (10 ml) containing a catalytic amount of acetic acid (0.5 ml) at room temperature for 24 h. The obtained solid was filtered, dried, and recrystallized from absolute ethanol.

##### 4.1.1.1. N'-Benzylidene-4-(methylsulfonyl)benzohydrazide (4)

M.p 273–275^ο^; 95% yield; IR (KBr, cm^−1^) *ν*: 3215 (NH), 1656 (C = O), 1284, 1148 (O = S=O); ^1^H NMR (700 MHz, DMSO-d_6_): δ 12.10 (s, 1H), 8.48 (s, 1H), 8.16 (d, 2H, *J* = 7.77 Hz), 8.11 (d, 2H, *J* = 7.77 Hz), 7.77 (d, 2H, *J* = 7.14 Hz), 7.48 (dd, 3H, *J* = 19.95 & 7.14 Hz), 3.31 (s, 3H); ^13 ^C NMR (176 MHz, DMSO-d_6_): δ 162.3, 149.2, 143.7, 138.3, 134.5, 130.8, 129.3, 129.1, 127.7, 127.6, 43.7; C_15_H14N2O3S: m/z (302.1).

##### 4.1.1.2. 4-(Methylsulfonyl)-N'-(pyridin-3-ylmethylene)benzohydrazide (5)

M.p 339–341^ο^; 89% yield; IR (KBr, cm^−1^) *ν*: 3221 (NH), 1640 (C=O), 1339, 1147 (O=S=O); ^1^H NMR (700 MHz, DMSO-d_6_): δ 12.26 (s, 1H), 8.89 (s, 1H), 8.64 (d, 1H, *J* = 4.55 Hz), 8.53 (s, 1H), 8.16 (d, 3H, *J* = 10.57, 8.33 Hz), 8.11 (d, 2H, *J* = 8.12 Hz), 7.51 (dd, 1H, *J* = 12.53, 2.80 Hz), 3.31 (s, 3H); ^13 ^C NMR (176 MHz, DMSO-d_6_): δ 162.4, 151.4, 149.3, 146.4, 143.8, 138.1, 134.0, 130.4, 129.1, 127.6, 124.5, 43.7; C14H13N3O3S: m/z (303.3).

##### 4.1.1.3. 4-(Methylsulfonyl)-N'-(naphthalen-1-ylmethylene)benzohydrazide (6)

M.p 235–237^ο^; 88% yield; IR (KBr, cm^−1^) *ν*: 3222 (NH), 1651 (C=O), 1295, 1144 (O = S=O); ^1^H NMR (700 MHz, DMSO-d_6_): δ 12.18 (s, 1H), 9.12 (s, 1H), 8.05 (dd, 1H, *J* = 15.47 & 8.19 Hz), 8.21 (d, 2H, *J* = 7.63 Hz), 8.14 (d, 2H, *J* = 7.63 Hz), 8.05 (dd, 2H, *J* = 15.47 & 8.19 Hz), 7.97 (d, 1H, *J* = 7.07 Hz), 7.70 (t, 1H, *J* = 15.12 Hz), 7.63 (dd, 2H, *J* = 7.09 Hz), 3.32 (s, 3H); ^13 ^C NMR (176 MHz, DMSO-d_6_): δ 162.3, 149.1, 143.8, 138.3, 134.0, 131.3, 130.6, 129.7, 129.3, 129.1, 128.6, 127.9, 127.7, 126.8, 126.0, 124.7, 43.7; C19H16N2O3S: m/z (352.1).

##### 4.1.1.4. N'-(4-Chlorobenzylidene)-4-(methylsulfonyl)benzohydrazide (7)

M.p 268–270^ο^; 92% yield; IR (KBr, cm^−1^) *ν*: 3239 (NH), 1656 (C=O), 1284, 1145 (O = S=O); ^1^H NMR (700 MHz, DMSO-d_6_): δ 12.16 (s, 1H), 8.46 (s, 1H), 8.16 (d, 2H, *J* = 8.26 Hz), 8.11 (d, 2H, *J* = 8.33 Hz), 7.78 (d, 2H, *J* = 8.40 Hz), 7.54 11 (d, 2H, *J* = 8.33 Hz), 3.30 (s, 3H); ^13 ^C NMR (176 MHz, DMSO-d_6_): δ 162.4, 147.8, 143.8, 138.2, 135.2, 133.4, 129.4, 129.3, 129.1, 127.6, 43.7; C15H13ClN2O3S m/z: 336.0, (M + 2; 338.0).

##### 4.1.1.5. N'-(4-Fluorobenzylidene)-4-(methylsulfonyl)benzohydrazide (8)

M.p 278–280^ο^; 90% yield; IR (KBr, cm^−1^) *ν*: 3239 (NH), 1655 (C=O), 1285, 1146 (O = S=O); ^1^H NMR (700 MHz, DMSO-d6): δ 12.17 (s, 1H), 8.48 (s, 1H), 8.16 (d, 2H, *J* = 7.98 Hz), 8.11 (d, 2H, *J* = 5.39 Hz), 7.82 (t, 2H, *J* = 13.09 Hz), 7.32 (t, 2H, *J* = 8.43 Hz), 3.31 (s, 3H); ^13 ^C NMR (176 MHz, DMSO-d_6_): δ 164.4, 163.0, 162.3, 148.0, 143.7, 138.3, 131.2, 131.1, 130.7, 129.9, 129.8, 129.1, 127.6, 116.5, 116.3, 43.7; C15H13FN2O3S: m/z: 320.2.

##### 4.1.1.6. N'-(4-Methylbenzylidene)-4-(methylsulfonyl)benzohydrazide (9)

M.p 271–272^ο^; 94% yield; IR (KBr, cm^−1^) *ν*: 3199 (NH), 1652 (C=O), 1292, 1150 (O = S=O); ^1^H NMR (700 MHz, DMSO-d6): δ 12.04 (s, 1Η), 8.44 (s, 1Η), 8.16 (d, 2H, *J* = 8.12 Hz), 8.11 (d, 2H, *J* = 8.19 Hz), 7.65 (d, 2H, *J* = 7.84 Hz), 7.29 (d, 2H, *J* = 7.84 Hz), 3.30 (s, 3H), 2.35 (s, 3H); ^13 ^C NMR (176 MHz, DMSO-d_6_): δ 162.3, 149.2, 143.7, 140.7, 138.4, 131.8, 129.9, 129.1, 127.7, 127.6, 43.7, 21.5; C16H16N2O3S m/z: 316.4.

##### 4.1.1.7. N'-(4-Methoxybenzylidene)-4-(methylsulfonyl)benzohydrazide (10)

M.p 249–251^ο^; 88% yield; IR (KBr, cm^−1^) *ν*: 3212 (NH), 1698 (C=O), 1282, 1149 (O = S=O); ^1^H NMR (700 MHz, DMSO-d6): δ 12.08 (s, 0.25Η), 11.97 (s, 0.75 H), 8.84 (s, 0.25H), 8.42 (s, 0.75H), 8.18 (d, 0.58H, *J* = 8.12 Hz), 8.15 (d, 1.42H, *J* = 8.12 Hz), 8.10 (d, 1.77H, *J* = 8.12 Hz), 8.01 (d, 0.23 H, J = 7.91 Hz), 7.90 (d, 0.3H, *J* = 7.56 Hz), 7.71 (d, 1.7H, J = 8.12 Hz), 7.13 (d, 0.3H, *J* = 8.33 Hz), 7.04 (d, 1.7H, *J* = 8.33 Hz), 3.88 (s, 0.8H), 3.82 (s, 2.2H), 3.30 (s, 3H); ^13 ^C NMR (176 MHz, DMSO-d_6_): δ 162.1, 161.4, 158.3, 149.0, 144.9, 144.6, 143.7, 143.6, 138.5, 138.3, 129.3, 129.1, 129.0, 127.6, 127.0, 114.8, 56.1, 55.7, 43.75; C16H16N2O4S m/z: 332.3.

##### 4.1.1.8. N'-(4-(Dimethylamino)benzylidene)-4-(methylsulfonyl)benzohydrazide (12)

M.p 295–297^ο^; 85% yield; IR (KBr, cm^−1^) *ν*: 3488 (NH), 1655 (C=O), 1278, 1148 (O = S=O); ^1^H NMR (700 MHz, DMSO-d6): δ 11.81 (s, 1H), 8.34 (s, 1H), 8.14 (d, 2H, *J* = 7.77 Hz), 8.09 (d, 2H, *J* = 7.91 Hz), 7.57 (d, 2H, *J* = 8.12 Hz), 7.63 (d, 2H, *J* = 8.19 Hz), 3.30 (s, 3H), 2.97 (s, 5.3H), 2.92 (s, 0.7H); ^13 ^C NMR (176 MHz, DMSO-d_6_): δ 161.8, 152.1, 150.0, 143.5, 138.7, 130.7, 129.1, 129.0, 127.6, 121.7, 112.2, 49.0, 43.7; C17H19N3O3S m/z: 345.1.

##### 4.1.1.9. N'-(2-Chlorobenzylidene)-4-(methylsulfonyl)benzohydrazide (13)

M.p 346–348^ο^; 85% yield; IR (KBr, cm^−1^) *ν*: 3278 (NH), 1684 (C=O), 1271, 1142 (O = S=O); ^1^H NMR (700 MHz, DMSO-d6): δ 12.31 (s, 1H), 8.89 (s, 1H), 8.19 (s, 1H), 8.18 (d, 2H, *J* = 8.12 Hz), 8.11 (d, 2H*, J* = 8.19 Hz), 8.05 (d, 2H, *J* = 7.14 Hz), 7.55 (d, 1H, *J* = 7.56 Hz), 7.47 (p, 2H, *J* = 16.31, 7.14 & 7.0 Hz), 3.31 (s, 3H); ^13 ^C NMR (176 MHz, DMSO-d_6_): δ 162.4, 145.0, 143.9, 138.0, 133.8, 132.2, 131.8, 130.4, 129.2, 128.1, 127.6, 127.4, 43.7; m/z: C15H13ClN2O3S m/z 336.0, (M + 2; 338.0).

##### 4.1.1.10. N'-(2-Fluorobenzylidene)-4-(methylsulfonyl)benzohydrazide (14)

M.p 300–302^ο^; 88% yield; IR (KBr, cm^−1^) *ν*: 3211 (NH), 1654 (C=O), 1280, 1144 (O = S=O); ^1^H NMR (700 MHz, DMSO-d6): δ 12.21 (s, 1H), 8.72 (s, 1H), 8.18 (d, 2H*, J* = 8.12 Hz), 8.11 (d, 2H, *J* = 8.12 Hz), 7.98 (t, 1H, *J* = 7.31 Hz), 7.52 (dd, 1H, *J* = 6.72, 6.93 Hz), 7.33 (d, 2H, *J* = 7.98 Hz), 3.31 (s, 3H); ^13 ^C NMR (176 MHz, DMSO-d_6_): δ 162.3, 162.0, 160.6, 143.9, 141.9, 141.8, 138.1, 132.8, 132.7, 129.1, 127.7, 126.8, 125.4, 122.1, 122.0, 116.5, 43.7; C15H13FN2O3S m/z: 320.2.

##### 4.1.1.11. N'-(2-Methoxybenzylidene)-4-(methylsulfonyl)benzohydrazide (15)

M.p 333–335^ο^; 91% yield; IR (KBr, cm^−1^) *ν*: 3198 (NH), 1679 (C=O), 1276, 1145 (O = S=O); ^1^H NMR (700 MHz, DMSO-d6): δ 12.08 (s, 1H), 8.84 (s, 1H), 8.18 (d, 2H, J = 7.56 Hz), 8.09 (d, 2H, J = 7.63 Hz), 7.90 (d, 1H, J = 7.70 Hz), 7.44 (t, 1H, J = 7.73 Hz), 7.13 (d, 1H, J = 8.33 Hz), 7.04 (t, 1H, J = 7.42 Hz), 3.82 (s, 3H), 3.31 (s, 3H); ^13 ^C NMR (176 MHz, DMSO-d_6_): δ 162.1, 158.3, 144.6, 143.7, 138.3, 132.3, 129.1, 127.6, 126.0, 122.5, 121.2, 112.3, 56.1, 43.7; C16H16N2O4S m/z: 332.1.

##### 4.1.1.12. N'-(2,4-Dichlorobenzylidene)-4-(methylsulfonyl)benzohydrazide (17)

M.*p* > 350^ο^; 93% yield; IR (KBr, cm^−1^) *ν*: 3227 (NH), 1677 (C=O), 1294, 1150 (O = S=O); ^1^H NMR (700 MHz, DMSO-d6): δ 12.35 (s, 1H), 8.83 (s, 1H), 8.18 (d, 2H, *J* = 7.84 Hz), 8.11 (d, 2H, *J* = 7.77 Hz), 8.05 (d, 1H, *J* = 8.47 Hz), 7.75 (s, 1H), 7.55 (d, 1H, *J* = 8.47 Hz), 3.31 (s, 3H); ^13 ^C NMR (176 MHz, DMSO-d_6_): δ 162.4, 143.9, 137.9, 135.8, 134.5, 130.9, 129.9, 129.2, 128.6, 127.7, 43.7; C15H12Cl2N2O3S: m/z 371.0, (M + 2; 373).

##### 4.1.1.13. N'-(3,4-Dichlorobenzylidene)-4-(methylsulfonyl)benzohydrazide (18)

M.p 242–244^ο^; 87% yield; IR (KBr, cm^−1^) *ν*: 3282 (NH), 1688 (C=O), 1274, 1146 (O = S=O); ^1^H NMR (700 MHz, DMSO-d6): δ 12.29 (s, 1H), 8.44 (s, 1H), 8.15 (d, 2H, *J* = 8.05 Hz), 8.11 (d, 2H, *J* = 8.05 Hz), 7.99 (s, 1H), 7.76 (dd, 2H, *J* = 8.12 & 7.84 Hz), 3.30 (s, 3H); ^13 ^C NMR (176 MHz, DMSO-d_6_): δ 162.5, 146.4, 143.9, 138.0, 135.4, 132.9, 132.2, 131.6, 129.2, 129.1, 127.6, 127.4, 43.7; C15H12Cl2N2O3S: m/z 371.0, (M + 2; 373).

##### 4.1.1.14. N'-(3,4-Dimethoxybenzylidene)-4-(methylsulfonyl)benzohydrazide (21)

M.p 245–247^ο^; 90% yield; IR (KBr, cm^−1^) *ν*: 3212 (NH), 1657 (C=O), 1269, 1140 (O = S=O); ^1^H NMR (700 MHz, DMSO-d_6_): δ 11.97 (s, 1H), 8.39 (s, 1H), 8.14 (d, 2H, *J* = 8.19 Hz), 8.09 (d, 2H, *J* = 8.19 Hz), 7.37 (s, 1H), 7.24 (d, 1H, *J* = 8.05 Hz), 7.05 (d, 1H, *J* = 8.26 Hz), 3.83 (s, 3H), 3.82 (s, 3H), 3.30 (s, 3H); ^13 ^C NMR (176 MHz, DMSO-d_6_): δ 172.5, 162.2, 151.4, 149.5, 149.3, 143.6, 138.5, 129.0, 127.6, 122.6, 111.9, 108.6, 56.0, 55.9, 43.7; C17H18N2O5S m/z: 362.3.

##### 4.1.1.15. N'-(Benzo[d][1,3]dioxol-5-ylmethylene)-4-(methylsulfonyl)benzohydrazide (22)

M.p 289–291^ο^; 86% yield; IR (KBr, cm^−1^) *ν*: 3220 (NH), 1655 (C=O), 1275, 1145 (O = S=O); ^1^H NMR (700 MHz, DMSO-d_6_): δ 12.00 (s, 1H), 8.38 (s, 1H), 8.14 (d, 2H, *J* = 8.19 Hz), 8.09 (d, 2H, *J* = 8.19 Hz), 7.33 (s, 1H), 7.20 (d, 1H, *J* = 7.91 Hz), 7.02 (d, 1H, *J* = 7.91 Hz), 6.11 (s, 2H), 3.30 (s, 3H); ^13 ^C NMR (176 MHz, DMSO-d_6_): δ 162.2, 149.7, 148.9, 148.5, 143.7, 138.4, 129.0, 128.9, 127.6, 124.1, 108.9, 105.6, 102.1, 43.7; C16H14N2O5S m/z: 346.2.

##### 4.1.1.16. 4-(Methylsulfonyl)-N'-(3,4,5-trimethoxybenzylidene)benzohydrazide (23)

M.p 228–230^ο^; 95% yield; IR (KBr, cm^−1^) *ν*: 3199 (NH), 1668 (C=O), 1272, 1132 (O = S=O); ^1^H NMR (700 MHz, DMSO-d_6_): δ 12.09 (s, 1H), 8.40 (s, 1H), 8.14 (d, 2H, *J* = 8.19 Hz), 8.09 (d, 2H, *J* = 8.05 Hz), 7.06 (s, 2H), 3.85 (s, 6H), 3.72 (s, 3H), 3.30 (s, 3H), 13 C NMR (176 MHz, DMSO-d6): δ 162.4, 153.6, 149.1, 143.7, 139.8, 138.4, 130.0, 129.1, 127.6, 1104.8, 60.6, 56.4, 43.7; C18H20N2O6S m/z: 392.1.

##### 4.1.1.17. (4-(Methylsulfonyl)-N'-(2,4,5-trimethoxybenzylidene)benzohydrazide (24)

M.p 277–279^ο^; 94% yield; IR (KBr, cm^−1^) *ν*: 3209 (NH), 1667 (C=O), 1275, 1140 (O = S=O); ^1^H NMR (700 MHz, DMSO-d_6_): δ 11.96 (s, 1H), 8.77 (s, 1H), 8.17 (d, 2H, *J* = 8.12 Hz), 8.09 (d, 2H, *J* = 8.12 Hz), 7.38 (s, 1H), 6.76 (s, 1H), 3.88 (s, 3H), 3.87 (s, 3H), 3.77 (s, 3H), 3.30 (s, 3H), ^13^C NMR (176 MHz, DMSO-d_6_): δ 161.8, 154.0, 152.7, 144.8, 143.7, 143.6, 138.4, 129.0, 127.5, 113.7, 107.9, 98.2, 56.9, 56.3, 56.2, 43.7; C18H20N2O6S m/z: 392.1.

### 4.2. Biological evaluation

#### 4.2.1. *In vitro* antitumor assay

The antitumor assay was performed for 59 human tumour cell lines obtained from nine human tissue under the protocol of the Drug Evaluation Branch, National Cancer Institute, Bethesda, MD_._ Three dose-response parameters; GI_50_, TGI, and LC_50_; were calculated for each compound^17^^,^^40^^,^^70^.

#### 4.2.2. Apoptosis assay

According to our previous report, apoptosis induction was performed using the Leukaemia HL-60 cell line and well-established Annexin 5-FITC/PI detection kit. The cell line samples were analysed using FACSCalibur flow cytometer^40^^,^^72^.

#### 4.2.3. Cell cycle analysis

Cell cycle analysis was carried out similar to our previous report using the Leukaemia HL-60 cell line stained with the DNA fluorochrome PI and analysed by FACSCalibur flow cytometer^40^^,^^74^.

#### 4.2.4. *In vitro* cyclooxygenase (COX) inhibition assay

The colorimetric COX-2 inhibition assay (kit catalogue number 560101, Cayman Chemical, Ann Arbour, MI) was used to measure the ability of the tested derivatives and celecoxib to inhibit COX-2 isozyme under the manufacturer’s instructions^15^^,^^76^ .

#### 4.2.5. Egfr and HER2 tyrosine kinases assay

*In vitro* luminescent EGFR tyrosine kinase assay using Kinase-Glo® MAX as a detection reagent, and *In vitro* HER2 tyrosine kinase assay using DP‐Glo™ reagent that measures ADP formed from a kinase reaction, this luminescent signal positively correlates with ADP amount and kinase activity^40^.

### 4.3. Molecular docking and ADME methodology

Molecular docking protocols were carried out using the MOE 2008.10 software from Chemical Computing Group Inc. (Montreal, QC, Canada) following established methods^40^^,^^81^. The crystal structures of COX-2 (PDB code: 1CX2), EGFR (PDB Code: 1M17), and HER2 (PDB Code: 3PP0) were retrieved from the protein data bank. The Swiss Target Prediction and the Swiss ADME online tools were used to predict the physicochemical, pharmacokinetic, and drug-likeness properties of the test compounds and used reference drugs^84^.

## Supplementary Material

Supplemental Material
