# Supplementary material for: Synthesis, potential antitumor activity, cell cycle analysis, and multitarget mechanisms of novel hydrazones incorporating a 4-methylsulfonylbenzene scaffold: a molecular docking study
Source: J Enzyme Inhib Med Chem. 2021 Jul 15;36(1):1521–39. doi: 10.1080/14756366.2021.1924698 (PMC8288134; doi:10.1080/14756366.2021.1924698)
Supplement: Supplemental Material [file IENZ_A_1924698_SM5087.pdf]

Synthesis, potential antitumor activity, cell cycle analysis, and multitarget mechanisms of novel hydrazones incorporating a 4-methylsulfonylbenzene scaffold: A molecular docking study

Alaa A.-M. Abdel-Aziz,* Adel S. El-Azab, Nawaf A. AlSaif, Ahmad J. Obaidullah, Abdulrahman M. Al-Obaid, Ibrahim A. Al-Suwaidan

Department of Pharmaceutical Chemistry, College of Pharmacy, P.O. Box 2457, King Saud University, Riyadh 11451, Saudi Arabia

1. NMR spectrum
2. Materials and methods
2.1. Biological evaluation
2.1.1. *In vitro* antitumor screening
2.1.2. Apoptosis assay
2.1.3. Cell cycle analysis
2.1.4. *In vitro* cyclooxygenase (COX) inhibition assay
2.1.5. EGFR and Her2 assay
2.2. Molecular docking method
2.2.1. Selection of protein crystal structure
2.2.2. Preparation of the ligand
2.2.3. Docking experiment
Figure S1. NCI Dose-Response Curves for compound **20**

1. NMR spectrum

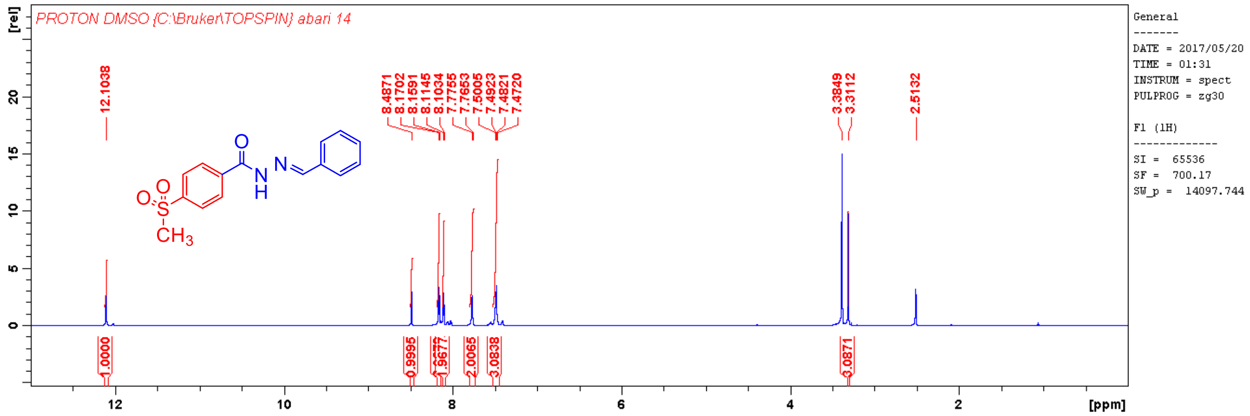

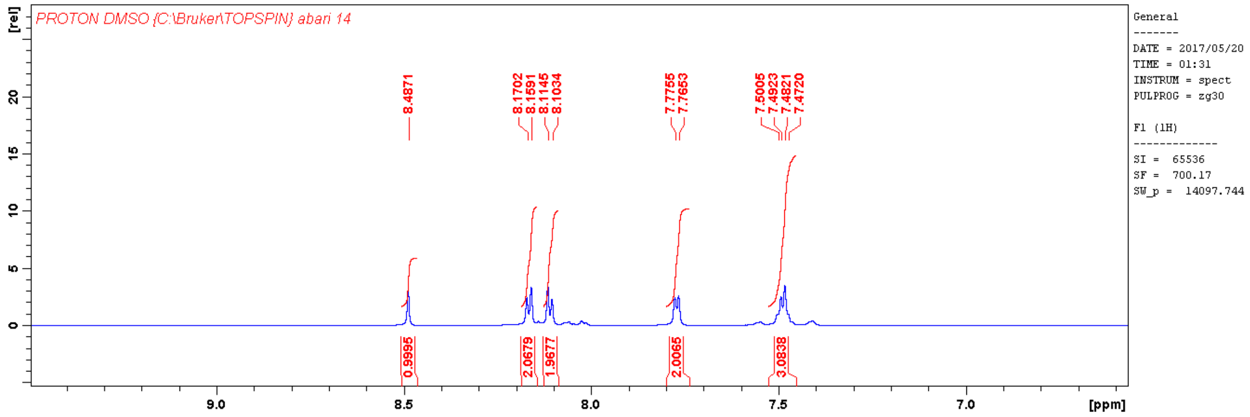

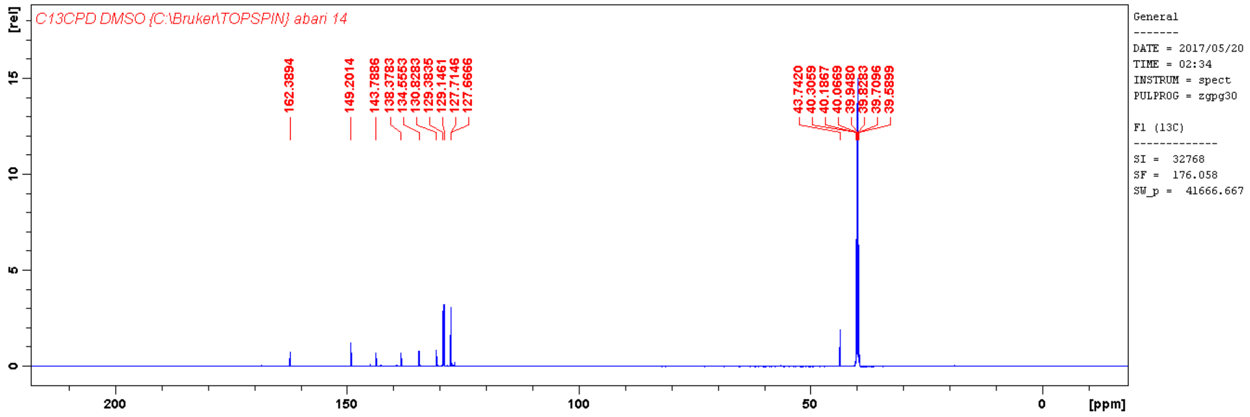

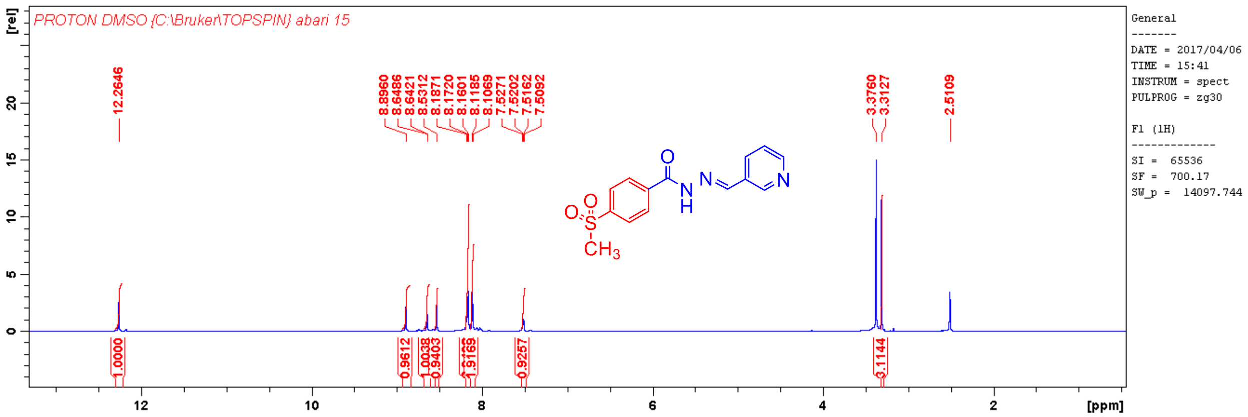
PROTON DMSO {C:\Bruker\TOPSPIN} abari 15
12.2646
8.8960
8.6486
8.6421
8.5312
8.1871
8.1720
8.1601
8.1185
8.1069
7.5271
7.5202
7.5162
7.5092
3.3760
3.3127
2.5109
1.0000
0.9612
1.0038
0.9403
2.9498
1.9169
0.9257
3.1144
[rel]
[ppm]
General
DATE = 2017/04/06
TIME = 15:41
INSTRUM = spect
PULPROG = zg30
F1 (1H)
SI = 65536
SF = 700.17
SW_p = 14097.744

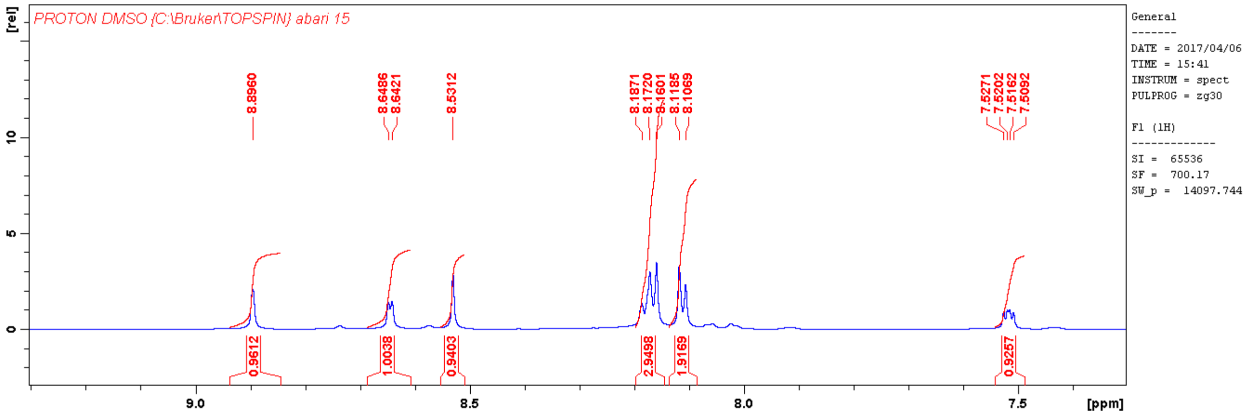
PROTON DMSO {C:\Bruker\TOPSPIN} abari 15
8.8960
8.6486
8.6421
8.5312
8.1871
8.1720
8.1601
8.1185
8.1069
7.5271
7.5202
7.5162
7.5092
0.9612
1.0038
0.9403
2.9498
1.9169
0.9257
[rel]
[ppm]
General
DATE = 2017/04/06
TIME = 15:41
INSTRUM = spect
PULPROG = zg30
F1 (1H)
SI = 65536
SF = 700.17
SW_p = 14097.744

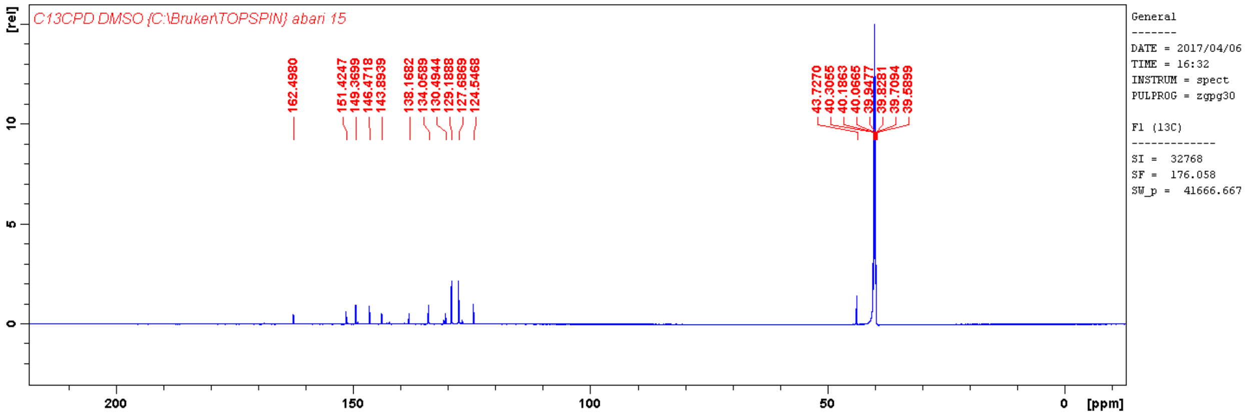
C13CPD DMSO {C:\Bruker\TOPSPIN} abari 15
162.4980
151.4247
149.3699
146.4718
143.8939
138.1682
134.0589
130.4944
129.1888
127.6869
124.5468
43.7270
40.3055
40.1863
40.0665
39.9477
39.8281
39.7094
39.5899
[rel]
[ppm]
General
DATE = 2017/04/06
TIME = 16:32
INSTRUM = spect
PULPROG = zgpg30
F1 (13C)
SI = 32768
SF = 176.058
SW_p = 41666.667

4

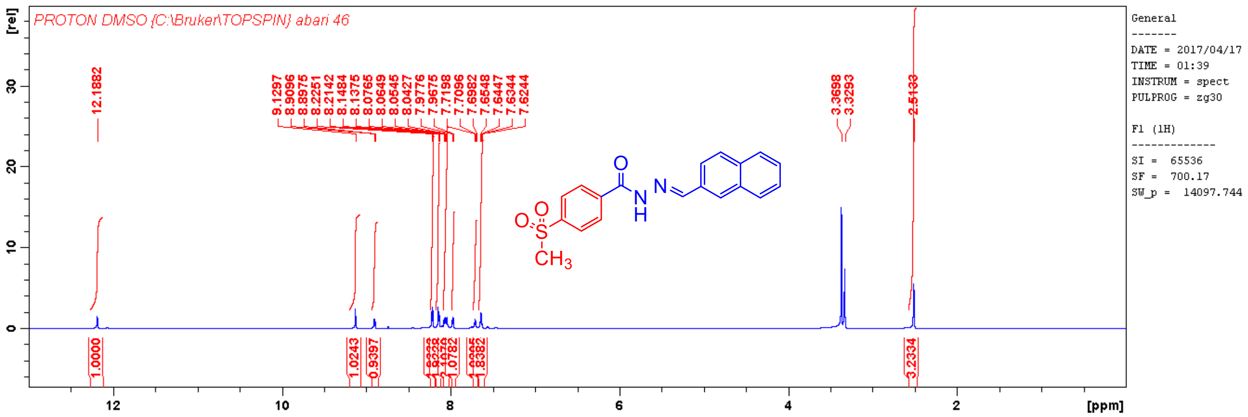
PROTON DMSO {C:\Bruker\TOPSPIN} abari 46
12.1882
9.1297 8.9096 8.8975 8.2251 8.2142 8.1484 8.1375 8.0765 8.0649 8.0545 8.0427 7.9776 7.9675 7.7198 7.7096 7.6982 7.6548 7.6447 7.6344 7.6244
3.3698 3.3293
2.5133
1.0000 1.0243 0.9397 1.8382 3.2334
[rel]
[ppm]
General

DATE = 2017/04/17
TIME = 01:39
INSTRUM = spect
PULPROG = zg30
F1 (1H)

SI = 65536
SF = 700.17
SW_p = 14097.744

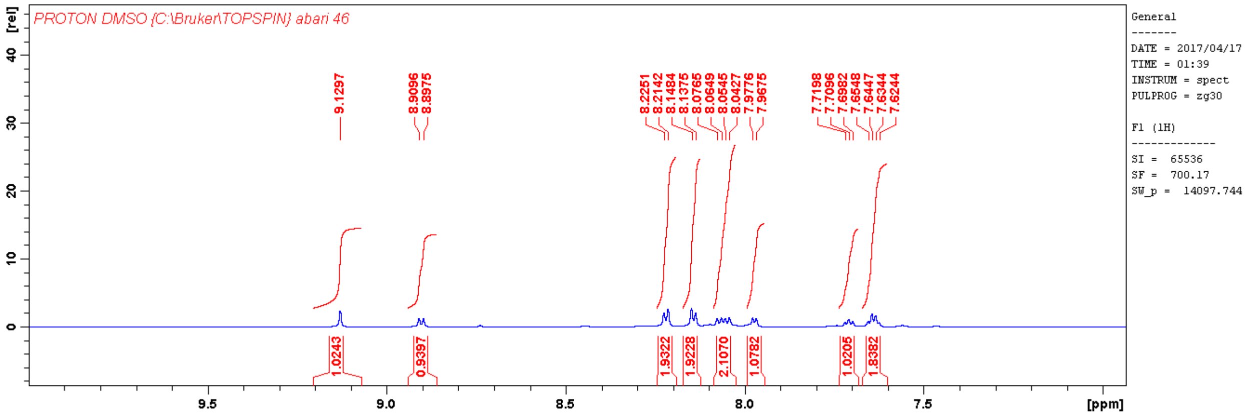
PROTON DMSO {C:\Bruker\TOPSPIN} abari 46
9.1297
8.9096 8.8975
8.2251 8.2142 8.1484 8.1375 8.0765 8.0649 8.0545 8.0427 7.9776 7.9675
7.7198 7.7096 7.6982 7.6548 7.6447 7.6344 7.6244
1.0243 0.9397 1.9322 1.9228 2.1070 1.0782 1.0205 1.8382
[rel]
[ppm]
General

DATE = 2017/04/17
TIME = 01:39
INSTRUM = spect
PULPROG = zg30
F1 (1H)

SI = 65536
SF = 700.17
SW_p = 14097.744

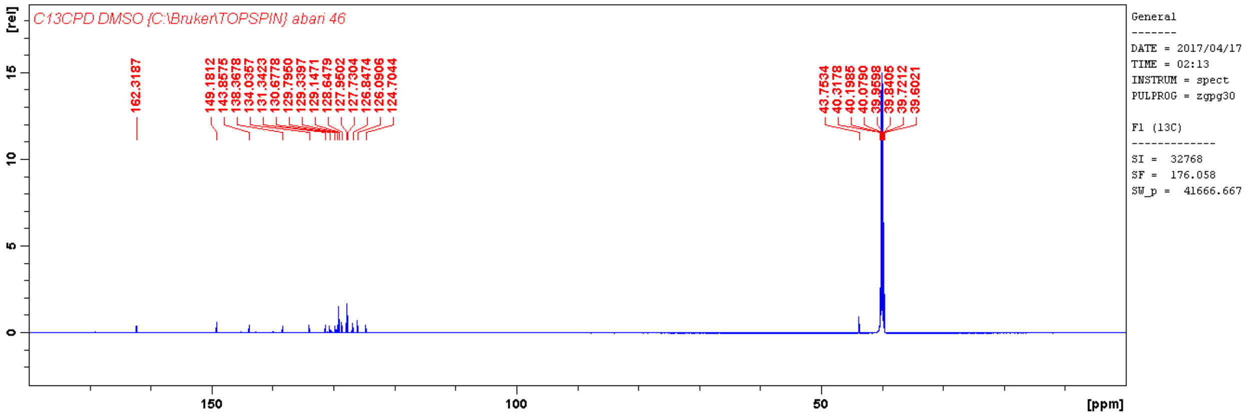
C13CPD DMSO {C:\Bruker\TOPSPIN} abari 46
162.3187
149.1812 143.8575 138.3678 134.0357 131.3423 130.6778 129.7950 129.3397 129.1471 128.6479 127.9502 127.7304 126.8474 126.0906 124.7044
43.7534 40.3178 40.1985 40.0790 39.9598 39.8405 39.7212 39.6021
[rel]
[ppm]
General

DATE = 2017/04/17
TIME = 02:13
INSTRUM = spect
PULPROG = zgpg30
F1 (13C)

SI = 32768
SF = 176.058
SW_p = 41666.667

5

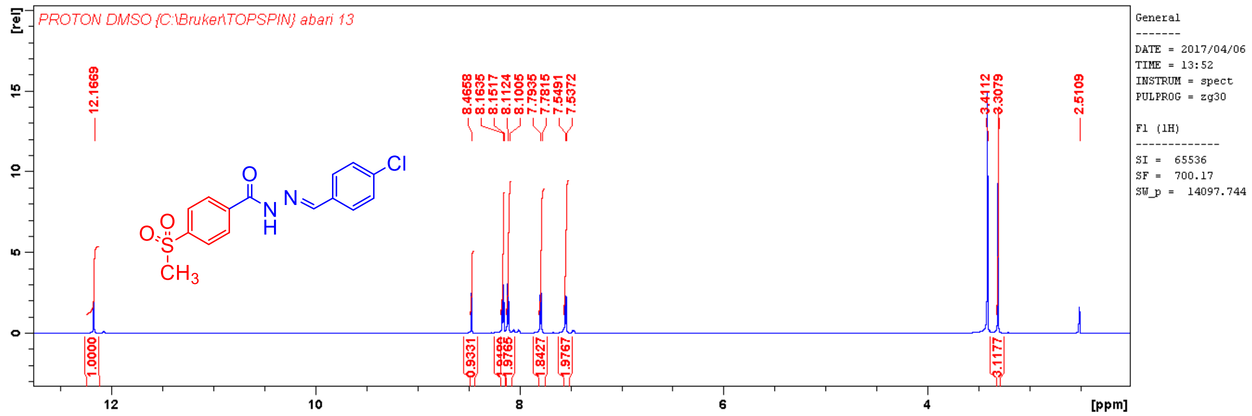

PROTON DMSO {C:\Bruker\TOPSPIN} abari 13
12.1669
8.4658
8.1635
8.1517
8.1124
8.1005
7.7935
7.7815
7.5491
7.5372
3.4112
3.3079
2.5109
1.0000
0.9331
1.9765
1.8427
1.9767
3.1177
[rel]
[ppm]
General
DATE = 2017/04/06
TIME = 13:52
INSTRUM = spect
PULPROG = zg30
F1 (1H)
SI = 65536
SF = 700.17
SW_p = 14097.744

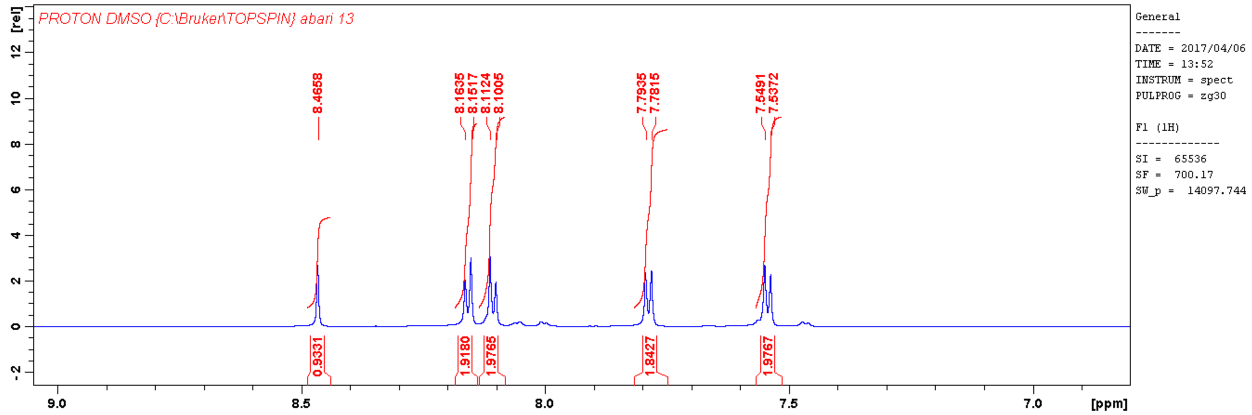

PROTON DMSO {C:\Bruker\TOPSPIN} abari 13
8.4658
8.1635
8.1517
8.1124
8.1005
7.7935
7.7815
7.5491
7.5372
0.9331
1.9180
1.9765
1.8427
1.9767
[rel]
[ppm]
General
DATE = 2017/04/06
TIME = 13:52
INSTRUM = spect
PULPROG = zg30
F1 (1H)
SI = 65536
SF = 700.17
SW_p = 14097.744

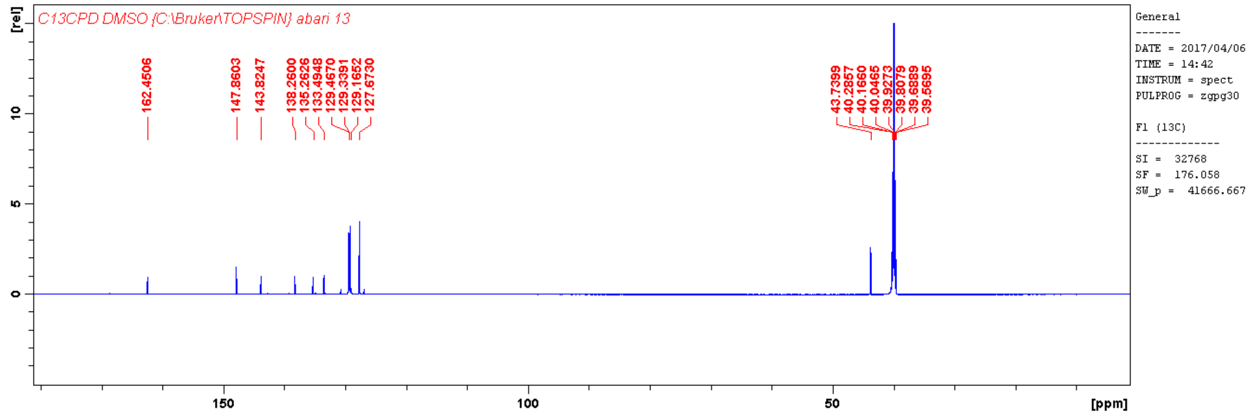

C13CPD DMSO {C:\Bruker\TOPSPIN} abari 13
162.4506
147.8603
143.8247
138.2600
135.2626
133.4948
129.4670
129.3391
129.1652
127.6730
43.7399
40.2857
40.1660
40.0465
39.9273
39.8079
39.6889
39.5695
[rel]
[ppm]
General
DATE = 2017/04/06
TIME = 14:42
INSTRUM = spect
PULPROG = zgpg30
F1 (13C)
SI = 32768
SF = 176.058
SW_p = 41666.667

6

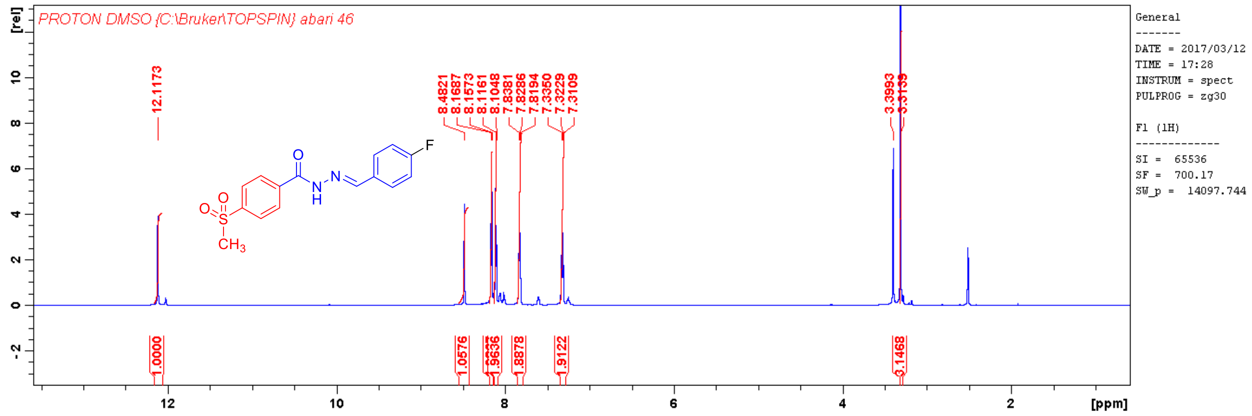
PROTON DMSO {C:\Bruker\TOPSPIN} abari 46
12.1173
8.4821 8.1687 8.1573 8.1161 8.1048 7.8381 7.8286 7.8194 7.3350 7.3229 7.3109
3.3993 3.3139
1.0000
1.0576 1.9636 1.8878 1.9122
3.1468
[ppm]
[rel]
General
DATE = 2017/03/12
TIME = 17:28
INSTRUM = spect
PULPROG = zg30
F1 (1H)
SI = 65536
SF = 700.17
SW_p = 14097.744

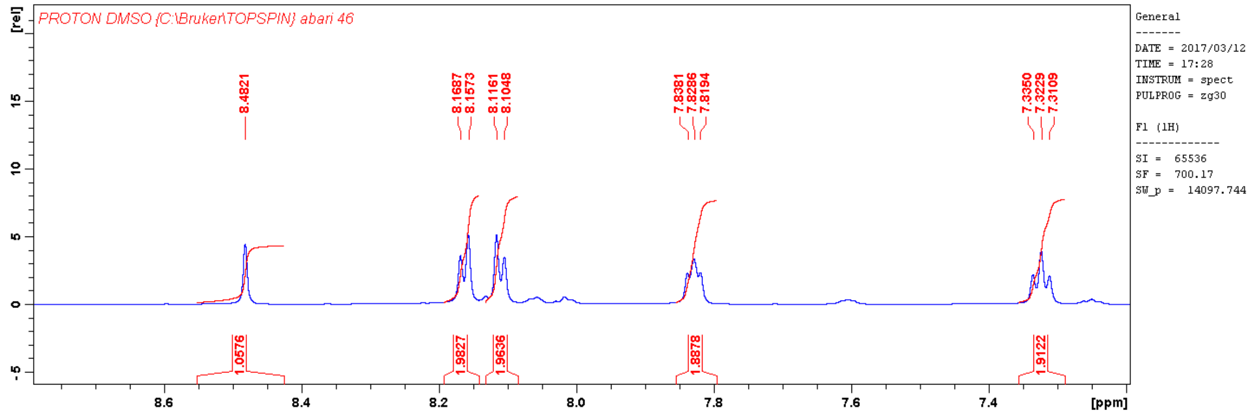
PROTON DMSO {C:\Bruker\TOPSPIN} abari 46
8.4821
8.1687 8.1573 8.1161 8.1048
7.8381 7.8286 7.8194
7.3350 7.3229 7.3109
1.0576 1.9827 1.9636 1.8878 1.9122
[ppm]
[rel]
General
DATE = 2017/03/12
TIME = 17:28
INSTRUM = spect
PULPROG = zg30
F1 (1H)
SI = 65536
SF = 700.17
SW_p = 14097.744

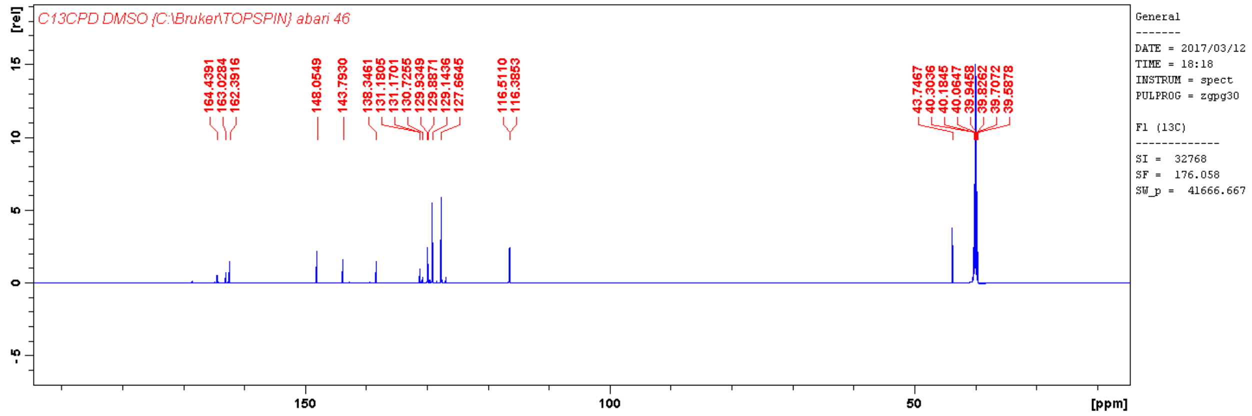
C13CPD DMSO {C:\Bruker\TOPSPIN} abari 46
164.4391 163.0284 162.3916
148.0549
143.7930
138.3461 131.1805 131.1701 130.7255 129.9349 129.8871 129.1436 127.6645
116.5110 116.3853
43.7467 40.3036 40.1845 40.0647 39.9458 39.8262 39.7072 39.5878
[ppm]
[rel]
General
DATE = 2017/03/12
TIME = 18:18
INSTRUM = spect
PULPROG = zgpg30
F1 (13C)
SI = 32768
SF = 176.058
SW_p = 41666.667

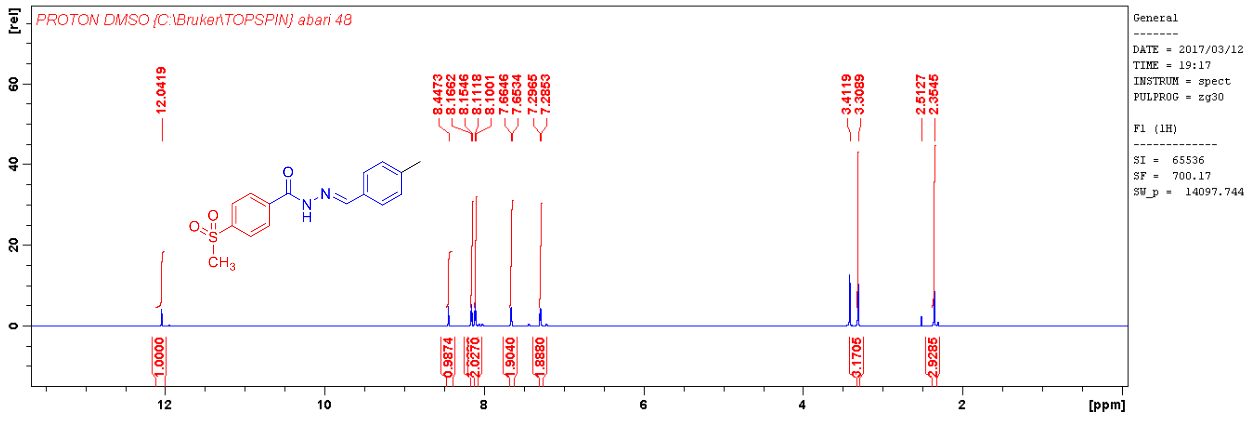
PROTON DMSO {C:\Bruker\TOPSPIN} abari 48
12.0419
8.4473
8.1662
8.1546
8.1118
8.1001
7.6646
7.6534
7.2965
7.2853
3.4119
3.3089
2.5127
2.3545
1.0000
0.9874
1.9296
2.0270
1.9040
1.8880
3.1705
2.9285
O
N
N
H
O
S
O
CH3
[rel]
[ppm]
General
DATE = 2017/03/12
TIME = 19:17
INSTRUM = spect
PULPROG = zg30
F1 (1H)
SI = 65536
SF = 700.17
SW_p = 14097.744

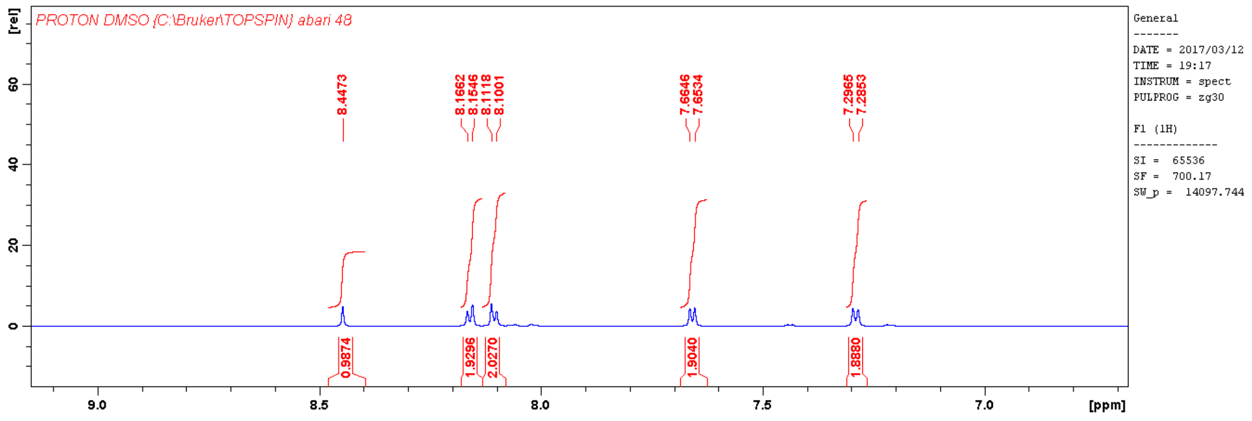
PROTON DMSO {C:\Bruker\TOPSPIN} abari 48
8.4473
8.1662
8.1546
8.1118
8.1001
7.6646
7.6534
7.2965
7.2853
0.9874
1.9296
2.0270
1.9040
1.8880
[rel]
[ppm]
General
DATE = 2017/03/12
TIME = 19:17
INSTRUM = spect
PULPROG = zg30
F1 (1H)
SI = 65536
SF = 700.17
SW_p = 14097.744

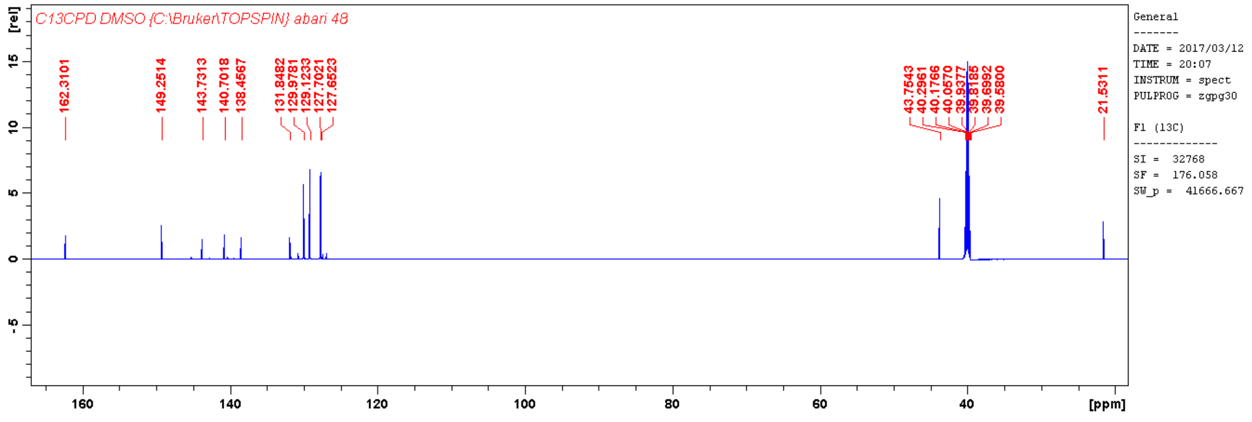
C13CPD DMSO {C:\Bruker\TOPSPIN} abari 48
162.3101
149.2514
143.7313
140.7018
138.4567
131.8482
129.9781
129.1233
127.7021
127.6523
43.7543
40.2961
40.1766
40.0570
39.9377
39.8185
39.6992
39.5800
21.5311
[rel]
[ppm]
General
DATE = 2017/03/12
TIME = 20:07
INSTRUM = spect
PULPROG = zgpg30
F1 (13C)
SI = 32768
SF = 176.058
SW_p = 41666.667

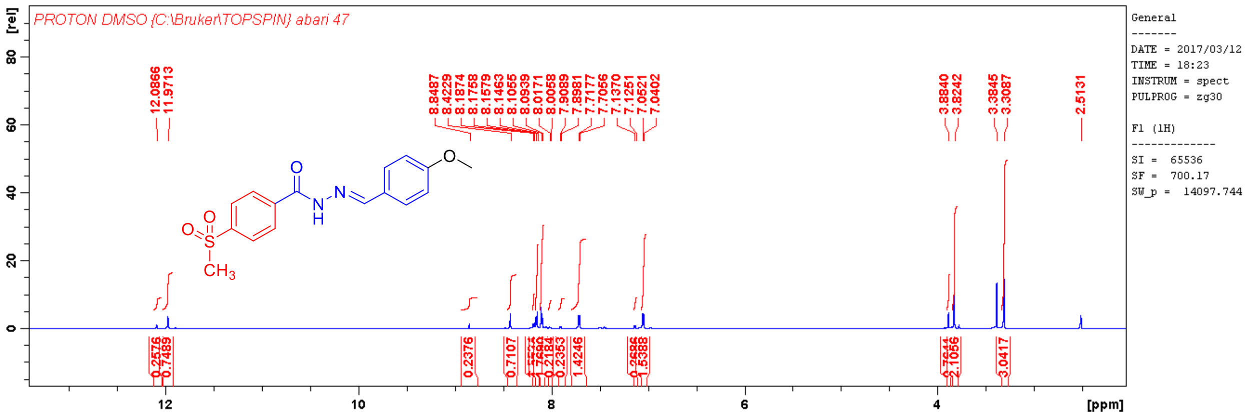
PROTON DMSO {C:\Bruker\TOPSPIN} abari 47
12.0866
11.9713
8.8487
8.4229
8.1874
8.1758
8.1579
8.1463
8.1055
8.0939
8.0171
8.0058
7.9089
7.8981
7.7177
7.7056
7.1370
7.1251
7.0521
7.0402
3.8840
3.8242
3.3845
3.3087
2.5131
O
N
H
N
O
O
S
CH3
0.2576
0.7489
0.2376
0.7107
2.1056
3.0417
1.4246
1.5388
[rel]
[ppm]
General
DATE = 2017/03/12
TIME = 18:23
INSTRUM = spect
PULPROG = zg30
F1 (1H)
SI = 65536
SF = 700.17
SW_p = 14097.744

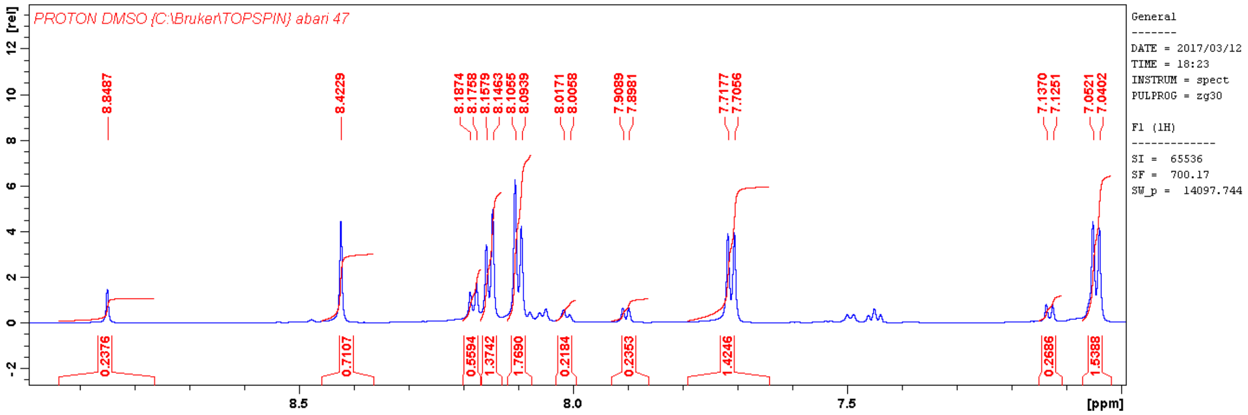
PROTON DMSO {C:\Bruker\TOPSPIN} abari 47
8.8487
8.4229
8.1874
8.1758
8.1579
8.1463
8.1055
8.0939
8.0171
8.0058
7.9089
7.8981
7.7177
7.7056
7.1370
7.1251
7.0521
7.0402
0.2376
0.7107
0.5594
1.3742
1.7690
0.2184
0.2353
1.4246
0.2686
1.5388
[rel]
[ppm]
General
DATE = 2017/03/12
TIME = 18:23
INSTRUM = spect
PULPROG = zg30
F1 (1H)
SI = 65536
SF = 700.17
SW_p = 14097.744

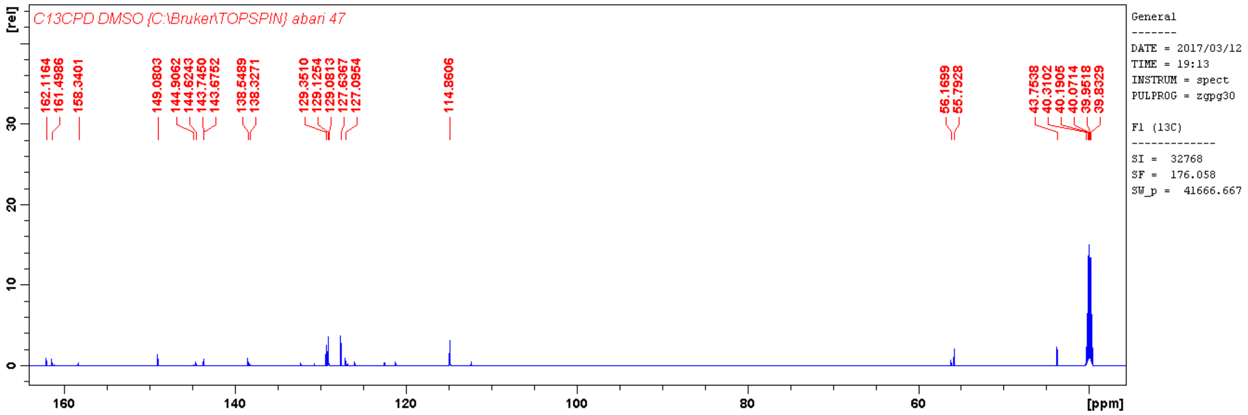
C13CPD DMSO {C:\Bruker\TOPSPIN} abari 47
162.1164
161.4986
158.3401
149.0803
144.9062
144.6243
143.7450
143.6752
138.5489
138.3271
129.3510
129.1254
129.0813
127.6367
127.0954
114.8606
56.1699
55.7928
43.7538
40.3102
40.1905
40.0714
39.9518
39.8329
[rel]
[ppm]
General
DATE = 2017/03/12
TIME = 19:13
INSTRUM = spect
PULPROG = zgpg30
F1 (13C)
SI = 32768
SF = 176.058
SW_p = 41666.667

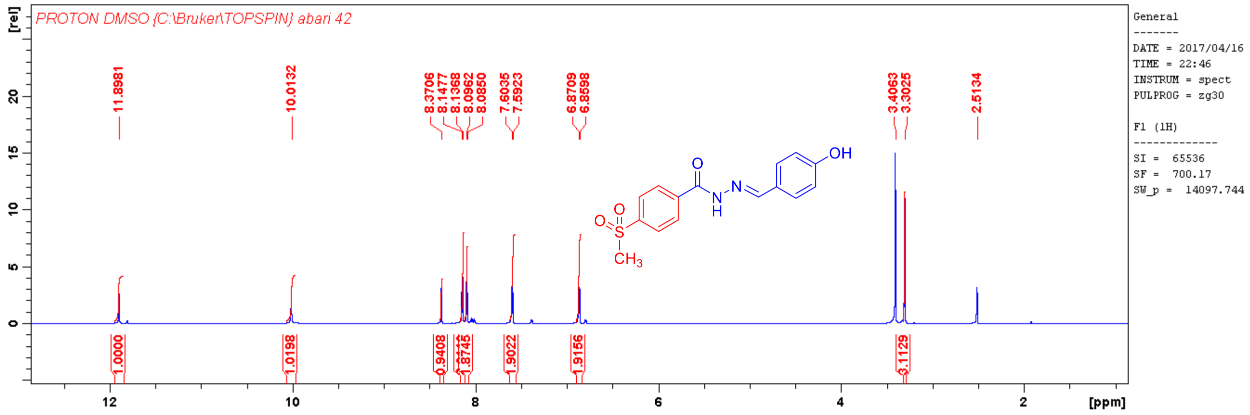
PROTON DMSO {C:\Bruker\TOPSPIN} abari 42
11.8981
10.0132
8.3706
8.1477
8.1368
8.0962
8.0850
7.6035
7.5923
6.8709
6.8598
3.4063
3.3025
2.5134
1.0000
1.0198
0.9408
2.0110
1.8745
1.9022
1.9156
3.1129
[rel]
[ppm]
OH
O
N
H
CH3
General
DATE = 2017/04/16
TIME = 22:46
INSTRUM = spect
PULPROG = zg30
F1 (1H)
SI = 65536
SF = 700.17
SW_p = 14097.744

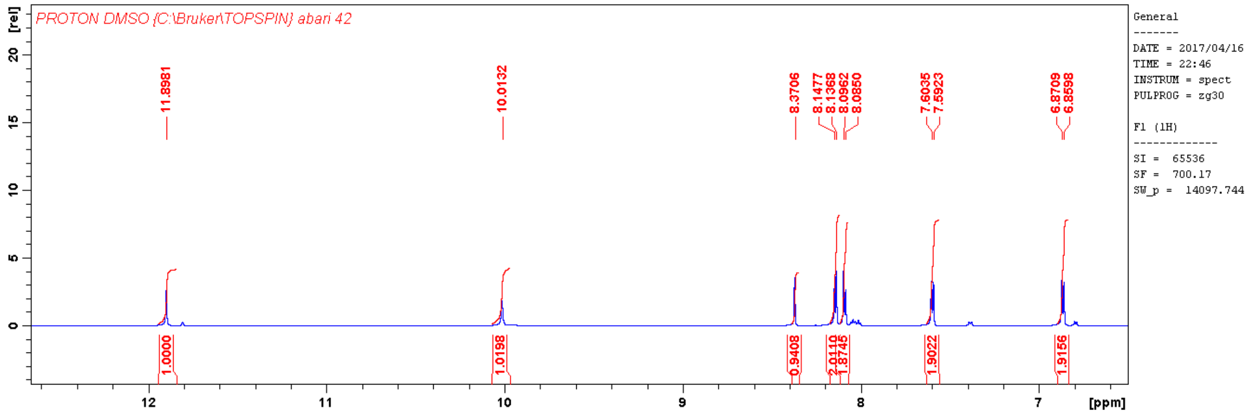
PROTON DMSO {C:\Bruker\TOPSPIN} abari 42
11.8981
10.0132
8.3706
8.1477
8.1368
8.0962
8.0850
7.6035
7.5923
6.8709
6.8598
1.0000
1.0198
0.9408
2.0110
1.8745
1.9022
1.9156
[rel]
[ppm]
General
DATE = 2017/04/16
TIME = 22:46
INSTRUM = spect
PULPROG = zg30
F1 (1H)
SI = 65536
SF = 700.17
SW_p = 14097.744

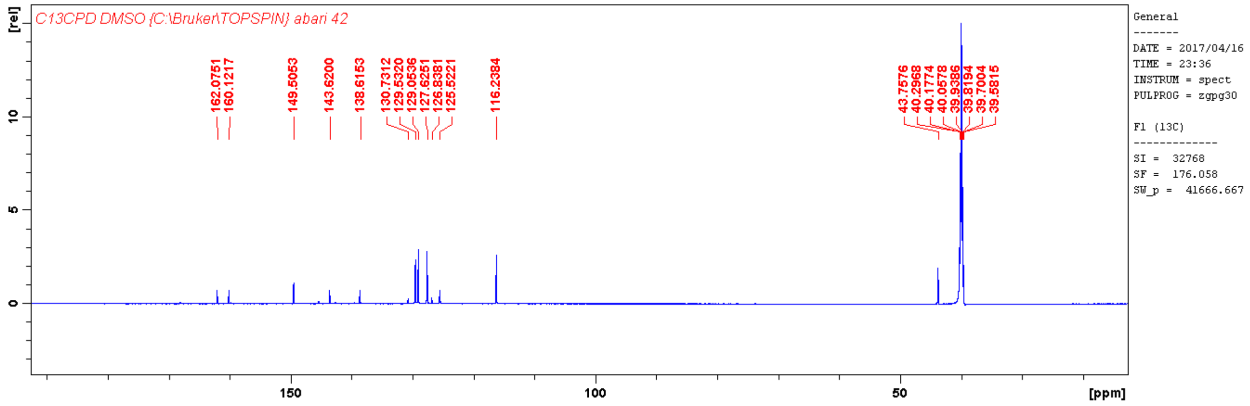
C13CPD DMSO {C:\Bruker\TOPSPIN} abari 42
162.0751
160.1217
149.5053
143.6200
138.6153
130.7312
129.5320
129.0536
127.6251
126.8381
125.5221
116.2384
43.7576
40.2968
40.1774
40.0578
39.9386
39.8194
39.7004
39.5815
[rel]
[ppm]
General
DATE = 2017/04/16
TIME = 23:36
INSTRUM = spect
PULPROG = zgpg30
F1 (13C)
SI = 32768
SF = 176.058
SW_p = 41666.667

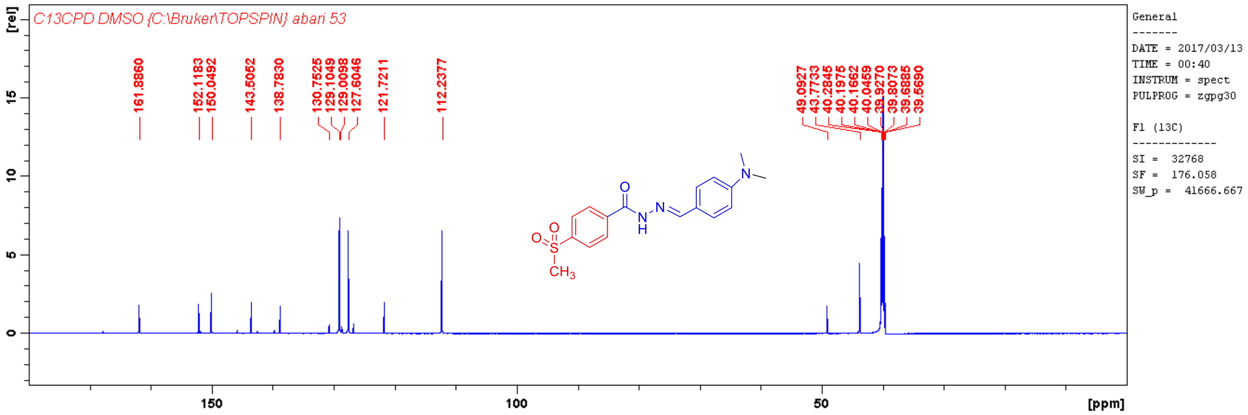

C13CPD DMSO {C:\Bruker\TOPSPIN} abari 53
161.8860
152.1183
150.0492
143.5052
138.7830
130.7525
129.1049
129.0098
127.6046
121.7211
112.2377
49.0927
43.7733
40.2845
40.1975
40.1662
40.0459
39.9270
39.8073
39.6885
39.5690
[rel]
[ppm]
General
DATE = 2017/03/13
TIME = 00:40
INSTRUM = spect
PULPROG = zgpg30
F1 (13C)
SI = 32768
SF = 176.058
SW_p = 41666.667

11

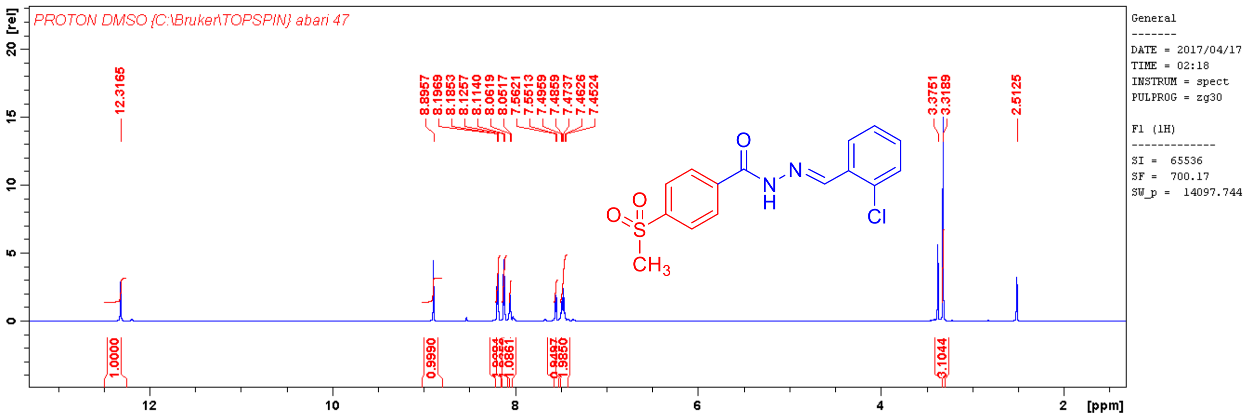
PROTON DMSO {C:\Bruker\TOPSPIN} abari 47
12.3165
8.8957 8.1969 8.1853 8.1257 8.1140 8.0619 8.0517 7.5621 7.5513 7.4959 7.4859 7.4737 7.4626 7.4524
3.3751 3.3189
2.5125
1.0000
0.9990
3.1044
Cl
O
N
N
H
O
O
S
CH3
[rel]
[ppm]
General
DATE = 2017/04/17
TIME = 02:18
INSTRUM = spect
PULPROG = zg30
F1 (1H)
SI = 65536
SF = 700.17
SW_p = 14097.744

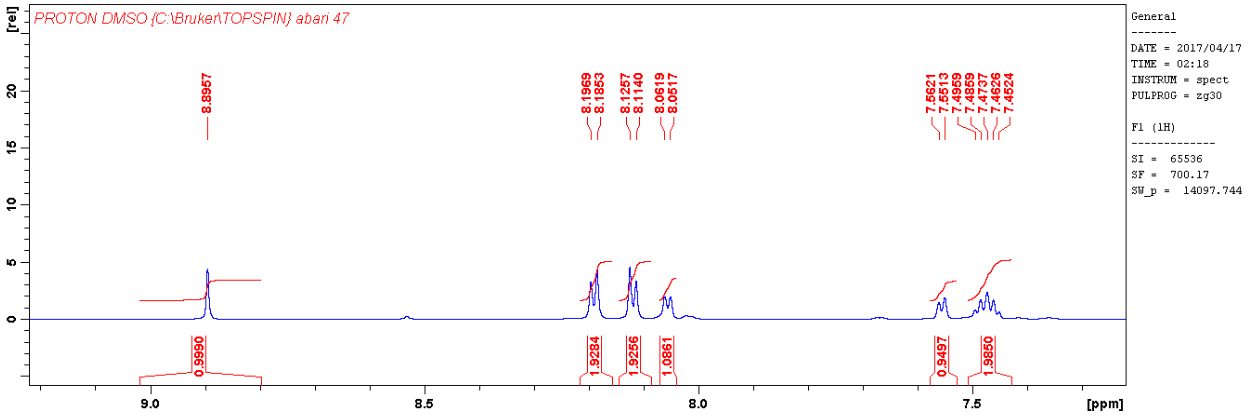
PROTON DMSO {C:\Bruker\TOPSPIN} abari 47
8.8957
8.1969 8.1853 8.1257 8.1140 8.0619 8.0517
7.5621 7.5513 7.4959 7.4859 7.4737 7.4626 7.4524
0.9990
1.9284
1.9256
1.0861
0.9497
1.9850
[rel]
[ppm]
General
DATE = 2017/04/17
TIME = 02:18
INSTRUM = spect
PULPROG = zg30
F1 (1H)
SI = 65536
SF = 700.17
SW_p = 14097.744

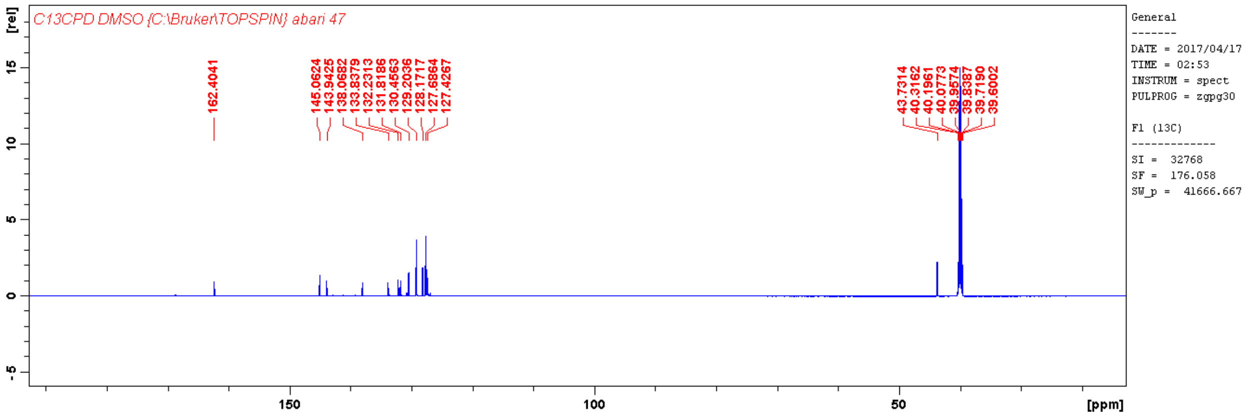
C13CPD DMSO {C:\Bruker\TOPSPIN} abari 47
162.4041
145.0624 143.9425 138.0682 133.8379 132.2313 131.8186 130.4563 129.2036 128.1717 127.6864 127.4267
43.7314 40.3162 40.1961 40.0773 39.9574 39.8387 39.7190 39.6002
[rel]
[ppm]
General
DATE = 2017/04/17
TIME = 02:53
INSTRUM = spect
PULPROG = zgpg30
F1 (13C)
SI = 32768
SF = 176.058
SW_p = 41666.667

12

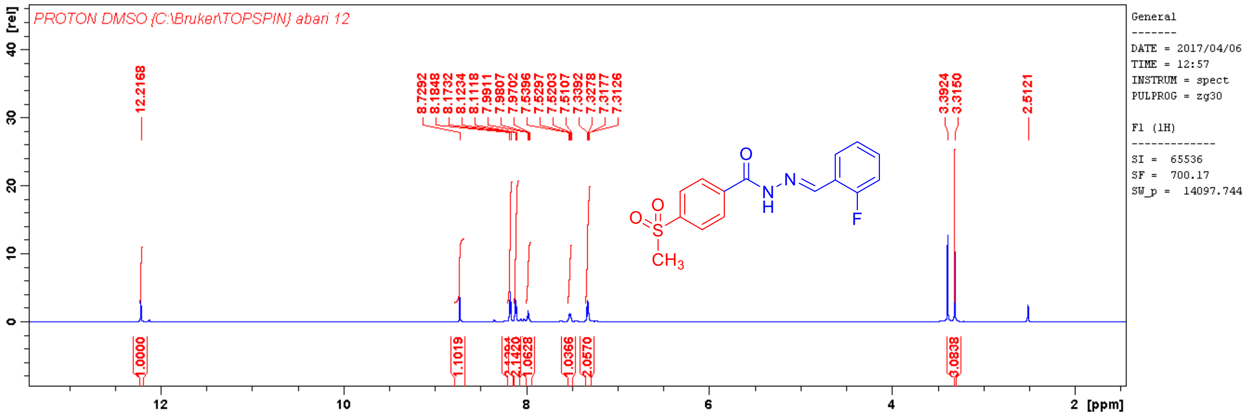
PROTON DMSO {C:\Bruker\TOPSPIN} abari 12
12.2168
8.7292 8.1848 8.1732 8.1234 8.1118 7.9911 7.9807 7.9702 7.5396 7.5297 7.5203 7.5107 7.3392 7.3278 7.3177 7.3126
3.3924 3.3150
2.5121
1.0000 1.1019 2.1394 2.1420 1.0628 1.0366 2.0570 3.0838
General
DATE = 2017/04/06
TIME = 12:57
INSTRUM = spect
PULPROG = zg30
F1 (1H)
SI = 65536
SF = 700.17
SW_p = 14097.744

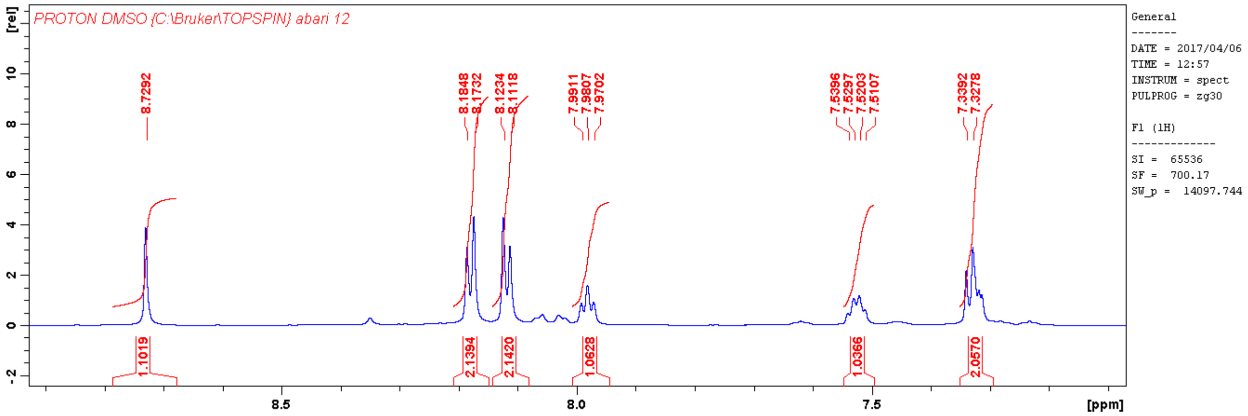
PROTON DMSO {C:\Bruker\TOPSPIN} abari 12
8.7292 8.1848 8.1732 8.1234 8.1118 7.9911 7.9807 7.9702 7.5396 7.5297 7.5203 7.5107 7.3392 7.3278
1.1019 2.1394 2.1420 1.0628 1.0366 2.0570
General
DATE = 2017/04/06
TIME = 12:57
INSTRUM = spect
PULPROG = zg30
F1 (1H)
SI = 65536
SF = 700.17
SW_p = 14097.744

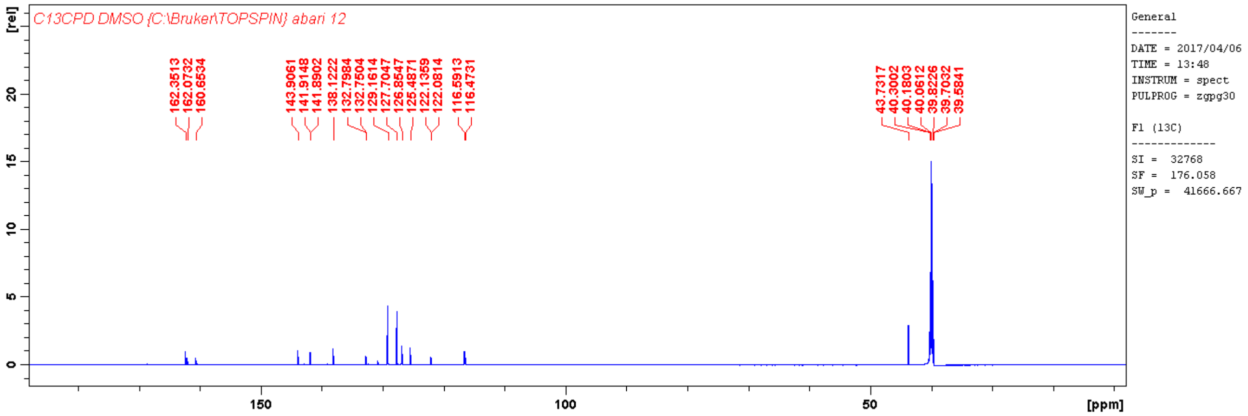
C13CPD DMSO {C:\Bruker\TOPSPIN} abari 12
162.3513 162.0732 160.6534
143.9061 141.9148 141.8902 138.1222 132.7984 132.7504 129.1614 127.7047 126.8547 125.4871 122.1359 122.0814 116.5913 116.4731
43.7317 40.3002 40.1803 40.0612 39.8226 39.7032 39.5841
General
DATE = 2017/04/06
TIME = 13:48
INSTRUM = spect
PULPROG = zgpg30
F1 (13C)
SI = 32768
SF = 176.058
SW_p = 41666.667

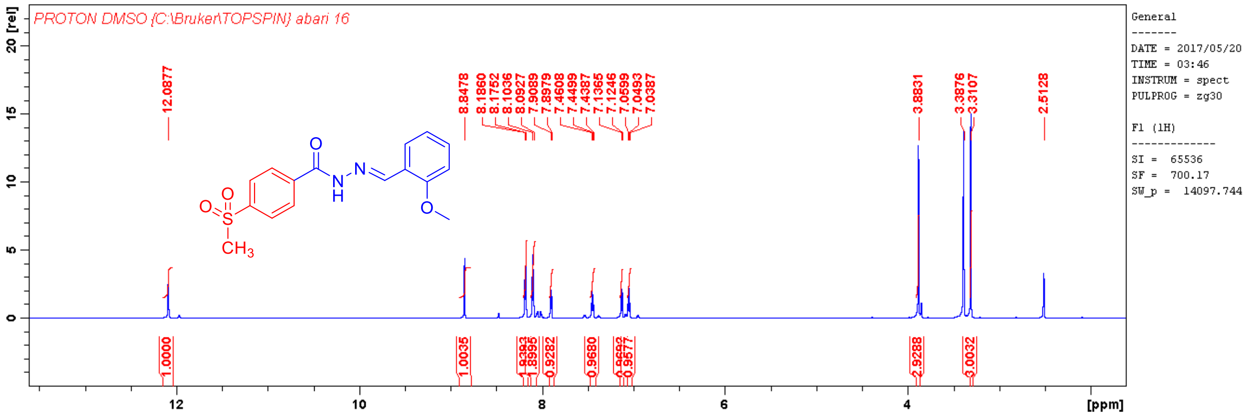
PROTON DMSO {C:\Bruker\TOPSPIN} abari 16
General
DATE = 2017/05/20
TIME = 03:46
INSTRUM = spect
PULPROG = zg30
F1 (1H)
SI = 65536
SF = 700.17
SW_p = 14097.744
[ppm]
[rel]

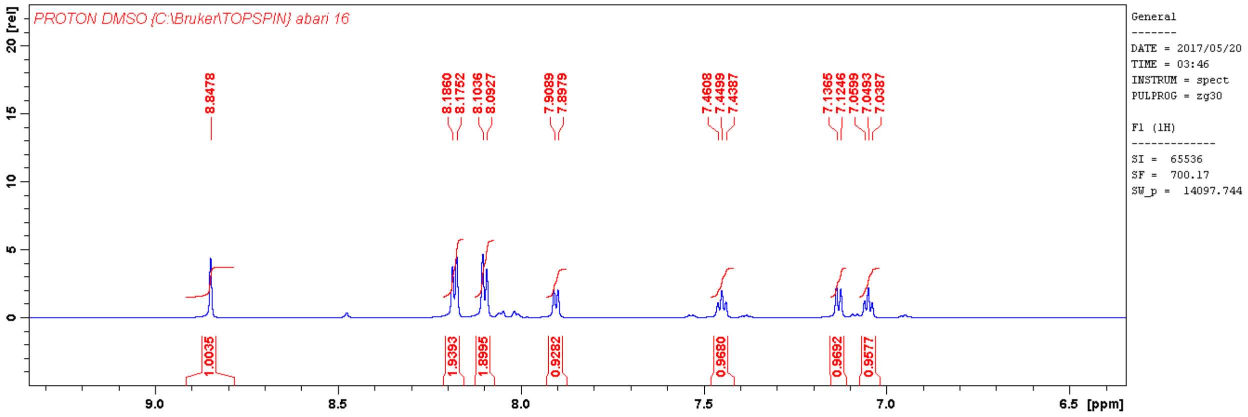
PROTON DMSO {C:\Bruker\TOPSPIN} abari 16
General
DATE = 2017/05/20
TIME = 03:46
INSTRUM = spect
PULPROG = zg30
F1 (1H)
SI = 65536
SF = 700.17
SW_p = 14097.744
[ppm]
[rel]

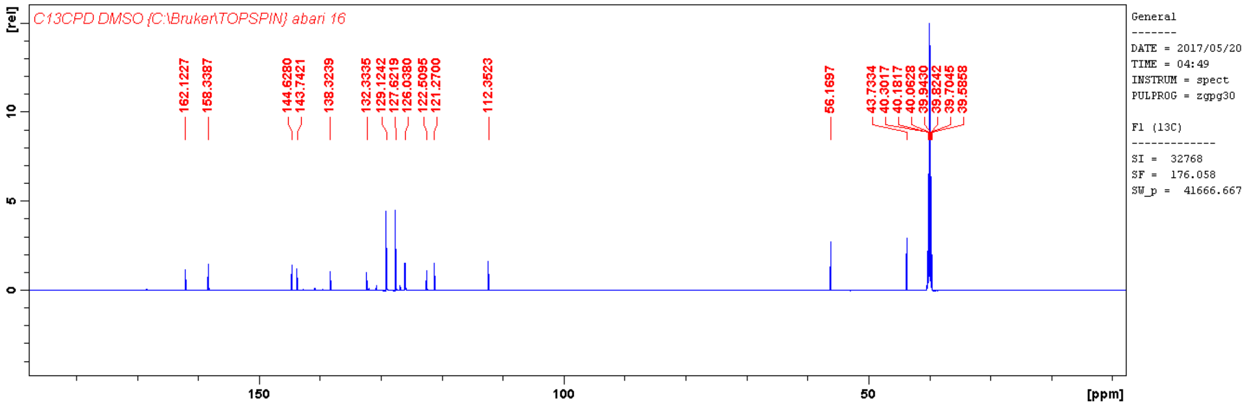
C13CPD DMSO {C:\Bruker\TOPSPIN} abari 16
General
DATE = 2017/05/20
TIME = 04:49
INSTRUM = spect
PULPROG = zgpg30
F1 (13C)
SI = 32768
SF = 176.058
SW_p = 41666.667
[ppm]
[rel]

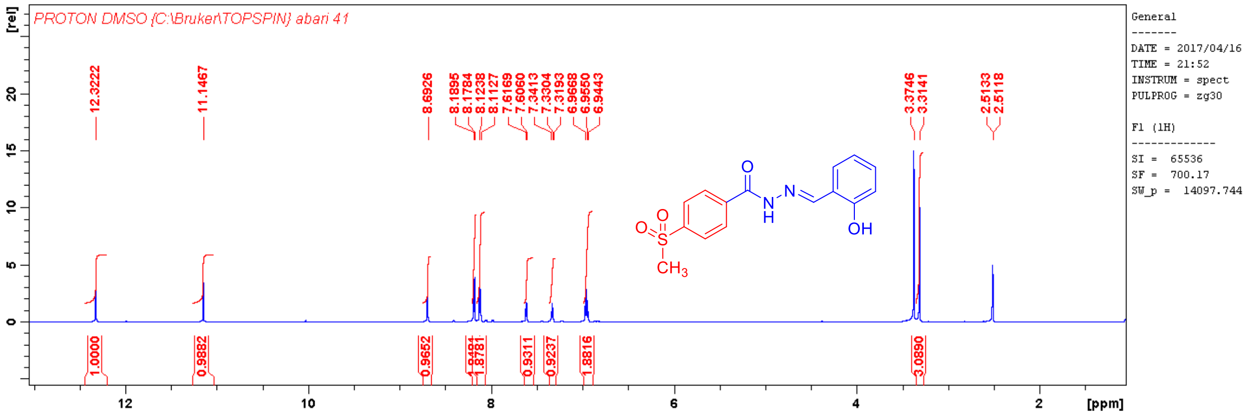
PROTON DMSO {C:\Bruker\TOPSPIN} abari 41
General
DATE = 2017/04/16
TIME = 21:52
INSTRUM = spect
PULPROG = zg30
F1 (1H)
SI = 65536
SF = 700.17
SW_p = 14097.744
12.3222
11.1467
8.6926
8.1895
8.1784
8.1238
8.1127
7.6169
7.6060
7.3413
7.3304
7.3193
6.9668
6.9550
6.9443
3.3746
3.3141
2.5133
2.5118
1.0000
0.9882
0.9652
1.8481
1.8781
0.9311
0.9237
1.8816
3.0890
OH
CH3
[ppm]

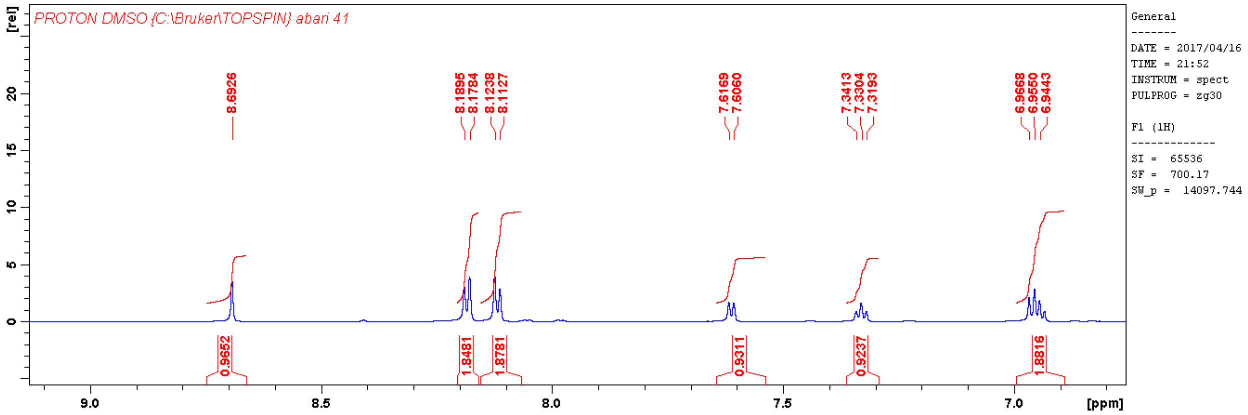
PROTON DMSO {C:\Bruker\TOPSPIN} abari 41
General
DATE = 2017/04/16
TIME = 21:52
INSTRUM = spect
PULPROG = zg30
F1 (1H)
SI = 65536
SF = 700.17
SW_p = 14097.744
8.6926
8.1895
8.1784
8.1238
8.1127
7.6169
7.6060
7.3413
7.3304
7.3193
6.9668
6.9550
6.9443
0.9652
1.8481
1.8781
0.9311
0.9237
1.8816
[ppm]

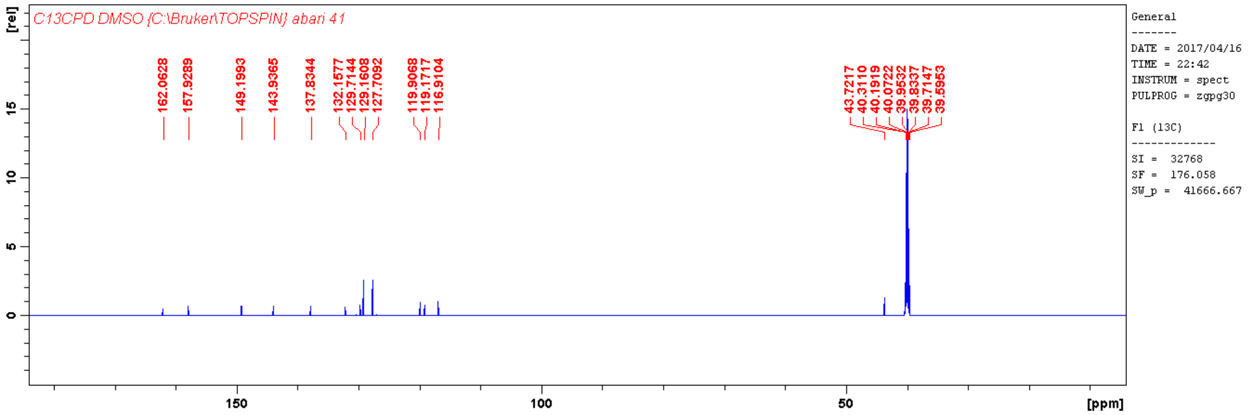
C13CPD DMSO {C:\Bruker\TOPSPIN} abari 41
General
DATE = 2017/04/16
TIME = 22:42
INSTRUM = spect
PULPROG = zgpg30
F1 (13C)
SI = 32768
SF = 176.058
SW_p = 41666.667
162.0628
157.9289
149.1993
143.9365
137.8344
132.1577
129.7144
129.1608
127.7092
119.9068
119.1717
116.9104
43.7217
40.3110
40.1919
40.0722
39.9532
39.8337
39.7147
39.5953
[ppm]

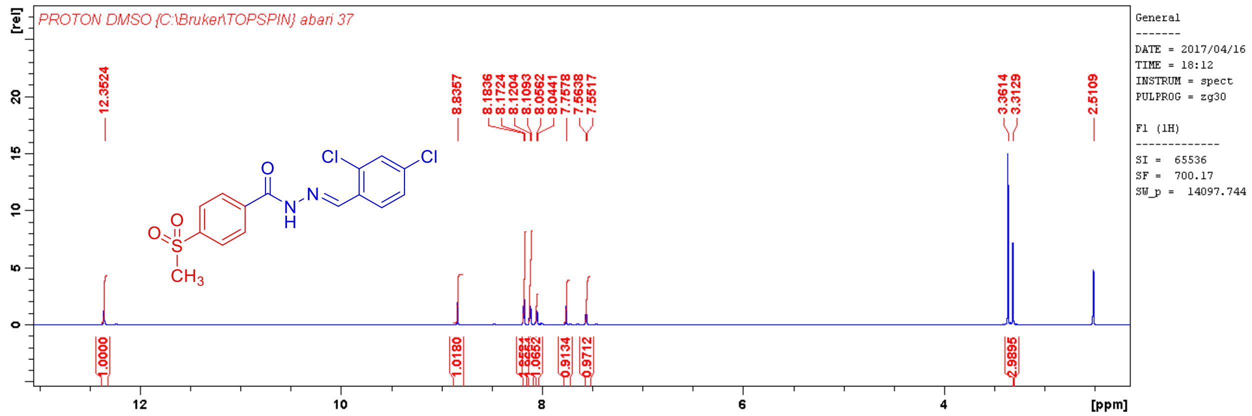
PROTON DMSO {C:\Bruker\TOPSPIN} abari 37
12.3524
8.8357
8.1836
8.1724
8.1204
8.1093
8.0562
8.0441
7.7578
7.5638
7.5517
3.3614
3.3129
2.5109
1.0000
1.0180
0.9134
0.9712
2.9895
[rel]
[ppm]
General
DATE = 2017/04/16
TIME = 18:12
INSTRUM = spect
PULPROG = zg30
F1 (1H)
SI = 65536
SF = 700.17
SW_p = 14097.744

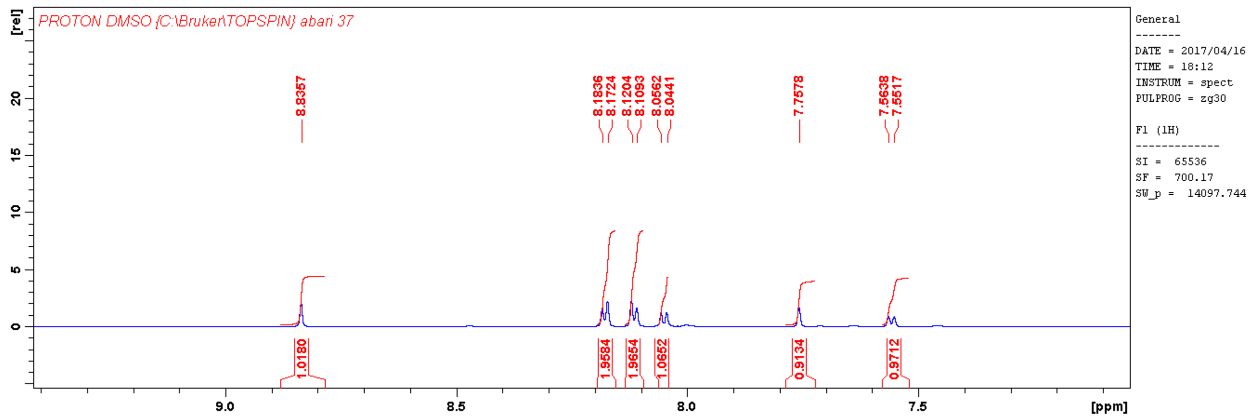
PROTON DMSO {C:\Bruker\TOPSPIN} abari 37
8.8357
8.1836
8.1724
8.1204
8.1093
8.0562
8.0441
7.7578
7.5638
7.5517
1.0180
1.9584
1.9654
1.0652
0.9134
0.9712
[rel]
[ppm]
General
DATE = 2017/04/16
TIME = 18:12
INSTRUM = spect
PULPROG = zg30
F1 (1H)
SI = 65536
SF = 700.17
SW_p = 14097.744

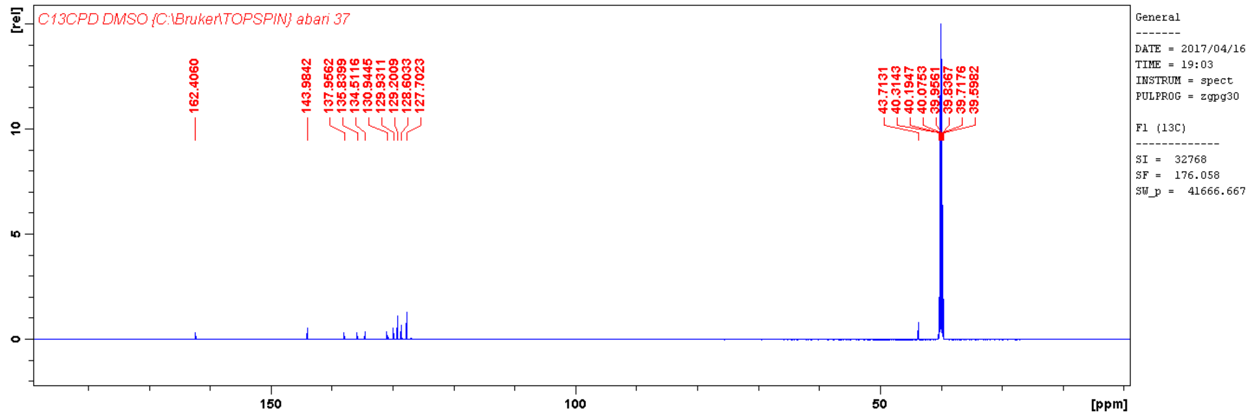
C13CPD DMSO {C:\Bruker\TOPSPIN} abari 37
162.4060
143.9842
137.9562
135.8399
134.5116
130.9445
129.9311
129.2009
128.6033
127.7023
43.7131
40.3143
40.1947
40.0753
39.9561
39.8367
39.7176
39.5982
[rel]
[ppm]
General
DATE = 2017/04/16
TIME = 19:03
INSTRUM = spect
PULPROG = zgpg30
F1 (13C)
SI = 32768
SF = 176.058
SW_p = 41666.667

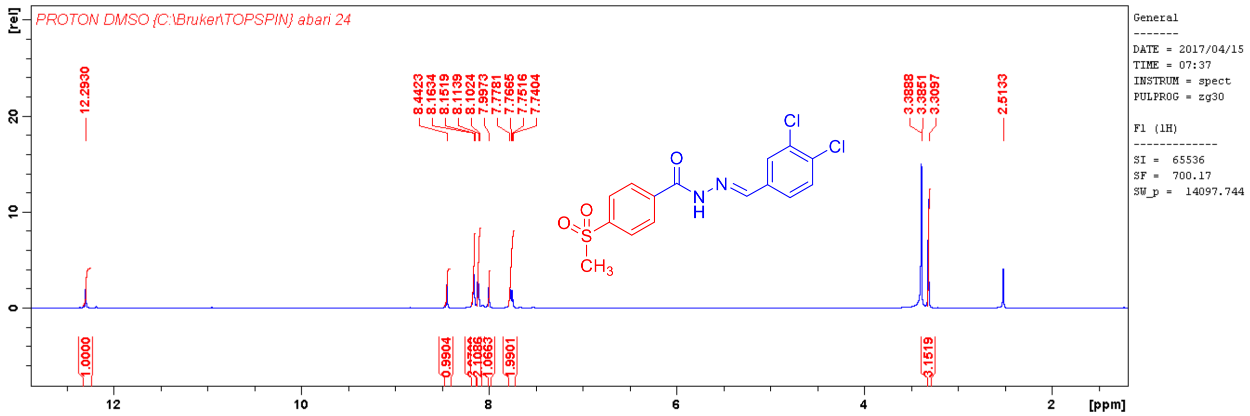
PROTON DMSO {C:\Bruker\TOPSPIN} abari 24
12.2930
8.4423
8.1634
8.1519
8.1139
8.1024
7.9973
7.7781
7.7665
7.7516
7.7404
3.3888
3.3851
3.3097
2.5133
1.0000
0.9904
2.0700
2.1086
1.0663
1.9901
3.1519
[ppm]
[rel]
General
DATE = 2017/04/15
TIME = 07:37
INSTRUM = spect
PULPROG = zg30
F1 (1H)
SI = 65536
SF = 700.17
SW_p = 14097.744
CH3
Cl
Cl
O
N
N
H
S
O
O

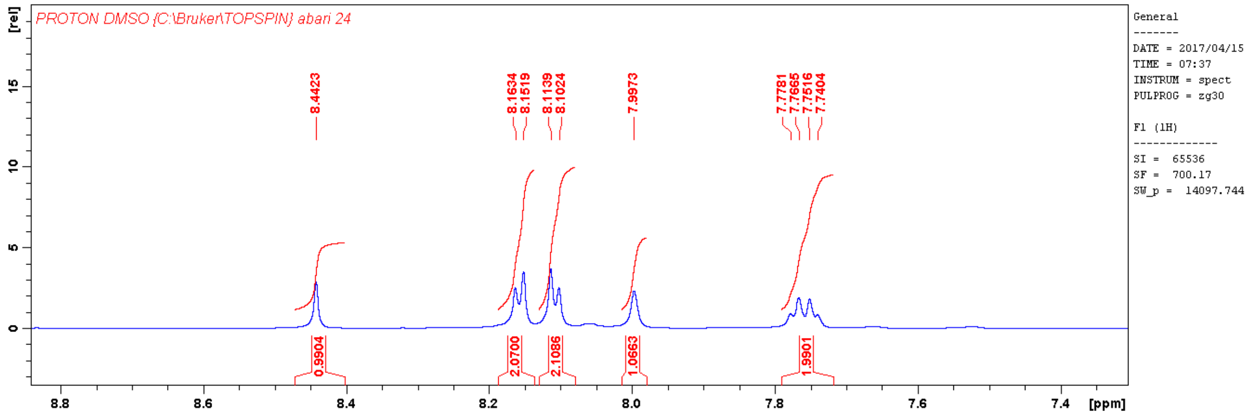
PROTON DMSO {C:\Bruker\TOPSPIN} abari 24
8.4423
8.1634
8.1519
8.1139
8.1024
7.9973
7.7781
7.7665
7.7516
7.7404
0.9904
2.0700
2.1086
1.0663
1.9901
[ppm]
[rel]
General
DATE = 2017/04/15
TIME = 07:37
INSTRUM = spect
PULPROG = zg30
F1 (1H)
SI = 65536
SF = 700.17
SW_p = 14097.744

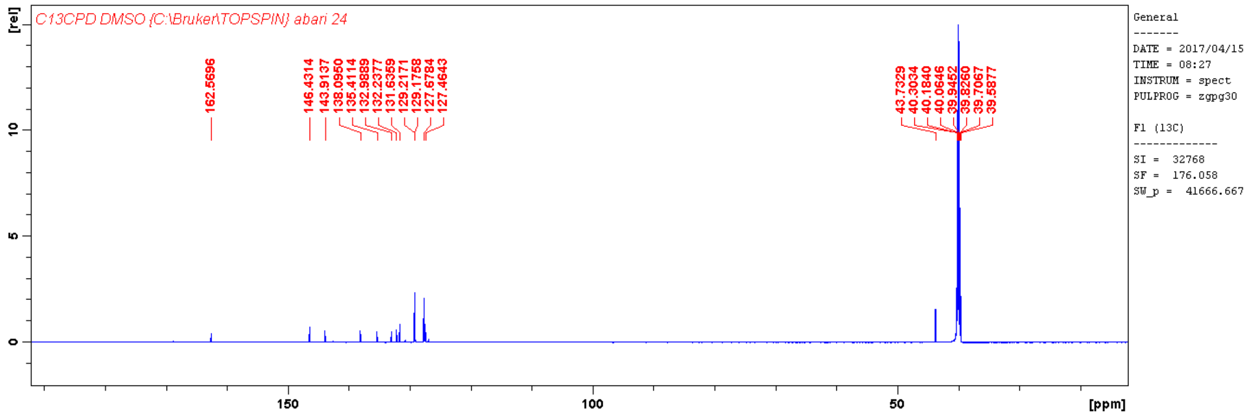
C13CPD DMSO {C:\Bruker\TOPSPIN} abari 24
162.5696
146.4314
143.9137
138.0950
135.4114
132.9889
132.2377
131.6359
129.2171
129.1758
127.6784
127.4643
43.7329
40.3034
40.1840
40.0646
39.9452
39.8260
39.7067
39.5877
[ppm]
[rel]
General
DATE = 2017/04/15
TIME = 08:27
INSTRUM = spect
PULPROG = zgpg30
F1 (13C)
SI = 32768
SF = 176.058
SW_p = 41666.667

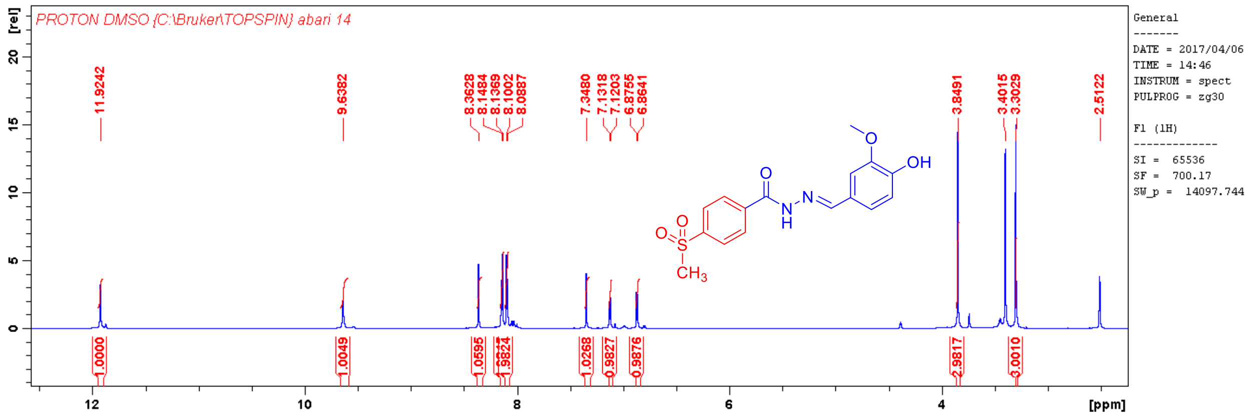
PROTON DMSO {C:\Bruker\TOPSPIN} abari 14
11.9242
9.6382
8.3628
8.1484
8.1369
8.1002
8.0887
7.3480
7.1318
7.1203
6.8755
6.8641
3.8491
3.4015
3.3029
2.5122
1.0000
1.0049
1.0595
1.9824
1.0268
0.9827
0.9876
2.9817
3.0010
[ppm]
[rel]
O
N
H
N
OH
O
O
S
O
CH3
General
DATE = 2017/04/06
TIME = 14:46
INSTRUM = spect
PULPROG = zg30
F1 (1H)
SI = 65536
SF = 700.17
SW_p = 14097.744

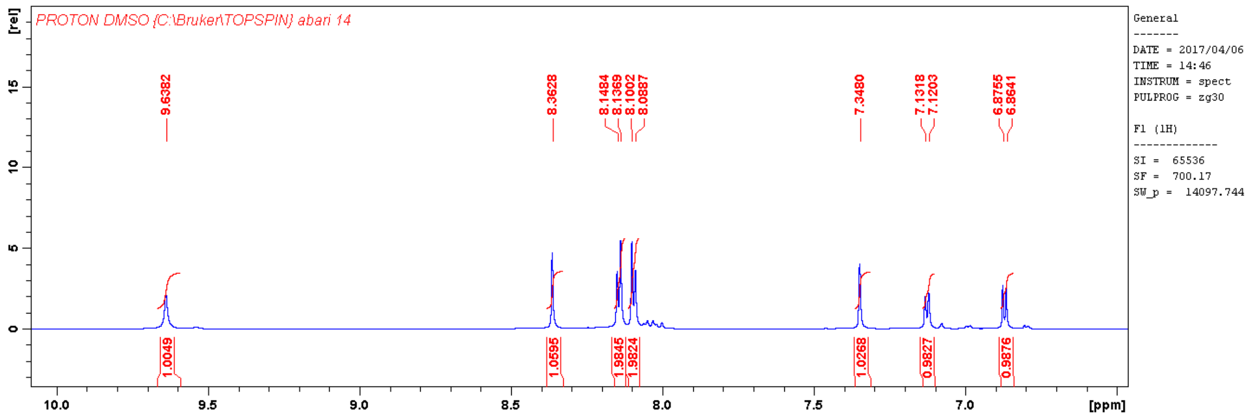
PROTON DMSO {C:\Bruker\TOPSPIN} abari 14
9.6382
8.3628
8.1484
8.1369
8.1002
8.0887
7.3480
7.1318
7.1203
6.8755
6.8641
1.0049
1.0595
1.9845
1.9824
1.0268
0.9827
0.9876
[ppm]
[rel]
General
DATE = 2017/04/06
TIME = 14:46
INSTRUM = spect
PULPROG = zg30
F1 (1H)
SI = 65536
SF = 700.17
SW_p = 14097.744

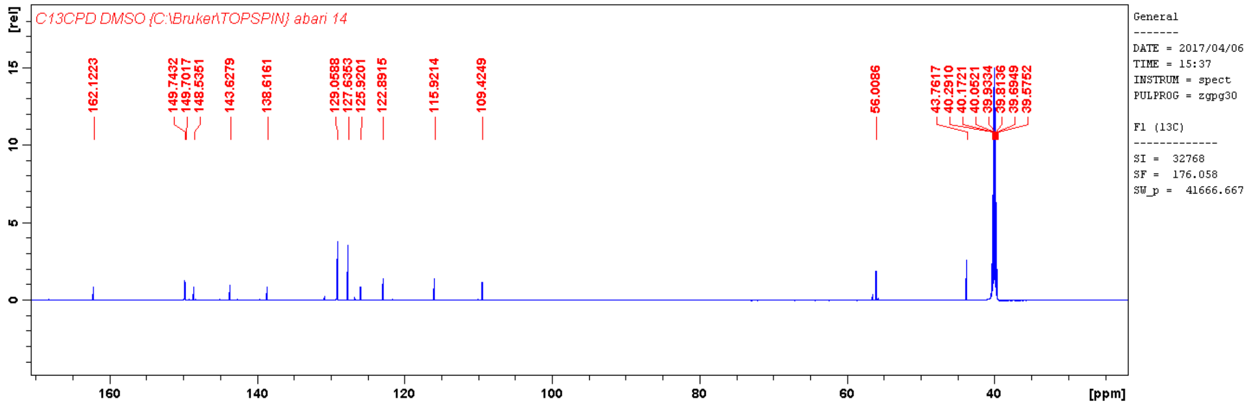
C13CPD DMSO {C:\Bruker\TOPSPIN} abari 14
162.1223
149.7432
149.7017
148.5351
143.6279
138.6161
129.0588
127.6353
125.9201
122.8915
115.9214
109.4249
56.0086
43.7617
40.2910
40.1721
40.0521
39.9334
39.8136
39.6949
39.5752
[ppm]
[rel]
General
DATE = 2017/04/06
TIME = 15:37
INSTRUM = spect
PULPROG = zgpg30
F1 (13C)
SI = 32768
SF = 176.058
SW_p = 41666.667

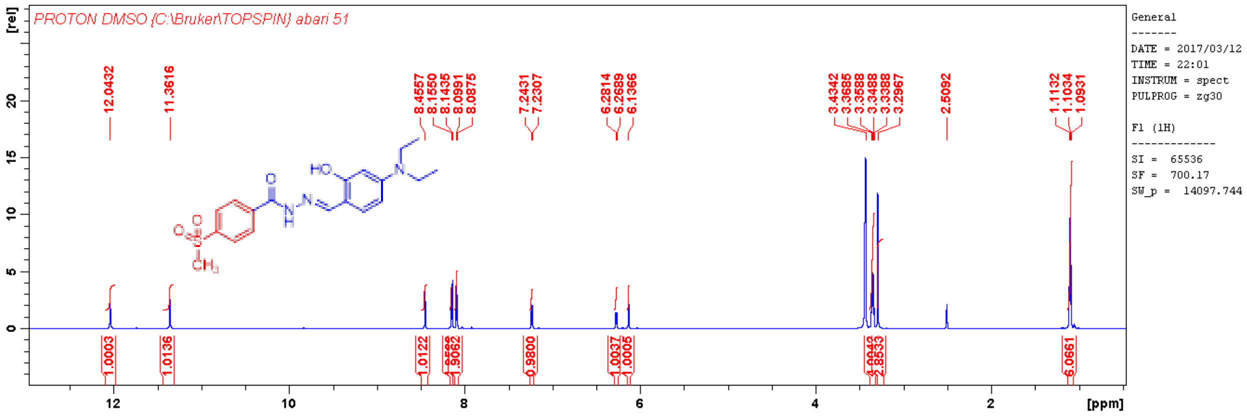
PROTON DMSO {C:\Bruker\TOPSPIN} abari 51
12.0432
11.3616
8.4557
8.1550
8.1435
8.0991
8.0875
7.2431
7.2307
6.2814
6.2689
6.1366
3.4342
3.3685
3.3588
3.3488
3.3388
3.2967
2.5092
1.1132
1.1034
1.0931
1.0003
1.0136
1.0122
1.9566
1.9062
0.9800
1.0037
1.0005
4.0042
2.8533
6.0661
HO
O
N
H
CH3
[ppm]
General
DATE = 2017/03/12
TIME = 22:01
INSTRUM = spect
PULPROG = zg30
F1 (1H)
SI = 65536
SF = 700.17
SW_p = 14097.744

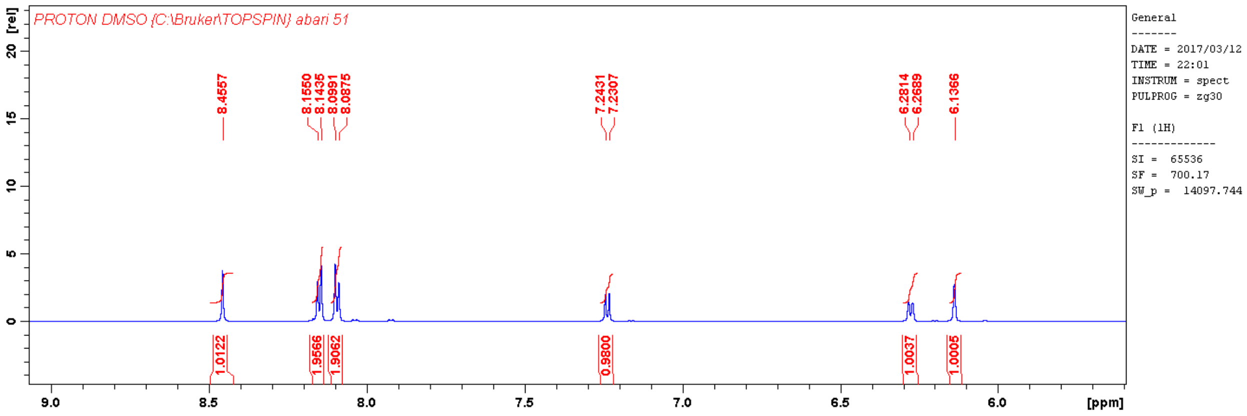
PROTON DMSO {C:\Bruker\TOPSPIN} abari 51
8.4557
8.1550
8.1435
8.0991
8.0875
7.2431
7.2307
6.2814
6.2689
6.1366
1.0122
1.9566
1.9062
0.9800
1.0037
1.0005
[ppm]
General
DATE = 2017/03/12
TIME = 22:01
INSTRUM = spect
PULPROG = zg30
F1 (1H)
SI = 65536
SF = 700.17
SW_p = 14097.744

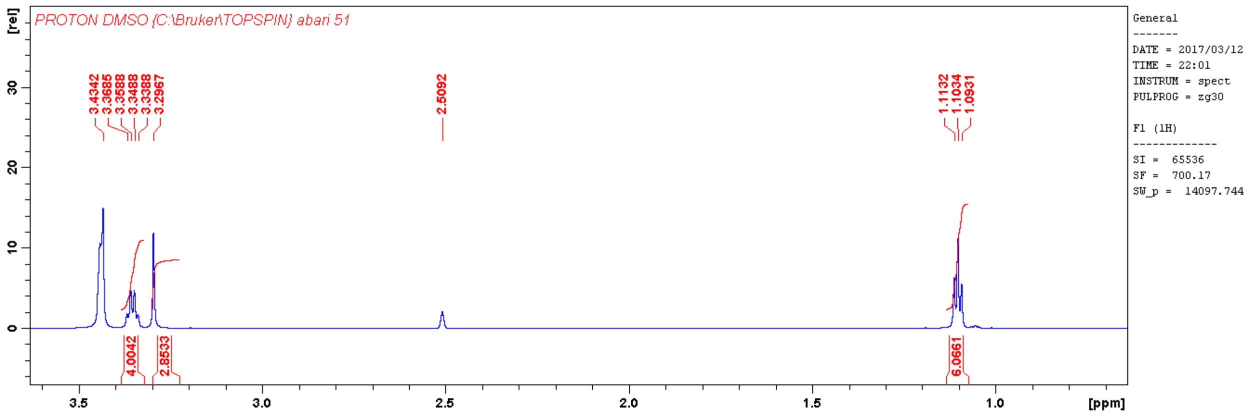
PROTON DMSO {C:\Bruker\TOPSPIN} abari 51
3.4342
3.3685
3.3588
3.3488
3.3388
3.2967
2.5092
1.1132
1.1034
1.0931
4.0042
2.8533
6.0661
[ppm]
General
DATE = 2017/03/12
TIME = 22:01
INSTRUM = spect
PULPROG = zg30
F1 (1H)
SI = 65536
SF = 700.17
SW_p = 14097.744

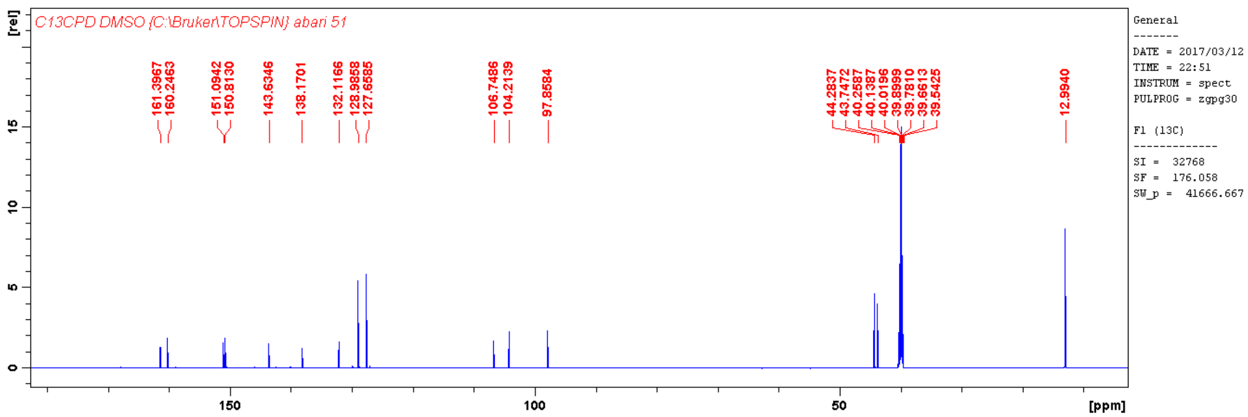
C13CPD DMSO {C:\Bruker\TOPSPIN} abari 51
161.3967
160.2463
151.0942
150.8130
143.6346
138.1701
132.1166
128.9858
127.6585
106.7486
104.2139
97.8584
44.2837
43.7472
40.2587
40.1387
40.0196
39.8999
39.7810
39.6613
39.5425
12.9940
[ppm]
General
DATE = 2017/03/12
TIME = 22:51
INSTRUM = spect
PULPROG = zgpg30
F1 (13C)
SI = 32768
SF = 176.058
SW_p = 41666.667

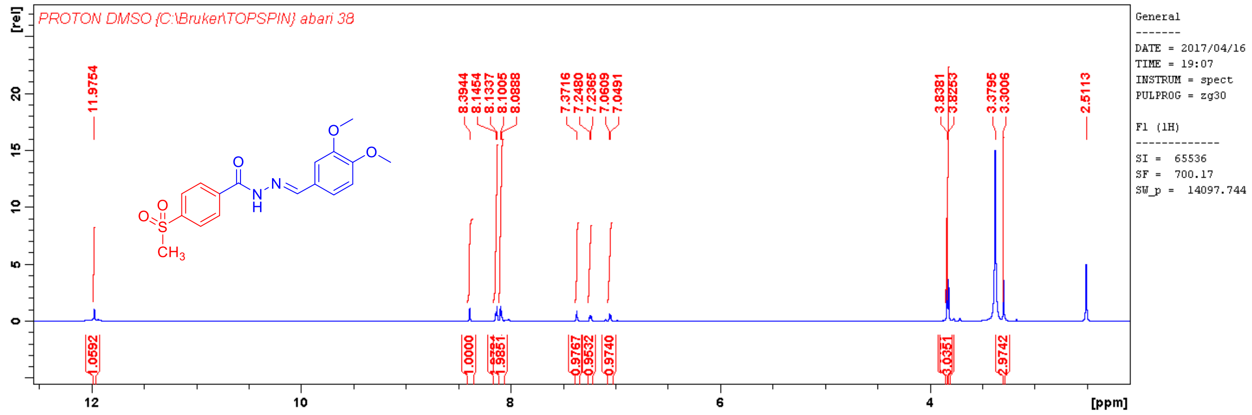
PROTON DMSO {C:\Bruker\TOPSPIN} abari 38
11.9754
8.3944
8.1454
8.1337
8.1005
8.0888
7.3716
7.2480
7.2365
7.0609
7.0491
3.8381
3.8253
3.3795
3.3006
2.5113
1.0592
1.0000
1.9784
1.9851
0.9767
0.9532
0.9740
3.0351
2.9742
O
S
O
CH3
O
N
H
N
O
O
General
DATE = 2017/04/16
TIME = 19:07
INSTRUM = spect
PULPROG = zg30
F1 (1H)
SI = 65536
SF = 700.17
SW_p = 14097.744
[rel]
[ppm]

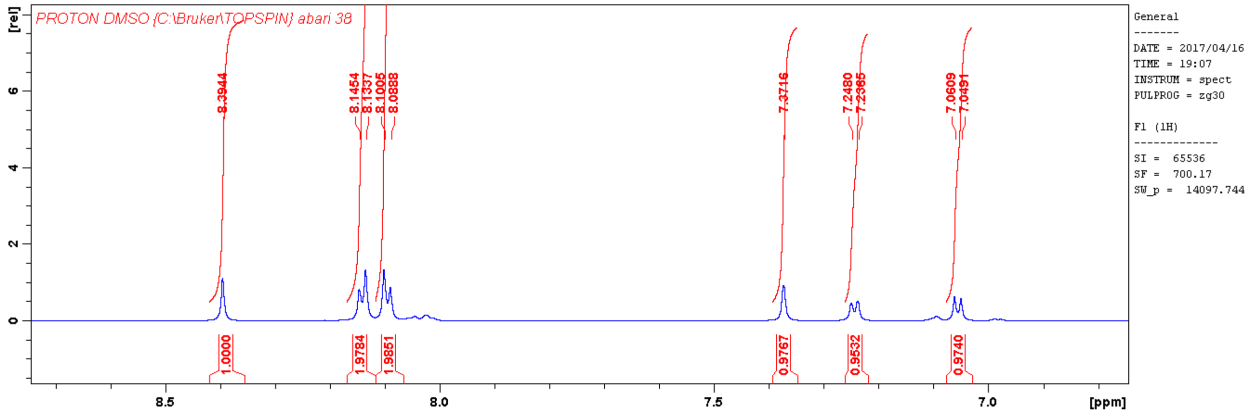
PROTON DMSO {C:\Bruker\TOPSPIN} abari 38
8.3944
8.1454
8.1337
8.1005
8.0888
7.3716
7.2480
7.2365
7.0609
7.0491
1.0000
1.9784
1.9851
0.9767
0.9532
0.9740
General
DATE = 2017/04/16
TIME = 19:07
INSTRUM = spect
PULPROG = zg30
F1 (1H)
SI = 65536
SF = 700.17
SW_p = 14097.744
[rel]
[ppm]

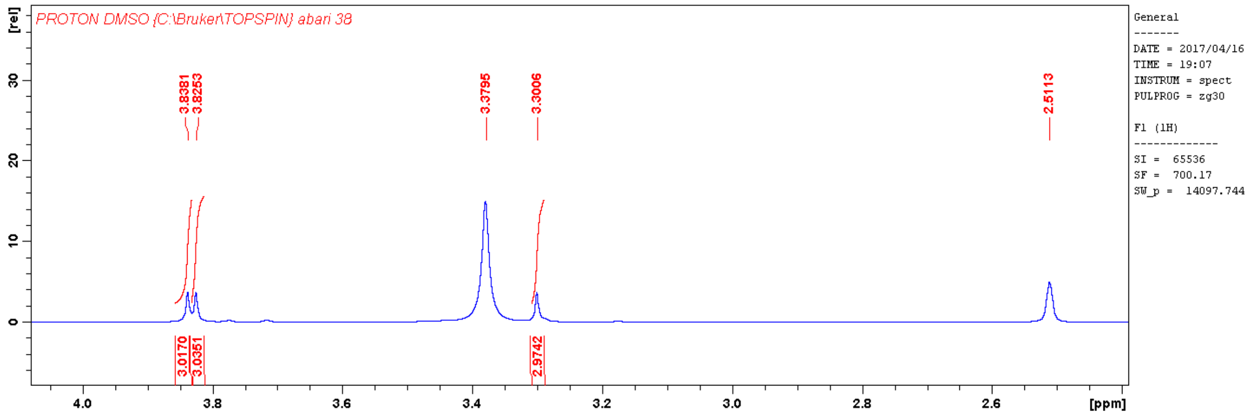
PROTON DMSO {C:\Bruker\TOPSPIN} abari 38
3.8381
3.8253
3.3795
3.3006
2.5113
3.0170
3.0351
2.9742
General
DATE = 2017/04/16
TIME = 19:07
INSTRUM = spect
PULPROG = zg30
F1 (1H)
SI = 65536
SF = 700.17
SW_p = 14097.744
[rel]
[ppm]

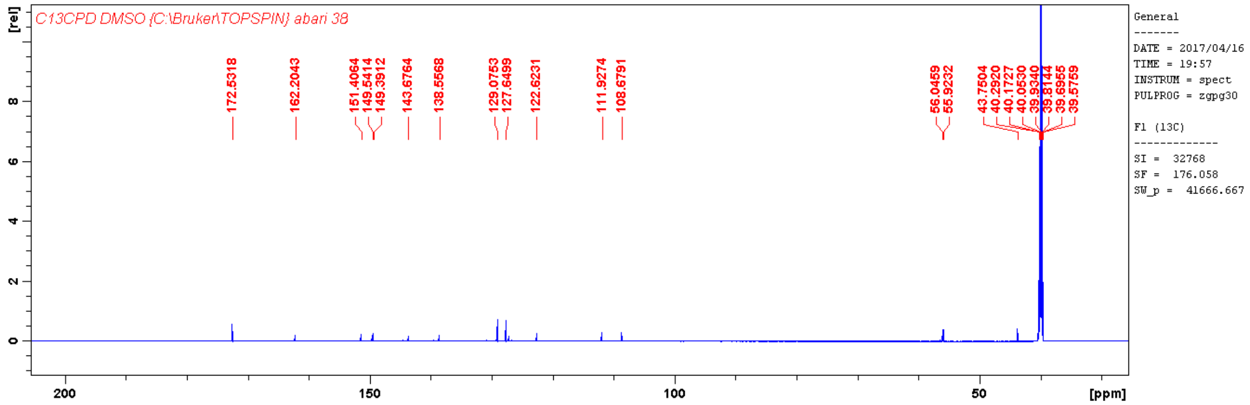
C13CPD DMSO {C:\Bruker\TOPSPIN} abari 38
172.5318
162.2043
151.4064
149.5414
149.3912
143.6764
138.5568
129.0753
127.6499
122.6231
111.9274
108.6791
56.0459
55.9232
43.7504
40.2920
40.1727
40.0530
39.9340
39.8144
39.6955
39.5759
General
DATE = 2017/04/16
TIME = 19:57
INSTRUM = spect
PULPROG = zgpg30
F1 (13C)
SI = 32768
SF = 176.058
SW_p = 41666.667
[rel]
[ppm]

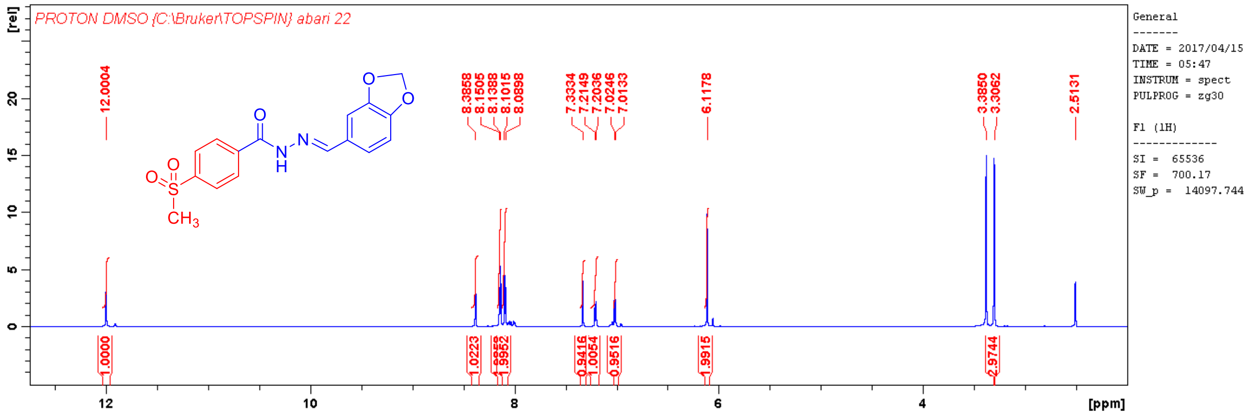
PROTON DMSO {C:\Bruker\TOPSPIN} abari 22
12.0004
8.3858
8.1505
8.1388
8.1015
8.0898
7.3334
7.2149
7.2036
7.0246
7.0133
6.1178
3.3850
3.3062
2.5131
1.0000
1.0223
1.9858
1.9952
0.9416
1.0054
0.9516
1.9915
2.9744
[rel]
[ppm]
General
DATE = 2017/04/15
TIME = 05:47
INSTRUM = spect
PULPROG = zg30
F1 (1H)
SI = 65536
SF = 700.17
SW_p = 14097.744

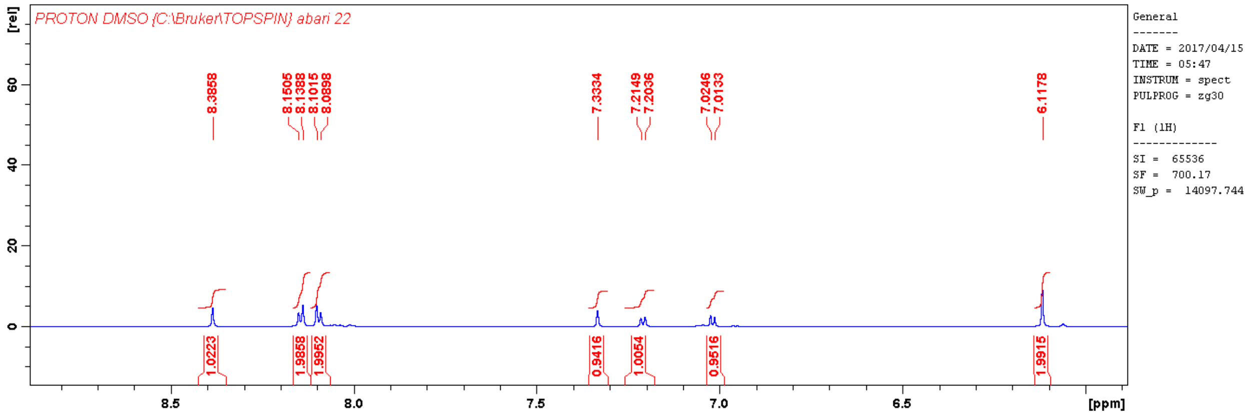
PROTON DMSO {C:\Bruker\TOPSPIN} abari 22
8.3858
8.1505
8.1388
8.1015
8.0898
7.3334
7.2149
7.2036
7.0246
7.0133
6.1178
1.0223
1.9858
1.9952
0.9416
1.0054
0.9516
1.9915
[rel]
[ppm]
General
DATE = 2017/04/15
TIME = 05:47
INSTRUM = spect
PULPROG = zg30
F1 (1H)
SI = 65536
SF = 700.17
SW_p = 14097.744

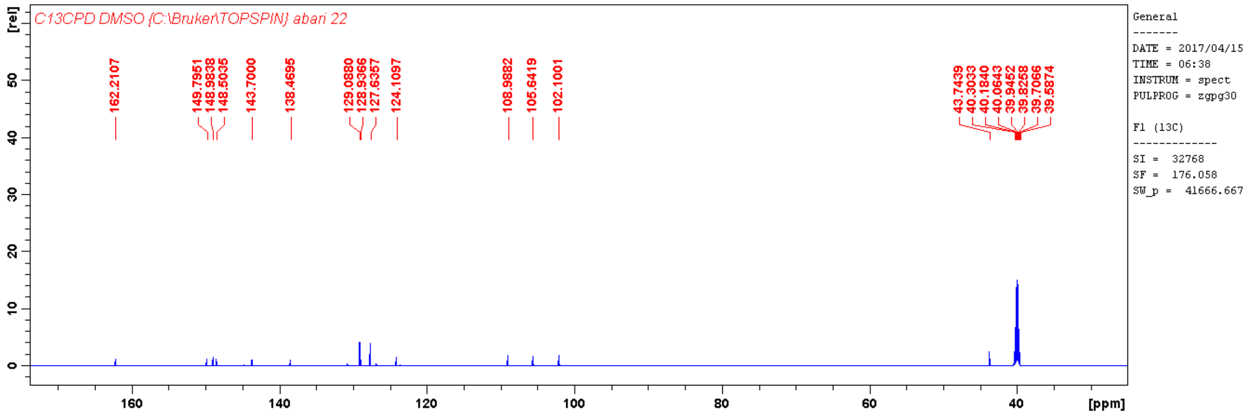
C13CPD DMSO {C:\Bruker\TOPSPIN} abari 22
162.2107
149.7951
148.9838
148.5035
143.7000
138.4695
129.0880
128.9366
127.6357
124.1097
108.9882
105.6419
102.1001
43.7439
40.3033
40.1840
40.0643
39.9452
39.8258
39.7066
39.5874
[rel]
[ppm]
General
DATE = 2017/04/15
TIME = 06:38
INSTRUM = spect
PULPROG = zgpg30
F1 (13C)
SI = 32768
SF = 176.058
SW_p = 41666.667

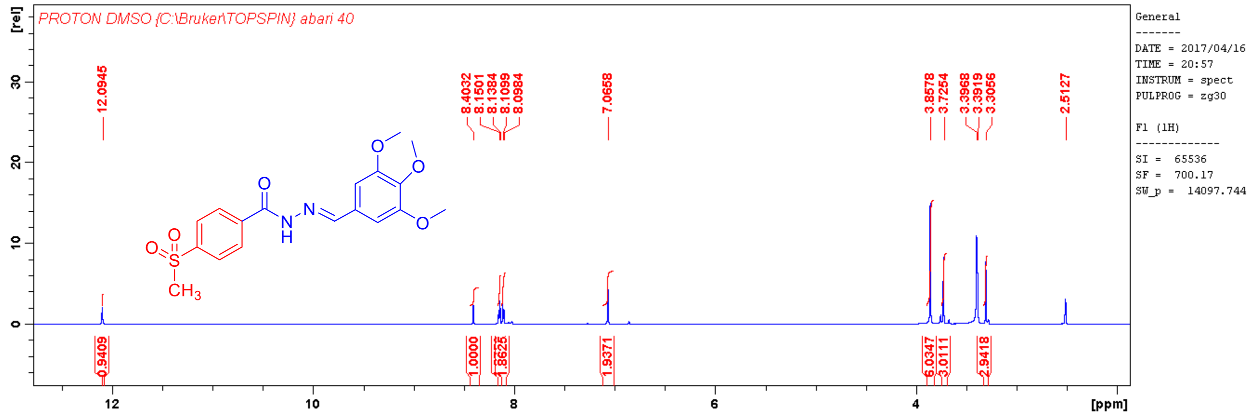
PROTON DMSO {C:\Bruker\TOPSPIN} abari 40
12.0945
8.4032
8.1501
8.1384
8.1099
8.0984
7.0658
3.8578
3.7254
3.3968
3.3919
3.3056
2.5127
0.9409
1.0000
1.8625
1.9371
6.0347
3.0111
2.9418
[rel]
[ppm]
General
DATE = 2017/04/16
TIME = 20:57
INSTRUM = spect
PULPROG = zg30
F1 (1H)
SI = 65536
SF = 700.17
SW_p = 14097.744

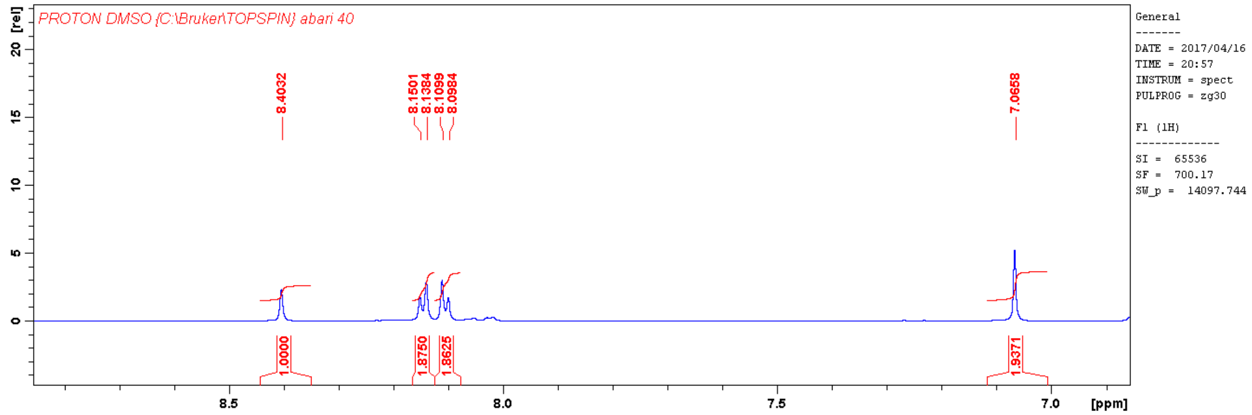
PROTON DMSO {C:\Bruker\TOPSPIN} abari 40
8.4032
8.1501
8.1384
8.1099
8.0984
7.0658
1.0000
1.8750
1.8625
1.9371
[rel]
[ppm]
General
DATE = 2017/04/16
TIME = 20:57
INSTRUM = spect
PULPROG = zg30
F1 (1H)
SI = 65536
SF = 700.17
SW_p = 14097.744

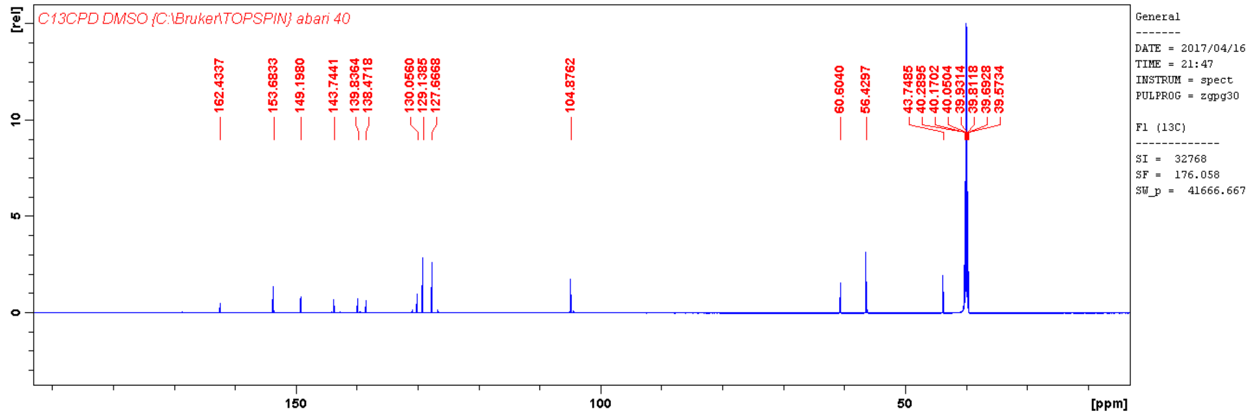
C13CPD DMSO {C:\Bruker\TOPSPIN} abari 40
162.4337
153.6833
149.1980
143.7441
139.8364
138.4718
130.0560
129.1385
127.6668
104.8762
60.6040
56.4297
43.7485
40.2895
40.1702
40.0504
39.9314
39.8118
39.6928
39.5734
[rel]
[ppm]
General
DATE = 2017/04/16
TIME = 21:47
INSTRUM = spect
PULPROG = zgpg30
F1 (13C)
SI = 32768
SF = 176.058
SW_p = 41666.667

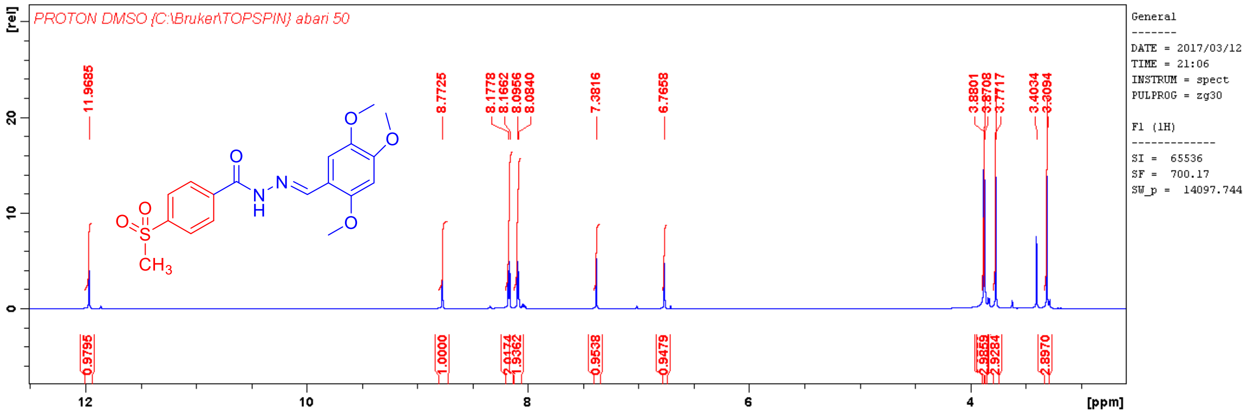
PROTON DMSO {C:\Bruker\TOPSPIN} abari 50
11.9685
8.7725
8.1778
8.1662
8.0956
8.0840
7.3816
6.7658
3.8801
3.8708
3.7717
3.4034
3.3094
0.9795
1.0000
2.0174
1.9362
0.9538
0.9479
2.9859
2.9284
2.8970
[ppm]
[rel]
General
DATE = 2017/03/12
TIME = 21:06
INSTRUM = spect
PULPROG = zg30
F1 (1H)
SI = 65536
SF = 700.17
SW_p = 14097.744

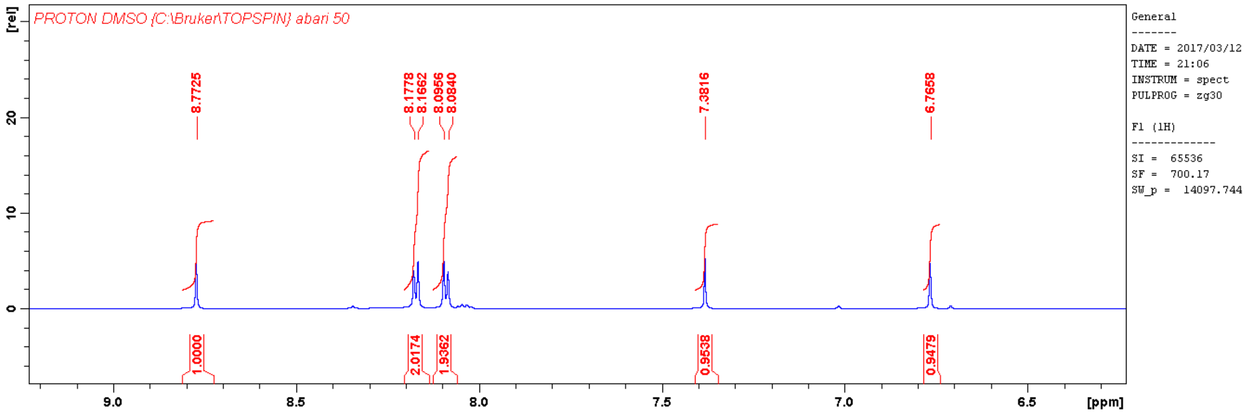
PROTON DMSO {C:\Bruker\TOPSPIN} abari 50
8.7725
8.1778
8.1662
8.0956
8.0840
7.3816
6.7658
1.0000
2.0174
1.9362
0.9538
0.9479
[ppm]
[rel]
General
DATE = 2017/03/12
TIME = 21:06
INSTRUM = spect
PULPROG = zg30
F1 (1H)
SI = 65536
SF = 700.17
SW_p = 14097.744

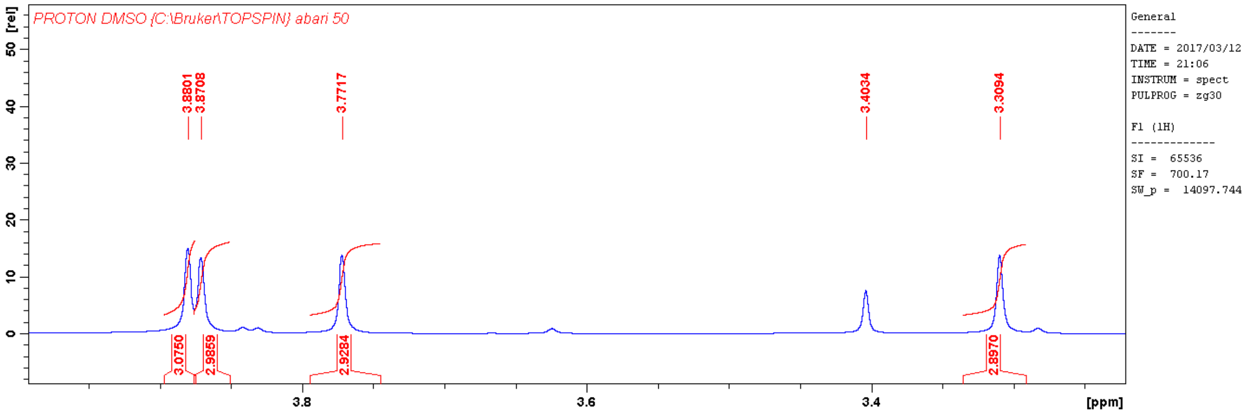
PROTON DMSO {C:\Bruker\TOPSPIN} abari 50
3.8801
3.8708
3.7717
3.4034
3.3094
3.0750
2.9859
2.9284
2.8970
[ppm]
[rel]
General
DATE = 2017/03/12
TIME = 21:06
INSTRUM = spect
PULPROG = zg30
F1 (1H)
SI = 65536
SF = 700.17
SW_p = 14097.744

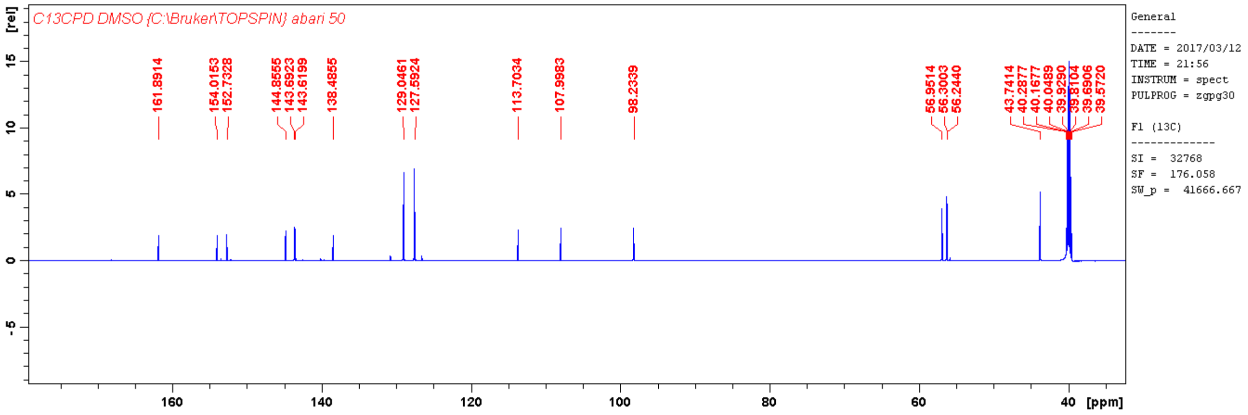
C13CPD DMSO {C:\Bruker\TOPSPIN} abari 50
161.8914
154.0153
152.7328
144.8555
143.6923
143.6199
138.4855
129.0461
127.5924
113.7034
107.9983
98.2339
56.9514
56.3003
56.2440
43.7414
40.2877
40.1677
40.0489
39.9290
39.8104
39.6906
39.5720
[ppm]
[rel]
General
DATE = 2017/03/12
TIME = 21:56
INSTRUM = spect
PULPROG = zgpg30
F1 (13C)
SI = 32768
SF = 176.058
SW_p = 41666.667

2. Materials and methods

2.1. Biological evaluation

2.1.1. *In vitro* antitumor screening

The antitumor assay was performed on approximately 59 human tumor cell lines obtained from nine organs following the protocol of the Drug Evaluation Branch, National Cancer Institute, Bethesda, MD. Three dose-response parameters: GI50, TGI, and LC50, were calculated for each compound. Initially, the tested compounds were added to the culture at a single concentration (10 mM), and the cultures were incubated for 48 h. Endpoint determinations were made with a protein-binding dye, sulforhodamine B (SRB). The results for each tested compound were reported as the growth percentage of the treated cells compared to those of the untreated control cells. The percentage of growth was evaluated spectrophotometrically against controls not treated with test agents. The growth inhibitory effects of the most active compounds were tested in vitro against the full panel of approximately 60 human tumor cell lines at 10-fold dilutions of five concentrations, ranging from 10-4 to 10-8 M. A 48 h continuous drug exposure protocol was followed, and an SRB protein assay was used to estimate cell viability or growth. Using the seven absorbance measurements [time zero, (Tz), control growth in the absence of drug (C), and test growth in the presence of drug at the five concentration levels (Ti)], the percentage growth was calculated at each the drug concentrations levels. Percentage growth inhibition was calculated as $[(T_i-T_z)/(C-T_z) \times 100]$ for concentration of which $T_i \geq T_z$,

$[(T_i-T_z)/T_z)] \times 100$ for concentration of which $T_i \leq T_z$.

2.1.2. Apoptosis assay

The induction of apoptosis was performed according to our previous report using Leukemia HL-60 cell line and well-established Annexin 5-FITC/PI detection kit. The cell line samples were analyzed using a FACSCalibur flow cytometer.

2.1.3. Cell cycle analysis

Using a method similar to that of our previous report, cell cycle analysis was carried out using Leukemia HL-60 cell line, stained with the DNA fluorochrome PI, and analyzed by FACSCalibur flow cytometer. Leukemia HL-60 cell was seeded at a density of 2 × 105 cells/well and incubated for 24 h in six-well plates. Fetal bovine serum (10%) was added, and the cells were incubated at 37°C in an atmosphere of 5% CO2. The medium was replaced with 1% (v/v) dimethyl sulfoxide (DMSO), containing 10.0 μM of compound **9**. Next, the cells were incubated for 48 h, washed with phosphate-buffered saline, fixed with 70% ethanol, rinsed with phosphate-buffered saline, and then stained with the DNA fluorochrome PI for 15 min at 37°C. Afterward, the samples were analyzed using a FACSCalibur flow cytometer.

2.1.4. *In vitro* cyclooxygenase (COX) inhibition assay

The colorimetric COX-2 inhibition assay (kit catalog number 560101, Cayman Chemical, Ann Arbour, MI) was used to measure the ability of the tested derivatives, and celecoxib was used to inhibit COX-2 isozyme following the manufacturer's instructions. The tested compounds' ability to inhibit ovine COX-2 was determined using an enzyme immunoassay (EIA) (kit catalog number 560101, Cayman Chemical, Ann Arbor, MI, USA) according to the manufacturer's instructions. Cyclooxygenase catalyzes the first reaction in the biosynthesis of arachidonic acid (AA) to PGH2. PGF2a, produced from PGH2 by reduction with stannous chloride, was measured by an enzyme immunoassay (ACE™ competitive EIA). Stock solutions of the tested compounds were dissolved in a minimum volume of DMSO. Subsequently, 10 μl of various concentrations of test drug solutions (0.01, 0.1, 1, 10, 50, and 100 lM in a final volume of 1 mL) were added to a series of supplied reaction buffer solutions (960 μl, 0.1 MTris–HCl pH 8.0, containing 5 mM EDTA and 2 mM phenol) with either COX-1 or COX-2 (10 μl) enzyme in the presence of heme (10 μl) for 5 min at 37°C; after which 10 μl of AA (100 lM) solution was added, and the COX reaction was stopped by the addition of 50 μl of 1 M HCl after 2 min. PGF2a, produced from PGH2 by reduction with stannous chloride, was measured by enzyme immunoassay. This assay was based on the competition between PGs and a PG-acetylcholinesterase conjugate (PG tracer) for a limited amount of PG antiserum. The PG tracer amount that can bind to the PG antiserum is inversely proportional to the concentration of the PGs in the wells since the concentration of PG tracer is held constant while the concentration of PGs varies. This antibody, PG complex, binds to a mouse anti-rabbit monoclonal antibody that

had been previously attached to the well. The plate was washed to remove any unbound reagents, and then Ellman's reagent, containing the substrate of acetylcholine esterase, was added to the well. This enzymatic reaction produced a distinct yellow color that was absorbed in 410 nm. The intensity of the color, determined spectrophotometrically, was proportional to the amount of PG tracer bound to the well, which was inversely proportional to PGs present in the well during the incubation: Absorbance ∝ [Bound PG Tracer] a1/PGs. Percent inhibition was calculated by comparing the treated compound to various control incubations. The test compound concentration, causing 50% inhibition (IC50, lM), was calculated from the concentration-inhibition response curve (duplicate determinations).

2.1.5. EGFR and Her2 assays

In vitro luminescent EGFR tyrosine kinase assay, using Kinase-Glo® MAX as a detection reagent, and *In vitro* HER2 tyrosine kinase assay, using DP-Glo™ reagent that measures the adenosine diphosphate (ADP) formed from a kinase reaction, were performed; this luminescent signal positively correlates with ADP amount and kinase activity. In 17 µl of distilled water, 6 µl of Kinase assay buffer and 1 µl ATP and 1 µl PTK substrate were mixed (master mixture). In every well, mix 20 µl of the master mixture and 5 µl of Inhibitor solution (Test Inhibitor) for positive control, and use 5 µl of the same solution without inhibitor (Inhibitor buffer) as the blank solution; add 20 µl of diluted EGFR or Her2 enzyme and incubate at 30°C for 40 min. Add 50 µl of Kinase-Glo Max reagent to each well, cover the plate with aluminum foil and incubate the plate at room temperature for 15 min. Afterward, measure luminescence using the microplate reader. "Blank" value is subtracted from all readings. All samples and controls should be tested in duplicates.

2.2. Molecular docking method

Molecular docking protocols were carried out using the MOE 2008.10 software from Chemical Computing Group Inc. (Montreal, QC, Canada) following previously established methods. The crystal structures of COX-2 (PDB code: 1CX2), EGFR (PDB Code: 1M17), and HER2 (PDB Code: 3PP0) were retrieved from the protein data bank. The SwissTargetPrediction and the

SwissADME online tools were used to predict the test compounds' physicochemical, pharmacokinetics, and drug-likeness properties and used reference drugs.

2.2.1. Selection of protein crystal structure

The ligand-bound crystallographic structures of COX-2, EGFR, and Her2 are available in the Protein Data Bank (http://www.rcsb.org/). In this study, COX-2 (PDB Code: 1CX2), EGFR (PDB code: 1M17), and Her2 (PDB code: 3PP0) with bound inhibitors were evaluated and selected for docking. The structure preparation process in MOE corrected the errors of the protein. The first step in the generation of suitable protein structures is the assignment of hydrogen positions based on default rules. All bound waters and cofactors contained in the PDB file were removed. Finally, partial charges (the Gasteiger methodology) were calculated, and the active site of the ensemble was defined as the collection of residues within 10.0 Å of the bound inhibitors and comprised the union of all the ligands in the ensemble. All atoms located less than 10.0 Å from any ligand atom were considered.

2.2.2. Preparation of the ligand

The ligand coordinates were built using the builder tool of the MOE program. Next, the correct atom types (including hybridization states) and correct bond types were defined, hydrogen atoms were added, charges were assigned to each atom, and finally, the energies of the structures were minimized (MMFF94x, gradient: 0.01). The energies of the ligand structures were previously minimized using the semi-empirical AM1 method with MOE program; point charges were assigned to the ligands.

2.2.3. Docking experiment

The docking experiments on COX-2, EGFR, and Her2 were carried out by superimposing the energy-minimized ligand on bound inhibitors in the PDB files; after which, co-crystallized inhibitors were deleted. The default Triangle Matcher placement method was used for docking. GBVI/WSA dG scoring function, which estimates the ligand's free binding energy from a given pose, was used to rank the final poses. The geometry of the resulting complexes was studied

using the MOE's Pose Viewer utility. The ligand-enzyme complex with thelowest S score was selected.

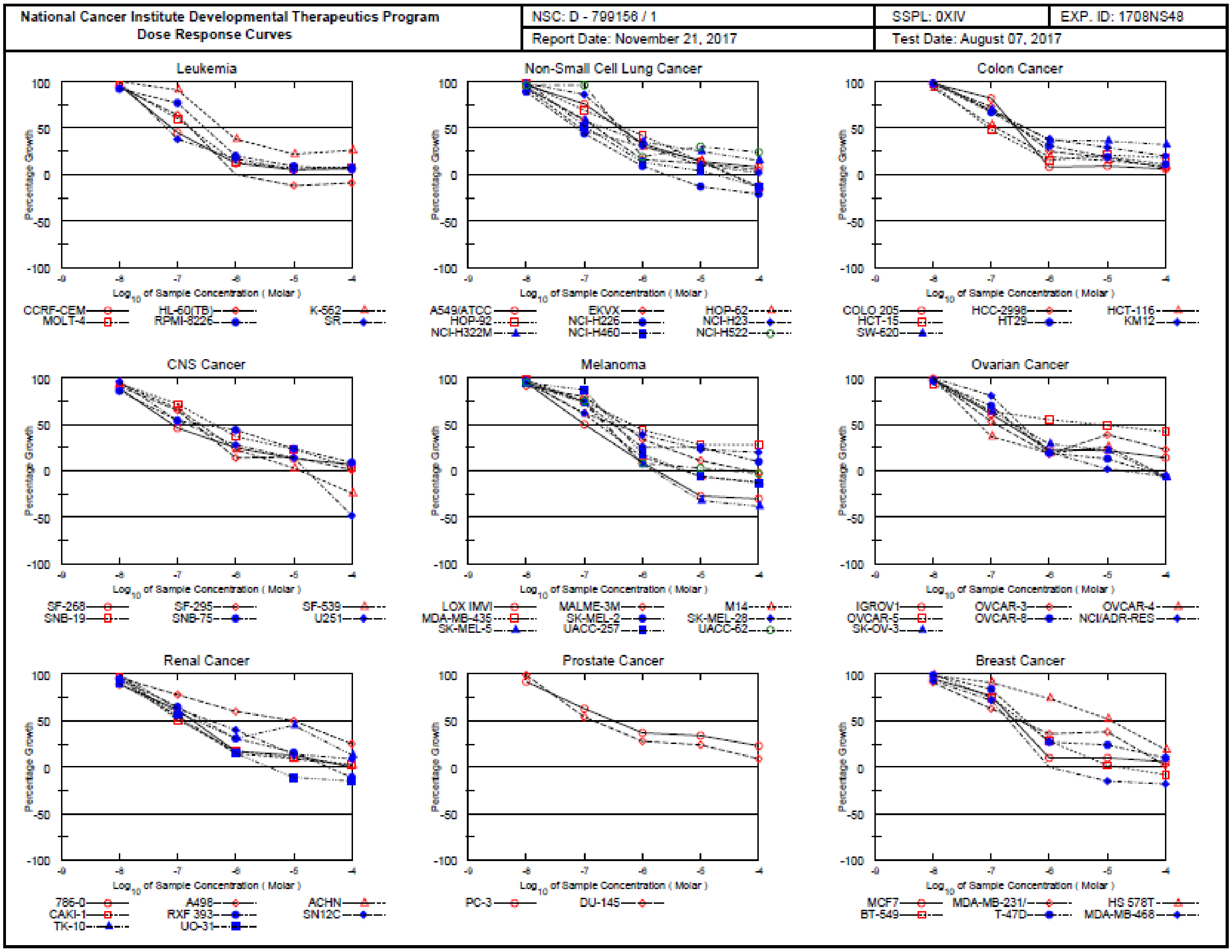

Figure S1. NCI Dose-Response Curves for compound **20**.
